# IGF2 interacts with the imprinted gene *Cdkn1c* to promote terminal differentiation of neural stem cells

**DOI:** 10.1242/dev.200563

**Published:** 2023-01-12

**Authors:** Anna Lozano-Ureña, Laura Lázaro-Carot, Esteban Jiménez-Villalba, Raquel Montalbán-Loro, Isabel Mateos-White, Pere Duart-Abadía, Irene Martínez-Gurrea, Keiichi I. Nakayama, Isabel Fariñas, Martina Kirstein, Cristina Gil-Sanz, Sacri R. Ferrón

**Affiliations:** ^1^ Instituto de Biotecnología y Biomedicina (BIOTECMED), Universidad de Valencia, Valencia 46100, Spain; ^2^ Departamento de Biología Celular, Universidad de Valencia, Valencia 46100, Spain; ^3^ Department of Molecular and Cellular Biology, Medical Institute of Bioregulation, Kyushu University, Fukuoka 819-0395, Japan

**Keywords:** Genomic Imprinting, Insulin-like growth factor 2, Neural Stem Cells, Neurosphere cultures, p57

## Abstract

Adult neurogenesis is supported by multipotent neural stem cells (NSCs) with unique properties and growth requirements. Adult NSCs constitute a reversibly quiescent cell population that can be activated by extracellular signals from the microenvironment in which they reside *in vivo*. Although genomic imprinting plays a role in adult neurogenesis through dose regulation of some relevant signals, the roles of many imprinted genes in the process remain elusive. Insulin-like growth factor 2 (IGF2) is encoded by an imprinted gene that contributes to NSC maintenance in the adult subventricular zone through a biallelic expression in only the vascular compartment. We show here that IGF2 additionally promotes terminal differentiation of NSCs into astrocytes, neurons and oligodendrocytes by inducing the expression of the maternally expressed gene cyclin-dependent kinase inhibitor 1c (*Cdkn1c*), encoding the cell cycle inhibitor p57. Using intraventricular infusion of recombinant IGF2 in a conditional mutant strain with *Cdkn1c*-deficient NSCs, we confirm that p57 partially mediates the differentiation effects of IGF2 in NSCs and that this occurs independently of its role in cell-cycle progression, balancing the relationship between astrogliogenesis, neurogenesis and oligodendrogenesis.

## Teaser

**Summary:** Intraventricular infusion of recombinant insulin-like growth factor 2 (IGF2) in a *Cdkn1c*-deficient mouse, reveals that p57 partially mediates the differentiation effects of IGF2 independently of its role in cell-cycle progression.

## INTRODUCTION

Adult neurogenesis in the subventricular zone (SVZ) is an orderly multistep process in which self-renewing astrocyte-like neural stem cells (NSCs or B1 cells) produce mature progeny via transit-amplifying progenitors (TAPs or C cells) (Chaker et al., 2016). These progenitors rapidly divide to give rise to neuroblasts (A cells) that migrate through the rostral migratory stream (RMS) to the olfactory bulb (OB) where they fully differentiate and integrate as interneurons (Calzolari et al., 2015; Chaker et al., 2016; Götz et al., 2016; Ponti et al., 2013). The SVZ also gives rise to astrocytes and oligodendrocytes that integrate into the corpus callosum (CC) (Menn et al., 2006; Sohn et al., 2015) and striatum (Figueres-Oñate et al., 2019). Sustained neurogenesis throughout adult life also occurs in the subgranular zone (SGZ) of the dentate gyrus (DG) in the hippocampus and is hypothesized to be involved in behavioural/cognitive processes, such as memory, and in diseases of the central nervous system (CNS) (Gage et al., 1998; Gonçalves et al., 2016).

Genomic imprinting is an epigenetic process that causes genes to be expressed depending on their parental origin (John and Surani, 2000). A relatively small subset of genes within the mammalian genome (0.4%) is imprinted, showing monoallelic expression in the whole organism or in specific tissues favouring the maternal or the paternal allele (John and Surani, 2000). Imprinted expression is initially determined by differential DNA methylation that is established in the germline (Surani, 1998). The prevalence of genomic imprinting is higher in the brain than in other organs and many genes with a crucial role in neurodevelopment are expressed in a maternal- or paternal-specific manner (Perez et al., 2016). For example, *Cdkn1c* is an imprinted gene expressed only by the maternal allele (Pateras et al., 2009) and the misregulation of its expression has been associated with human growth disorders, such as Beckwith-Wiedemann and Silver-Russell syndromes, as well as with the onset of several types of cancers (Bastaki et al., 2016; Binder et al., 2020; Nakashima et al., 2015; Stampone et al., 2021). *Cdkn1c* encodes p57 protein and belongs to the CIP/KIP family of cyclin-dependent kinase inhibitors (CKIs), which also includes p21 and p27. Among them, p57 is the least studied CIP/KIP member, although it has been demonstrated to play a fundamental role in regulating the cell cycle and differentiation during mammalian development (Rossi et al., 2018). Indeed, this cell cycle regulator controls multiple stages of corticogenesis such as cell cycle exit of foetal progenitors, their differentiation and migration (Furutachi et al., 2015; Jadasz et al., 2012; Laukoter et al., 2020; Mairet-Coello et al., 2012; Tury et al., 2011). Importantly, p57 accumulates in proliferating MASH1^+^ telencephalic neural progenitors; it represses neuronal differentiation independently of cell-cycle exit and acts as a direct repressor of transcription (Joseph et al., 2009). p57 has also been shown to be a key factor during embryonic specification of quiescent NSCs and its deletion impairs the emergence of adult NSCs in the SVZ (Furutachi et al., 2015). Moreover, p57 inhibits adult NSC proliferation after focal cerebral ischemia (Guo et al., 2014) and controls NSC quiescence in the adult SGZ (Furutachi et al., 2013). Consistently, a detailed analysis of hematopoietic stem cells (HSCs) lacking p57 has confirmed the role of this cell cycle inhibitor as a key molecule in quiescence and self-renewal (Matsumoto et al., 2011; Tesio and Trumpp, 2011; Zou et al., 2011).

*Igf2* (insulin-like growth factor 2) is another imprinted gene expressed only by the paternal allele in most tissues (DeChiara et al., 1991; Ferguson-Smith et al., 1991; Giannoukakis et al., 1993). Remarkably, the silent maternal allele is specifically activated in neurogenic niches, resulting in biallelic expression of the gene (Ferrón et al., 2015; Lehtinen et al., 2011). It has been shown that IGF2 regulates NSCs in the adult mouse SVZ by influencing their cell cycle (Ferrón et al., 2011; Lehtinen et al., 2011; Ziegler et al., 2019). Indeed, IGF2 has been shown to promote expansion of NSCs more potently than either insulin growth factor 1 (IGF1) or standard growth media (Alagappan et al., 2014; Ziegler et al., 2014, 2019). Detailed analysis of the SVZ niche has shown that IGF2 is secreted in a paracrine manner by the choroid plexus (CP) epithelium, and becomes readily accessible to those NSCs contacting the cerebrospinal fluid (CSF) (Bracko et al., 2012; Ferrón et al., 2015; Lehtinen et al., 2011; Ziegler et al., 2014). The brain vasculature and leptomeninges also secrete IGF2 into the neurogenic niches (Ferrón et al., 2015). Accordingly, studies in the hematopoietic system showed that IGF2 acts as a potent growth factor for adult bone marrow cells by stimulating their *ex vivo* expansion (Barroca et al., 2017; Zhang and Lodish, 2004), and a direct link between IGF2 and the cell cycle regulator p57 has been shown in this system (Thomas et al., 2016). However the mechanisms by which IGF2 governs neurogenesis remain incompletely characterized.

In our study, we report that maternal, but not paternal, deletion of *Cdkn1c* in adult NSCs abrogates their quiescence and promotes their premature differentiation into terminally differentiated astrocytes. Moreover, we show that IGF2 promotes cell fate commitment by inducing the expression of *Cdkn1c* mRNA and p57 protein, which elicits cell cycle exit and enhances terminal differentiation of adult NSCs into non-multipotent astrocytes. Consistently, intraventricular infusion of recombinant IGF2 in a conditional mouse model with *Cdkn1c*-deficient neural progenitors has confirmed that IGF2 and p57 act in a common pathway to regulate quiescence and differentiation of the NSC pool in the adult SVZ. We also identify a methylation-independent mechanism of control of the *Cdkn1c* imprinted gene after IGF2 treatment, which involves the PI3K-Akt pathway during the differentiation of adult NSCs.

## RESULTS

### IGF2 promotes terminal differentiation of NSCs *in vitro*

**Fig. 1.**
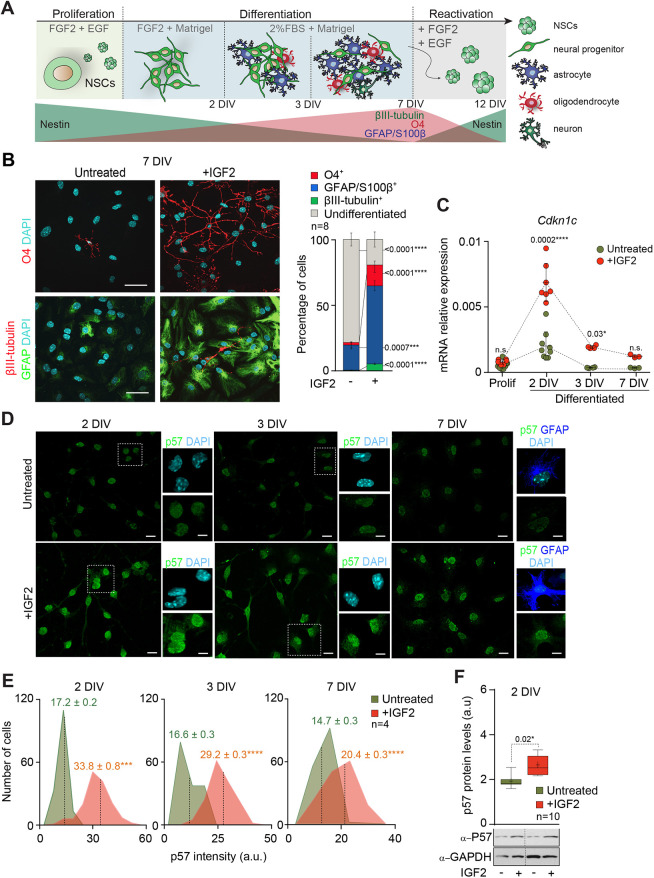
**IGF2 promotes terminal differentiation of adult NSCs.** (A) Schematic representation of differentiation and reactivation protocols in adult NSCs. For expansion, cells were grown in presence of mitogens (EGF and FGF) and then cultures were attached to Matrigel in absence of EGF for 2 days. For terminal differentiation, FGF was removed from the medium and NSCs were cultured for 5 more days in medium with serum. For reactivation, NSCs were detached and cultured again in proliferation-promoting conditions. (B) Immunocytochemistry images for O4 (red), GFAP (green) and βIII-tubulin (red) in NSCs after 7 DIV under differentiation conditions in the presence or absence of IGF2 (left panel). Percentage of cells positive for βIII-tubulin, O4 or GFAP/S100β in untreated and in IGF2-treated cultures after 7 DIV of differentiation. The percentage of undifferentiated cells is also determined (right panel). Data are mean±s.e.m.; *n*=8 experimental replicates (two-tailed paired Student's *t*-test). (C) Quantitative PCR (qPCR) for *Cdkn1c* in proliferating NSCs and after 2, 3 and 7 DIV in differentiation-promoting conditions in the absence (green) or presence of IGF2 (red). *Gapdh* was used as a housekeeping gene. Data are mean±s.e.m.; *n*=9, 9, 4 and 3 experimental replicates in untreated conditions and 6, 7, 4 and 3 experimental replicates in IGF2-treated conditions (Mann–Whitney test). (D) Immunocytochemistry images for p57 (green) in NSCs after 2, 3 and 7 days of differentiation in the presence or absence of IGF2. Images for GFAP (blue) are also shown in NSCs after 7 days of differentiation. (E) Histograms showing p57 intensity (in arbitrary units, a.u.) in NSC cultures in the presence or absence of IGF2 after 2, 3 and 7 DIV under differentiation conditions. Mean intensities are indicated as dashed lines. Data are mean±s.e.m; *n*=4 experimental replicates (two-tailed paired Student's *t*-test). (F) Western-blot of p57 after 2 DIV under differentiation conditions in untreated and IGF-treated cultures (lower panel). Quantification of p57 protein levels by western blot (upper panel). Boxes indicate interquartile range and whiskers indicate maximum and minimum values; *n*=10 experimental replicates (two-tailed paired Student's *t*-test). DAPI was used to counterstain DNA. *P*-values and number of samples are indicated. Scale bars: 30 µm in B,D; 6 µm in high magnification images in D.

IGF2 plays an important role in NSCs by affecting their proliferation capacity (Bracko et al., 2012; Ferrón et al., 2015; Lehtinen et al., 2011); however, little is known about the effects of IGF2 in the differentiation process of NSCs. In order to study the effects of this factor in neural differentiation, NSCs isolated from the adult SVZ were expanded and differentiated into the three neural lineages of the CNS in the presence or absence of IGF2 (Belenguer et al., 2016). To do this, cells were first expanded as neurospheres in the presence of mitogenic stimulation with epidermal growth factor (EGF) and fibroblast growth factor 2 (FGF2) (Ferrón et al., 2007). Neurospheres were then disaggregated, plated on Matrigel (adherent assay) and cultured for 2 days *in vitro* (2 DIV) in medium without any insulin stimulation but containing FGF2 to induce neural progenitors differentiation (Belenguer et al., 2016). Afterwards, mitogens were withdrawn and the medium was supplemented with 2% foetal bovine serum (FBS) which is required for terminal differentiation (Fig. 1A). Under these conditions, differentiation is pushed forward and NSCs stop dividing and initiate an orderly program of commitment and differentiation into neurons, oligodendrocytes and astrocytes during the following 5 days (7 DIV) (Fig. 1A).

After 2 DIV under differentiation conditions, IGF2-treated NSCs showed an increase in proliferation, as indicated by an MTS viability assay (Fig. S1A). Consistently, immunodetection of the proliferation marker Ki67 revealed an increase in the proportion of nestin^+^ (Nes) progenitors that were still proliferating after 2 DIV under differentiation conditions in IGF2-treated cultures (Fig. S1B). This increased rate of proliferation was maintained after 3 DIV under differentiation conditions in presence of IGF2, as revealed by a higher proportion of Ki67^+^ and MCM2^+^ cells (Fig. S1C,D). Moreover, although the level of expression of the *Nes* gene was not altered in presence of IGF2 (Fig. S2A), the expression of the neuronal gene βIII-tubulin (*Tubb3*), the astrocytic genes *S100b* (S100β) and *Gfap,* and the oligodendrocytic gene *Olig2* was increased in IGF2-treated cells after 2 DIV under differentiation conditions (Fig. S2A). Accordingly, culturing NSCs in the presence of IGF2 promoted their terminal differentiation into the three neural lineages, increasing the percentage of neurons measured as βIII-tubulin^+^ cells, astrocytes measured as cells expressing high levels of GFAP and S100β, and oligodendrocytes measured as O4^+^ cells formed after 7 DIV under differentiation conditions (Fig. 1B). Moreover, higher proportions of cells that were strongly positive for S100β, a protein largely absent from neurogenic GFAP^+^ cells (Raponi et al., 2007), and lower proportions of Nes^+^ cells, were also observed after 7 DIV under differentiation conditions in the presence of IGF2 (Fig. S2B), suggesting that IGF2 directly promotes terminal differentiation of multipotent NSCs. To confirm whether enhanced differentiation in the presence of IGF2 was accompanied by a reduction in the capacity of differentiated NSCs to form neurospheres, 7 DIV differentiated adult NSCs cultures were detached and replated again in proliferating conditions (Fig. 1A). This led to the re-activation of a small proportion of cells that retained the capacity to form neurospheres in non-adherent conditions after 5 more days (12 DIV) (Fig. S2C). As expected, the higher presence of S100β was consistent with a reduction in the neurospheres formation capacity in IGF2-treated NSCs cultures (Fig. S2C), indicating that the bias toward a more differentiated phenotype in the presence of IGF2 correlated with a reduction in stemness.

### IGF2 induces the expression of *Cdkn1c* in differentiating NSCs *in vitro*

A direct link between IGF2 and the cell cycle regulator p57 has been shown in the HSC pool (Thomas et al., 2016). In fact, overexpression of IGF2 in purified adult murine HSCs resulted in the upregulation of *Cdkn1c* expression, giving rise to an arrest of HSCs in the G_0_/G_1_ phase of the cell cycle and promoting the formation of multi-lineage colonies *in vitro*. However, the link between IGF2 and p57 in the NSC population is still unknown. Neurospheres cultures grown in suspension under proliferating conditions exhibited low levels of *Cdkn1c* mRNA, but mitogen withdrawal induced an increase in *Cdkn1c* gene expression (Fig. 1C). Moreover, a significant increase in the percentage of cells with detectable levels of the p57 protein in differentiating cultures inversely correlated with the proportion of proliferating Ki67^+^ cells (Fig. S3A), suggesting that increased expression of p57 in the absence of mitogens might correlate with cell cycle exit and differentiation of adult NSCs. Accordingly, p57 protein was detected in Nes^+^ progenitors after 2 and 3 DIV under differentiation conditions, and in GFAP^+^ astrocytes at 7 DIV (Fig. S3B).

To define the potential role of IGF2 in regulating *Cdkn1c* expression, the levels of expression of this gene was quantified by qPCR in NSCs cultures in absence or presence of IGF2. This study revealed no changes in the levels of *Cdkn1c* expression in proliferating conditions in the presence of IGF2 (Fig. 1C). However, *Cdkn1c* levels were significantly increased IGF2-treated cultures in differentiation-promoting conditions showing a maximum difference at the first step (2 DIV) of the differentiation process (Fig. 1C). No changes in the levels of expression of the other two members of the CKIs family of proteins, *Cdkn1a* and *Cdkn1b*, were observed at the same time of differentiation (Fig. S3C). Immunofluorescent detection of nuclear p57 also revealed increased levels of the p57 protein in IGF2-treated cultures after 2, 3 and 7 DIV under differentiation conditions (Fig. 1D,E), consistent with the increased levels of the protein observed by immunoblot in differentiation conditions after IGF2 treatment (Fig. 1F). To determine whether mitogen withdrawal could influence the increase in p57 levels in neurospheres cultures, we generated primary cultures from the adult SVZ that were maintained in the absence of mitogens (Costa et al., 2011; Ortega et al., 2011) and determined the levels of p57 in the presence or absence of IGF2 (Fig. S3D). Consistent with the data in neurosphere cultures, the addition of IGF2 to the primary cultures also induced the expression of p57 (Fig. S3D,E), which resulted in a more differentiated state of the cultures, as indicated by the increased percentage of GFAP^+^ astrocytes, βIII-tubulin^+^ neuroblasts and Olig2^+^ oligodendrocytes in IGF2-treated cultures (Fig. S3F,G).

### IGF2 regulates *Cdkn1c* expression during NSCs differentiation through Akt activation

*Cdkn1c* expression is finely regulated by several epigenetic mechanisms, including genomic imprinting. The mouse *Cdkn1c* gene belongs to the *Kcnq1ot1* imprinted cluster on mouse chromosome 7 and is canonically expressed from the maternally inherited chromosome (Fig. S4A) (Stampone et al., 2018). To explore whether the upregulation of *Cdkn1c* in IGF2-treated NSCs could be caused by a loss of imprinting of the *Cdkn1c* gene, we assayed the imprinting state of the gene in NSCs at 2 DIV under differentiation conditions. NSCs from wild-type adult F1 hybrid offspring from reciprocal crosses of *Mus musculus domesticus* (C57BL6/J) and *Mus musculus castaneus* (CAST/EiJ) strains, in which a single-nucleotide polymorphism (SNP) was identified at the *Cdkn1c* gene between the two subspecies, were analysed (Fig. S4B). IGF2-treated and untreated cultures showed the expected maternally inherited imprinted expression of *Cdkn1c*, and no expression of the paternal allele was observed (Fig. S4C), indicating that genomic imprinting was not altered after IGF2 treatment. The *Cdkn1c* gene has a somatic differentially methylated region (sDMR) that is located at the promoter of the gene that regulates its expression (Pateras et al., 2009; Stampone et al., 2018). Given that the imprinting state of the gene was not altered, and to further analyse whether DNA methylation of *Cdkn1c* promoter might be associated with the regulation of the expression of the gene after IGF2 treatment, we next determined the methylation levels of the sDMR by bisulphite sequencing. Consistent with the maintenance of genomic imprinting of *Cdkn1c*, IGF2-treated NSCs showed the expected levels of methylation (mean methylation percentage: untreated, 61.6±3.5%; IGF2 treated, 61.5±3.6%; *P*=0.96) (Fig. S4D), supporting a methylation-independent function of IGF2 on the regulation of expression of this gene in the adult NSCs.

**Fig. 2.**
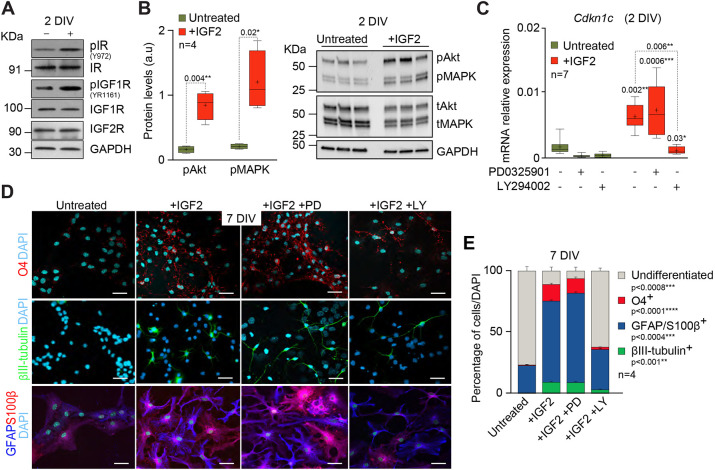
**IGF2 promotes *Cdkn1c* expression in NSCs through a mechanism involving PI3K-Akt.** (A) Immunoblot for phospho-IR (pIR), phospho-IGF1R (pIGF1R) and IGF2R in NSCs in differentiating conditions and after IGF2 treatment. GAPDH was used to normalize the quantity of protein. (B) Quantification of phosphorylated forms of AKT and MAPK protein levels by western blot (left). Immunoblot for phosphorylated AKT (pAkt) and MAPK (pMAPK), and total AKT (tAkt) and MAPK (tMAPK) in untreated and IGF2-treated cultures after 2 days in differentiation-promoting conditions (right). Data are presented relative to total Akt or MAPK protein. Boxes indicate interquartile range and whiskers indicate maximum and minimum values; *n*=4 experimental replicates (two-tailed paired Student's *t*-test). (C) qPCR for *Cdkn1c* in wild-type cultures in the absence or presence of IGF2 that have been additionally treated with the PI3K inhibitor LY294002 or the ERK1/2 inhibitor PD0325901 after 2 DIV under differentiation conditions. Data are mean±s.e.m.; boxes indicate interquartile range and whiskers indicate maximum and minimum values; *n*=7 experimental replicates (Mann–Whitney test). (D) Immunocytochemistry images for O4 (red), βIII-tubulin (green), GFAP (blue) and S100β (red) in wild-type NSCs after 7 DIV under differentiation conditions in the presence or absence of IGF2 that have been additionally treated with the PI3K inhibitor LY294002 (LY) or the MAPK inhibitor PD0325901 (PD). DAPI was used to counterstain DNA. (E) Percentage of cells that are positive for O4, GFAP/S100β and βIII-tubulin after 7 days in differentiation-promoting conditions in the presence or absence of IGF2 and that were additionally treated with LY or PD. Data are mean±s.e.m.; *n*=4 experimental replicates (repeated measures ANOVA with a post-hoc Tukey test). *P*-values and number of samples are indicated. Scale bars: 30 µm.

IGF2 binds with high affinity to the insulin receptor (IR) and Igf1 receptor (IGF1R), and is also able to interact with IGF2R to target the ligand to lysosomes for degradation (D'Ercole et al., 1996; Stewart and Rotwein, 1996). It has been previously shown that IGF2 activates phosphatidylinositol 3-kinase/protein kinase B (PI3K-Akt) and mitogen-activated protein kinase (MAPK) to promote cell cycle progression and differentiation (Bracko et al., 2012; Chirivella et al., 2017). More precisely, the link between IGF2 and p57 has been shown to be dependent on the activation of PI3K/Akt pathway in HSCs (Thomas et al., 2016). Therefore, to elucidate the intracellular pathways downstream of IGF2 in differentiating NSCs, a study at the mRNA level was performed in NSCs after 2 DIV under differentiation conditions. This study showed that *Insr* and *Igf1r* were the most strongly expressed receptors and maintained their level of expression after IGF2 treatment (Fig. S5A). Moreover, treatment with IGF2 induced the phosphorylation of IR and IGF1R, whereas no effect was observed on IGF2R (Fig. 2A). Furthermore, the levels of phosphorylated Akt (pAkt) and ERK1/2 (pMAPK) were determined after IGF2 treatment in differentiation conditions. This study revealed a significant increase of both pAkt and pMAPK in IGF2-treated NSCs after 2 DIV under differentiation conditions (Fig. 2B). Notably, simultaneous treatment of adult NSC cultures with IGF2 and the PI3K pharmacological inhibitor LY294002 blocked the effects of IGF2 on *Cdkn1c* expression (Fig. 2C and Fig. S5B), whereas treatment with the ERK1/2 inhibitor PD0325901 did not modify the increased levels of *Cdkn1c* expression induced by IGF2 (Fig. 2C and Fig. S5B). These results demonstrated that IGF2 promoted *Cdkn1c* expression in NSCs through a mechanism involving PI3K-Akt but not the ERK1/2-MAPK pathway.

To determine whether differentiation effects of IGF2 via p57 are mediated by activation of the PI3K-Akt pathway, wild-type NSCs were differentiated for 7 DIV into astrocytes, oligodendrocytes and neurons in the presence of IGF2 and LY294002 or PD0325901 (Fig. 2D). As previously shown, IGF2 promoted an increase of the percentage of βIII-tubulin^+^ neurons, GFAP^+^/S100β^+^ astrocytes and O4^+^ oligodendrocytes cells (Fig. 2D,E). However, the addition of LY294002 to IGF2-treated cultures reverted the differentiation effects of the factor, resulting in cultures with a smaller proportion of neurons, astrocytes and oligodendrocytes (Fig. 2D,E). Accordingly, the proportion of undifferentiated cells was increased in the presence of LY294002 (Fig. 2D,E). Importantly, the addition of PD0325901 did not have any effect on NSCs differentiation (Fig. 2D,E). These data confirm that the PI3K-Akt pathway mediates regulation of *Cdkn1c* expression by IGF2*.*

### p57 mediates the differentiation effects of IGF2 in adult NSCs *in vitro*

To evaluate whether p57 could mediate the differentiation effects of IGF2 in adult SVZ NSCs, a murine genetic model was generated by crossing mice carrying *loxP* sites flanking exons 2 to 4 of *Cdkn1c* gene (*Cdkn1c^loxp/loxp^*) (Matsumoto et al., 2011) with mice expressing the Cre-recombinase under the control of the mouse *Gfap* promoter (*Gfap-cre^+/0^*) (Fig. S6A) (Garcia et al., 2004; Montalbán-Loro et al., 2019). Given that *Cdkn1c* is an imprinted gene expressed only from the maternal allele, crosses were carried out reciprocally to generate heterozygous mice with either the maternal (*Cdkn1c-Gfap^mat^*) or the paternal (*Cdkn1c-Gfap^pat^*) deleted allele (Fig. S6A). To first determine the specificity of the *Gfap-cre* recombination, we crossed *Gfap-cre* females with ROSA26R males and performed an X-gal histochemistry in the adult brain of the resulting mice (*Gfap-cre/LACZ*). This analysis showed positive staining for β-galactosidase in the SVZ and RMS of the adult *Gfap-cre/LACZ* brains (Fig. S6B), corroborating the deletion of *Cdkn1c* in the adult GFAP^+^ stem cell population.

**Fig. 3.**
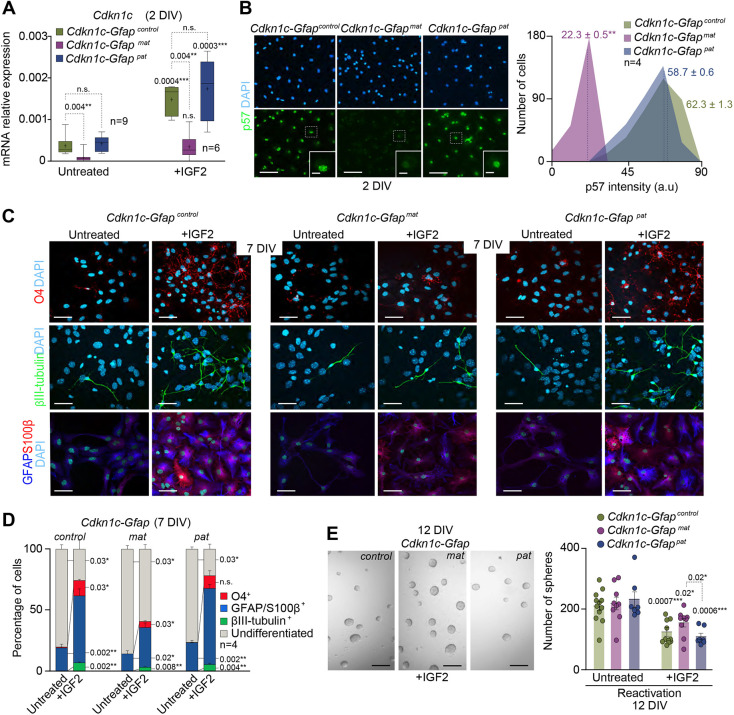
**p57 mediates the effects of IGF2 on NSC differentiation *in vitro*.** (A) qPCR for the *Cdkn1c* gene in *Cdkn1c-Gfap^control^*, *Cdkn1c-Gfap^mat^* and *Cdkn1c-Gfap^pat^* NSCs after 2 DIV under differentiation conditions in the presence or absence of IGF2. *Gapdh* was used as a housekeeping gene. Boxes indicate interquartile range and whiskers indicate maximum and minimum values; *n*=9 experimental replicates in untreated conditions and *n*=6 in IGF2-treated conditions (Mann–Whitney test). (B) Immunocytochemistry images for p57 (green) in *Cdkn1c-Gfap^control^*, *Cdkn1c-Gfap^mat^* and *Cdkn1c-Gfap^pat^* NSCs after 2 DIV under differentiation conditions (left panel). Histogram showing p57 intensity (in arbitrary units, a.u.) in *Cdkn1c-Gfap^control^*, *Cdkn1c-Gfap^mat^* and *Cdkn1c-Gfap^pat^* cultures after 2 DIV under differentiation conditions. Mean intensities are indicated as dashed lines (right panel). Data are mean±s.e.m.; *n*=4 experimental replicates (Friedman test). (C) Immunocytochemistry images for O4 (red), βIII-tubulin (green), GFAP (blue) and S100β (red) in *Cdkn1c-Gfap^control^*, *Cdkn1c-Gfap^mat^* and *Cdkn1c-Gfap^pat^* NSCs after 7 DIV under differentiation conditions and in the presence or absence of IGF2. (D) Percentage of cells that were positive for O4, GFAP/S100β or βIII-tubulin in untreated and IGF2-treated cultures after 7 DIV under differentiation-promoting conditions. Data are mean±s.e.m.; *n*=4 experimental replicates (Mann–Whitney test). (E) Representative images of neurospheres formed by differentiated cultures in the presence or absence of IGF2 (left panel). Number of neurospheres formed from *Cdkn1c-Gfap^control^*, *Cdkn1c-Gfap^mat^* and *Cdkn1c-Gfap^pat^* cultures after detaching 7 DIV-differentiated NSCs in the presence or absence of IGF2 and replating in proliferation conditions (right panel). Data are mean±s.e.m.; *n*=12, 9, 7, 11, 9 and 7 experimental replicates, respectively (Mann–Whitney test). DAPI was used to counterstain DNA. *P*-values and number of samples are indicated. Scale bars: 30 µm in B,C; 10 µm in insets in B; 100 µm in E.

A significant reduction of *Cdkn1c* mRNA and p57 protein was observed in *Cdkn1c-Gfap^mat^* NSCs after 2 DIV under differentiation conditions (Fig. 3A,B), but no downregulation of p57 was detectable in *Cdkn1c-Gfap^pat^* heterozygous NSCs (Fig. 3A,B), coincident with the canonical maternal expression of the gene. No changes in the levels of expression of *Cdkn1a* and *Cdkn1b* were observed at the same time of differentiation in *Cdkn1c-Gfap^mat^* and *Cdkn1c-Gfap^pat^* cultures (Fig. S6C). Importantly, body and brain weights were not affected in *Cdkn1c-Gfap^mat^* and *Cdkn1c-Gfap^pat^* compared with controls (Fig. S6D). To investigate the differentiation effects of IGF2 in the presence or in the absence of p57, NSCs from the adult SVZ of heterozygous *Cdkn1c-Gfap^mat^* and *Cdkn1c-Gfap^pat^* and control mice were expanded and differentiated for 7 DIV in the presence or absence of IGF2. We observed that IGF2 promoted the terminal differentiation of NSCs, which increases the percentage of βIII-tubulin^+^ neurons, GFAP^+^/S100β^+^ astrocytes and O4^+^ oligodendrocytes in control cultures, as previously shown (Fig. 3C,D). These effects were also observed in *Cdkn1c-Gfap^pat^* cultures; however, the proportions of these three neural lineages were not equally induced in *Cdkn1c-Gfap^mat^* cultures (Fig. 3C,D). Accordingly, the proportion of cells in the differentiated cultures with the capacity to activate and form new neurospheres in non-adherent cultures was reduced after IGF2 treatment in all cultures; however, *Cdkn1c-Gfap^mat^* cultures were not equally reduced compared with control or *Cdkn1c-Gfap^pat^* cultures (Fig. 3E). All these data confirm that maternal expression of *Cdkn1c* partly mediates the differentiation effects of IGF2 in adult NSCs *in vitro.*

### Maternal deficiency of *Cdkn1c* causes astrocytic differentiation of adult NSCs *in vivo*

**Fig. 4.**
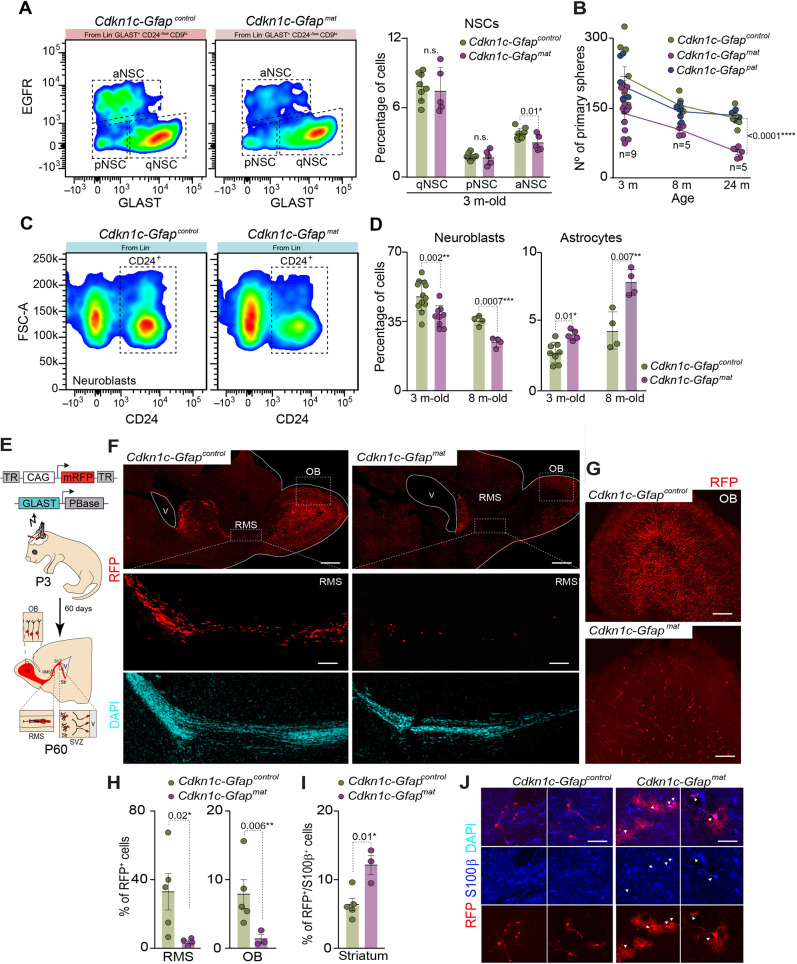
***Cdkn1c* deficiency causes terminal differentiation of activated NSCs into astrocytes *in vivo*.** (A) Flow cytometry analysis of the combined markers GLAST, EGFR and CD9 in *Cdkn1c-Gfap^control^* and *Cdkn1c-Gfap^mat^* SVZ. Different cell fractions are shown: cells that express low levels of GLAST with high levels of EGFR correspond to activated NSCs (aNSCs); cells that also express low levels of GLAST but with low levels of EGFR correspond to primed NSCs (pNSCs); GLAST^+^ cells that show low levels of EGFR correspond to quiescent NSCs (qNSCs) (left panel). Quantification of the percentage of qNSCs, pNSCs and aNSCs obtained by flow cytometry in dissociated cells from 3-month-old *Cdkn1c-Gfap^control^* and *Cdkn1c-Gfap^mat^* mice (right panel). Data are mean±s.e.m.; *n*=8, 5, 8, 5, 8 and 5 experimental replicates, respectively (two-tailed unpaired Student's *t*-test). (B) Number of primary spheres obtained from the SVZ of *Cdkn1c-Gfap^control^*, *Cdkn1c-Gfap^mat^* and *Cdkn1c-Gfap^pat^* in 3-, 8- and 12-month-old mice. Data are mean±s.e.m; *n*=9, 5 and 5 experimental replicates, respectively (linear regression test). (C) Flow cytometry analysis of CD24^+^ cells showing the neuroblast population in *Cdkn1c-Gfap^control^* and *Cdkn1c-Gfap^mat^* mice. (D) Percentage of neuroblasts and astrocytes analysed by flow cytometry in dissociated cells from the SVZ of 3- and 8-month-old *Cdkn1c-Gfap^control^* and *Cdkn1c-Gfap^mat^* mice. Data are mean±s.e.m.; *n*=12, 9, 4 and 4 experimental replicates, respectively, for neuroblasts; *n*=8, 5, 4 and 4 experimental replicates, respectively, for astrocytes (two-tailed unpaired Student's *t*-test). (E) Schematics of the electroporation strategy for labelling SVZ NSCs progeny. (F) Confocal images for RFP^+^ cells (red) in the brain of *Cdkn1c-Gfap^control^* and *Cdkn1c-Gfap^mat^* mice (upper panel). High-magnification images of the RFP^+^ cells migrating through the rostral migratory stream (RMS) in *Cdkn1c-Gfap^control^* and *Cdkn1c-Gfap^mat^* mice are shown (lower panel). (G) Confocal images for RFP^+^ cells (red) in the olfactory bulb (OB) of *Cdkn1c-Gfap^control^* and *Cdkn1c-Gfap^mat^* mice. (H) Percentage of RFP^+^ cells in the RMS and OB of *Cdkn1c-Gfap^control^* and *Cdkn1c-Gfap^mat^* mice. Data are mean±s.e.m.; *n*=5 and 4 experimental replicates for the RMS and OB, respectively (two-tailed unpaired Student's *t*-test). (I) Percentage of RFP^+^ cells that expressed S100β in the striatum of *Cdkn1c-Gfap^control^* and *Cdkn1c-Gfap^mat^* mice. Data are mean±s.e.m.; *n*=5 and 4 experimental replicates, respectively (two-tailed unpaired Student's *t*-test). (J) Immunohistochemistry confocal images for S100β and RFP cells in the striatal parenchyma of *Cdkn1c-Gfap^control^* and *Cdkn1c-Gfap^mat^* mice. DAPI was used to counterstain DNA. V, ventricle lumen. *P*-values and number of samples are indicated. Scale bars: 1 mm in F; 100 µm for higher magnification images in F; 100 µm in G; 30 µm in J.

Immunohistochemical analysis with antibodies to p57 and to cell-identity antigens in wild-type adult brains revealed nuclear staining for p57 protein in the GFAP population located close to the lateral ventricles (Fig. S7A), in mature neurons in the striatal parenchyma and in DCX^+^ neuroblasts reaching the olfactory bulb (Fig. S7A,B). Moreover, a transcriptomic analysis of the different populations in the neurogenic lineage from the adult SVZ and studied by flow cytometry (Belenguer et al., 2021a) revealed that GLAST^+^ quiescent NSCs (qNSCs) expressed very low levels of *Cdkn1c*, whereas GLAST^+^ primed NSCs (pNSCs) upregulated the levels of expression of the gene, being even higher in GLAST/EGFR^+^ activated NSCs (aNSCs) (Fig. S7C). Consistent with the *in vivo* immunostaining analysis, significant levels of expression of *Cdkn1c* were maintained in more differentiated cells, such as GLAST−/CD24+/PSA-NCAM+ neuroblasts and GLAST−/CD24−/EGFR+ neural progenitors (NPCs) (Fig. S7C). Thus, in order to characterize the function of p57 in the maintenance and differentiation of NSCs in the adult SVZ *in vivo*, a similar flow cytometry analysis of the different cell fractions from the SVZ was also performed in 3-month-old *Cdkn1c-Gfap^mat^* and control mice (Belenguer et al., 2021a,b). *Cdkn1c* deletion resulted in reduced percentage of aNSCs (Fig. 4A), correlating with a reduced number of primary neurospheres obtained from the SVZ of *Cdkn1c-Gfap^mat^* mice (Fig. 4B). Consequently, a decrease in the percentage of the neuroblast population was observed in *Cdkn1c*-deficient mice (Fig. 4C,D). Loss of p57 also resulted in a significant increase in the terminally differentiated astrocytic population in *Cdkn1c-Gfap^mat^* mice compared with wild types (Fig. 4D and Fig. S7D). Importantly, a similar analysis in 8-month-old mice confirmed the loss of NB production and the induction of astrocyte differentiation in *Cdkn1c-Gfap^mat^* mice, which were accelerated with age (Fig. 4D). This correlated with the exhaustion of neurosphere-forming cells isolated from the SVZ of 8- and 24-month-old *Cdkn1c-Gfap^mat^* mice (Fig. 4B). No changes in the EGFR^+^ transit amplifying progenitor population or in the O4^+^ oligodendrocyte progeny was observed in *Cdkn1c*-deficient mice (Fig. S7E-H).

To further study the role of p57 in NSC differentiation *in vivo*, we performed brain electroporation in *Cdkn1c-Gfap^control^* and *Cdkn1c-Gfap^mat^* newborn pups at 3 days of age (Fig. 4E). This procedure takes advantage of the brain ventricles to allow the introduction of DNA into the lining NSCs (Fig. 4E). We used a Piggy BAC integrative vector carrying a red fluorescent protein (RFP) together with a GLAST-Transposase to permanently label the NSCs and their progeny with RFP (Fig. 4E). Sixty days after electroporation, we found a considerable number of RFP-labelled cells migrating through the RMS, and reaching and integrating with the OB in control mice (Fig. 4F-H). RFP^+^ cells were far less numerous in the RMS of *Cdkn1c-Gfap^mat^* mice, resulting in fewer NBs in the OB (Fig. 4F-H). Notably, we found terminally differentiated astrocytes in the striatum of *Cdkn1c-Gfap^mat^* mice, as indicated by the increased percentage of RFP^+^ cells that expressed the astrocytic marker S100β (Fig. 4I,J). These data confirmed a role for p57 in the differentiation process of adult NSCs also *in vivo*.

### Intraventricular infusion of IGF2 promotes NSCs differentiation *in vivo* through activation of *Cdkn1c*

**Fig. 5.**
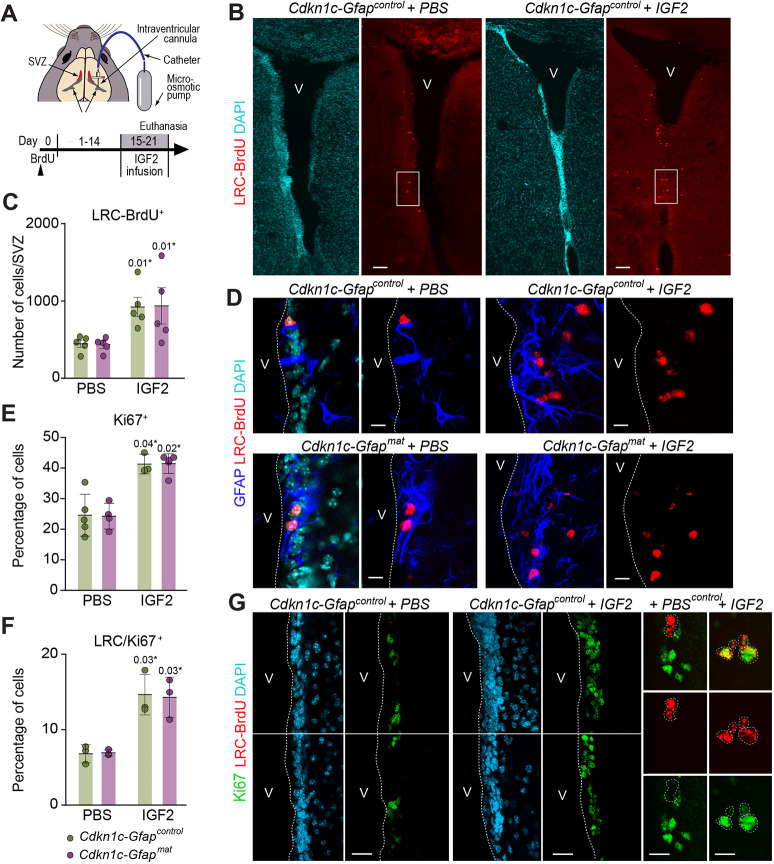
**IGF2 promotes NSC proliferation *in vivo* independently of p57.** (A) Schematic representation of the ventricular infusion of IGF2 and BrdU injection regime. (B) Immunohistochemistry confocal images for BrdU-LRC (red) in the SVZ of *Cdkn1c -Gfap^control^* mice after intraventricular infusion of PBS or IGF2. Each image comprises two tiled confocal fields of view. Area outlined is shown in D. (C) Total number of BrdU-LRC in the SVZ of *Cdkn1c-Gfap^control^* and *Cdkn1c -Gfap^mat^* mice after the infusion of PBS or IGF2. Data are mean±s.e.m.; *n*=5 experimental replicates (two-way ANOVA with a post-hoc Tukey test). (D) Immunohistochemistry for BrdU-LRC (red) and GFAP (blue) in the SVZ of *Cdkn1c -Gfap^control^* and *Cdkn1c -Gfap^mat^* mice after the infusion of PBS or IGF2. (E) Percentage of Ki67^+^ cells in the SVZ of *Cdkn1c-Gfap^control^* and *Cdkn1c -Gfap^mat^* mice after the infusion of PBS or IGF2. Data are mean±s.e.m.; *n*=5, 4, 3 and 5 experimental replicates (Mann–Whitney test). (F) Percentage of BrdU-LRC/Ki67^+^ cells in the SVZ of *Cdkn1c-Gfap^control^* and *Cdkn1c -Gfap^mat^* mice after the infusion of PBS or IGF2. Data are mean±s.e.m; *n*=4 experimental replicates (Mann–Whitney test). (G) Immunohistochemistry confocal images for Ki67 (green) and BrdU-LRC (red) in the SVZ of *Cdkn1c -Gfap^control^* mice after intraventricular infusion of PBS or IGF2. DAPI was used to counterstain DNA. V, ventricle lumen. *P*-values and number of samples are indicated. Scale bars: 100 µm in B; 30 µm in D and G; 7 µm for higher magnification images in G.

Finally, in order to functionally test whether p57 could also mediate the differentiation effects of IGF2 *in vivo*, we infused PBS solution or 100 µg ml^−1^ of recombinant IGF2 for 7 days into the lateral ventricle (Ferrón et al., 2007; Mellott et al., 2014) of *Cdkn1c-Gfap^mat^* and *Cdkn1c-Gfap^control^* mice brains (Fig. 5A). We first injected mice with BrdU 15 days before pump implantation and euthanized them immediately after pump removal (Fig. 5A). In the SVZ, fast-proliferating transit-amplifying progenitors dilute out the BrdU, which is specifically retained in slowly proliferating NSCs (label-retaining cells, BrdU-LRCs), in newborn neurons and in newborn oligodendrocytes that cease to divide and undergo terminal differentiation soon after the injection in the OB or in the CC, respectively (Ferrón et al., 2007; Menn et al., 2006). The number of BrdU-LRC^+^ cells was significantly increased after IGF2 infusion in both wild-type and *Cdkn1c*-deficient mice (Fig. 5B-D), and more of them were positive for the proliferation antigen Ki67 (Fig. 5E-G), supporting a role for IGF2 in regulating the number of activated NSCs within the SVZ, although this effect was independent of p57.

**Fig. 6.**
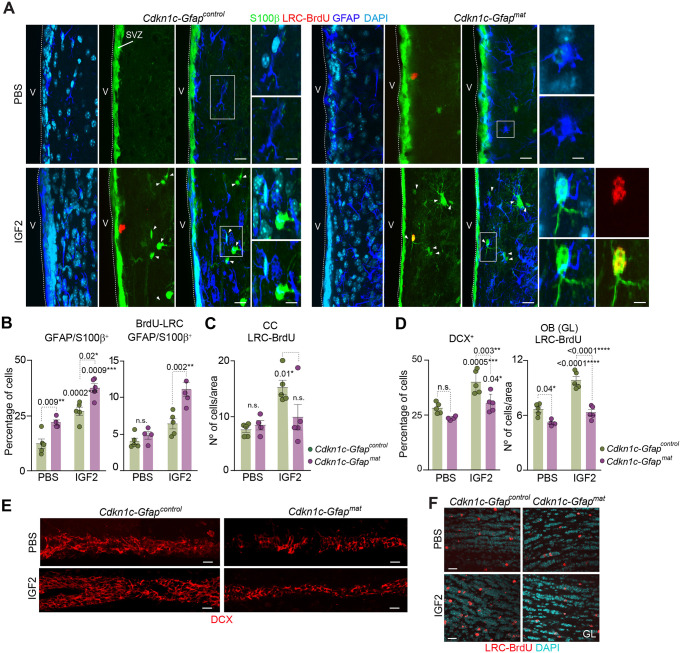
**IGF2 infusion into the lateral ventricles induces neurogenesis and oligodedrogenesis via p57.** (A) Immunohistochemistry confocal images for S100β (green), GFAP (blue) and BrdU-LRC (red) in the SVZ of *Cdkn1c-Gfap^control^* and *Cdkn1c-Gfap^mat^* mice after the infusion of PBS or IGF2. Arrowheads indicate positive cells. V, ventricle lumen. Areas outlined with rectangles are shown at higher magnification on the right. (B) Percentage of double GFAP/S100β^+^ cells in the SVZ of *Cdkn1c-Gfap^control^* and *Cdkn1c-Gfap^mat^* mice after the infusion of PBS or IGF2 (left panel). Percentage of GFAP/S100β^+^ cells that are also LRC-BrdU in the SVZ of *Cdkn1c-Gfap^control^* and *Cdkn1c-Gfap^mat^* mice after the infusion of PBS or IGF2 (right panel). Data are mean±s.e.m.; *n*=5 experimental replicates (two-way ANOVA with a post-hoc Tukey test). (C) Quantification of the number of newborn oligodendrocytes that become incorporated into the corpus callosum in the same conditions. Data are mean±s.e.m.; *n*=5 experimental replicates (two-way ANOVA with a post-hoc Tukey test). (D) Percentage of DCX^+^ cells in the SVZ of *Cdkn1c-Gfap^control^* and *Cdkn1c-Gfap^mat^* mice after the infusion of PBS or IGF2 (left panel). Quantification of the number of newborn neurons incorporating in the granular layer (GL) in the OB of *Cdkn1c-Gfap^control^* and *Cdkn1c-Gfap^mat^* mice in the same conditions (right panel). Data are mean±s.e.m.; *n*=5 experimental replicates (two-way ANOVA with a post-hoc Tukey test). (E) Immunohistochemistry confocal images for chains of DCX^+^ (red) neuroblasts in the SVZ of *Cdkn1c-Gfap^control^* and *Cdkn1c-Gfap^mat^* mice after intraventricular infusion of PBS or IGF2. (F) Immunohistochemistry confocal images for BrdU-LRC (red) in the OB of *Cdkn1c-Gfap^control^* and *Cdkn1c-Gfap^mat^* mice after intraventricular infusion of PBS or IGF2. DAPI was used to counterstain DNA. *P*-values and number of samples are indicated. Scale bars: 30 µm in A, E and F; 7 µm in higher magnification images in A.

Immunohistological analysis of cell populations within the SVZ of *Cdkn1c-Gfap^control^* and *Cdkn1c-Gfap^mat^* mice confirmed the increased astrocytic differentiation of cells in p57-deficient mice, as indicated by the higher proportion of GFAP^+^ cells that were also positive for S100β found in the SVZ of *Cdkn1c-Gfap^mat^* mice compared with control mice (Fig. 6A,B). Notably, IGF2 infusion in the lateral ventricle of *Cdkn1c-Gfap^control^* mice resulted in a higher proportion of GFAP/S100β^+^ cells and this increase was even higher in *Cdkn1c-Gfap^mat^* (Fig. 6A,B). Importantly, more of these GFAP/S100β^+^ cells retained BrdU in *Cdkn1c-Gfap^mat^* mice (Fig. 6A,B and Fig. S8A), suggesting an accumulation of inactive cells with mature astrocytic fate in the absence of p57.

Higher numbers of newly generated BrdU^+^ oligodendrocytes were also found in the CC of wild-type mice after IGF2 infusion (Fig. 6C). In addition, more densely populated DCX^+^ neuroblast chains in the RMS were found in the wild-type SVZ after IGF2 infusion (Fig. 6D,E; Fig. S8B,C), which resulted in higher numbers of newly generated BrdU^+^ neurons in the granular and periglomerular layers (PGL) of the OB (Fig. 6D,F and Fig. S8D). However, no effects of IGF2 infusion were observed in the number of BrdU^+^ newly generated neurons and oligodendrocytes in the absence of p57 (Fig. 6C-F and Fig. S8C,D), confirming that p57 is required for the differentiation effects of IGF2 also *in vivo*.

## DISCUSSION

**Fig. 7.**
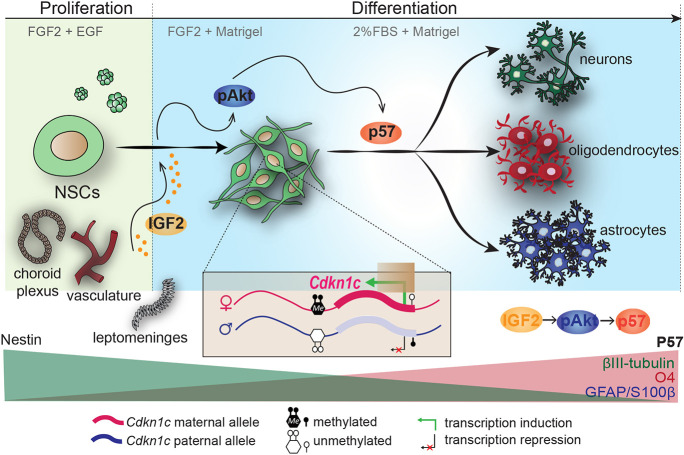
**IGF2 promotes adult NSC differentiation through upregulation of the maternal expression of *Cdkn1c* in a PI3K-Akt-dependent-manner.** Schematic drawing the process of adult NSCs differentiation. IGF2 is secreted in the SVZ neurogenic niche by the vasculature, meninges, leptomeninges and choroid plexus, promoting the phosphorylation of Akt. The activation of the PI3K-Akt pathway induces the expression of the maternal allele of the imprinted gene *Cdkn1c*, which finally induces NSC terminal differentiation into neurons, oligodendrocytes and astrocytes.

This study shows that IGF2 and *Cdkn1c* interact to regulate the differentiation potential of adult NSCs. Treatment of NSCs with recombinant IGF2 promotes a more differentiated phenotype of NSCs and a reduction of their stemness *in vitro*. The promotion of terminal differentiation of NSCs correlates with the induction of an increased level of the maternally expressed gene *Cdkn1c* through activation of the PI3K-Akt pathway. Consistently, intraventricular infusion of IGF2 in wild-type results in a final stimulation of NSCs differentiation. Importantly, infusion of IGF2 in *Cdkn1c*-deficient mice also induces proliferation; however, the absence of p57 in infused mice results in terminal differentiation of non-multipotent astrocytes at the expense of neurons and oligodendrocytes (Fig. 7). Our data present a model that illustrates the requirement of the correct interaction of IGF2 and the imprinted gene *Cdkn1c* to ensure long-term neurogenesis in the adult SVZ neurogenic niche.

Genomic imprinting controls the allelic expression of a subset of dose-sensitive genes in a parent of origin-dependent manner. Imprinted genes can be classified into canonical imprinted genes and genes that switch detectable expression of both parental alleles in different tissues and/or during development (Laukoter et al., 2020). Genes belonging to the second category include *Igf2*, which, although paternally expressed by most tissues, has also been shown to be biallelically expressed by the CP and vasculature in the SVZ niche and has a critical function in postnatal neurogenesis (Ferrón et al., 2015). In contrast, NSCs within the SGZ rely on canonically imprinted autocrine IGF2 expressed from only the paternal allele to regulate hippocampal stem cell function (Ferrón et al., 2015). Along the same lines, we have previously shown that loss of genomic imprinting of the paternally expressed gene *Dlk1* is necessary to maintain NSC function in both neurogenic niches: the SVZ and the SGZ (Ferrón et al., 2011; Montalbán-Loro et al., 2021). Notably, many of these imprinted genes work coordinately and even antagonistically to control foetal growth (Daniel et al., 2015; Hoffmann et al., 2014). Thus, dynamic changes in genomic imprinting translate into biologically relevant functions.

The CKI p57 has been described to induce cell cycle exit, differentiation, neurite outgrowth and migration during embryonic cortical development (Imaizumi et al., 2020; Laukoter et al., 2020; Mairet-Coello et al., 2012), and is known to control NSC quiescence in hippocampal neurogenesis (Furutachi et al., 2013). More concretely, p57 has been shown to play a non-canonical role in regulating the activity of transcription factors implicated in neuronal differentiation interacting robustly with a subset of neurogenic bHLH proteins, including *Mash1* (*Ascl1*), *Neurod1* and *Nex/Math2* (*Neurod6*), that inhibit neuronal differentiation, independently of the interaction with CDKs and cell-cycle exit (Joseph et al., 2009). Moreover, loss- and gain-of-function approaches suggest that p57 exerts a context-dependent repressive effect on neuronal but not astrocyte differentiation after mitogen withdrawal to allow proper glial differentiation (Joseph et al., 2009). In the SGZ of the hippocampus it has been shown that p57 is expressed at high levels in radial NSCs and that this CKI is required for NSC quiescence. Consistently, p57 diminishes in proliferating active radial NSCs (Furutachi et al., 2013). However, our immunohistochemical analysis in the SVZ shows that p57 is present in GFAP^+^ cells, whereas it is absent in neuroblasts within the SVZ. Interestingly, our data also show that p57 increases during neuronal and astroglial differentiation *in vitro* and becomes highly expressed in mature astrocytes and in more differentiated neuroblasts that reach the OB *in vivo.*

Given the absence of decisive markers to identify the neural stem cell population *in vivo*, part of the analysis of adult NSCs has relied on a neurospheres *ex vivo* assay obtained from the SVZ neurogenic niche (Bizy and Ferrón, 2014; Ferrón et al., 2007; Reynolds and Weiss, 1992). Moreover, recent FACS studies using different combinations of markers have led to the identification of quiescent and activated NSCs (Basak et al., 2018; Belenguer et al., 2021b; Daynac et al., 2016; Llorens-Bobadilla et al., 2015; Morizur et al., 2018). This strategy, in combination with our primary neurospheres assay in wild-type and p57 deficient cultures, has shown an impairment of self-renewal potential and a consistent lower number of activated NSCs *in vivo* after p57 deficiency*.*

In addition, *in vitro* treatment of NSCs with IGF2 promotes a more differentiated phenotype and a reduction of their sternness capacity. Notably, these IGF2-mediated effects occur along with the induction of an increased level of the maternally expressed gene *Cdkn1c*, which is not due to an alteration of the imprinting status of the gene but is regulated through activation of the PI3K-Akt pathway. As a result, an IGF2-mediated increase in the levels of p57 *in vitro* promotes neuronal and glial differentiation of NSCs. This was corroborated with the intraventricular infusion of IGF2 in wild-type mice, which stimulates the differentiation of NSCs within the adult SVZ. These results were consistent with previous studies in a murine model of Alzheimer's disease, in which IGF2 infusion increased the population of DCX^+^ cells in the adult hippocampus through stimulation of NGF, BDNF, NT3 and IGF1 (Mellott et al., 2014). Importantly, our study also revealed that, after infusion of IGF2 in *Cdkn1c* deficient mice, NSCs accumulate and differentiate more markedly into non-multipotent committed astrocytes, limiting neurogenesis and oligodendrogenesis. Notably, IGF2 infusion in wild-type mice also induces the proliferation of NSC within the neurogenic niche, effects that are also observed after the infusion of other growth factors, such as EGF, FGF, BDNF or GDNF (Kobayashi et al., 2006; Türeyen et al., 2005; Zigova et al., 1998). However, infusion of IGF2 in *Cdkn1c*-deficient mice resulted in similar rates of proliferation than in wild-type mice, indicating that effects of IGF2 on NSCs activation are not mediated by p57, consistent with its role as a cell cycle inhibitor. Therefore, our data present a model that illustrates the requirement of the correct interaction of the two imprinted genes *Igf2* and *Cdkn1c* to balance neurogenesis, oligodendrogenesis and astrogliogenesis in the adult SVZ neurogenic niche (Fig. 7).

The human imprinted gene *CDKN1C* is the most frequently silenced or mutated gene in the imprinting genetic disorder Beckwith-Wiedemann syndrome (BWS); aberrant imprinting of *IGF2* has also been shown to interact with *CDK1C* in this imprinting disorder (Caspary et al., 1999; Grandjean et al., 2000). Interestingly, both genes are located in the same imprinted gene cluster on human chromosome II (Grandjean et al., 2000). Moreover, alterations in both imprinted genes can lead to malignant cell transformation in different cancers (Bastaki et al., 2016; Nakashima et al., 2015). Therefore, our study highlights the importance of probing the relative contributions of imprinted genes and their inhibition to the regulation of cell signalling during adult neurogenesis in non-pathological conditions. In future studies, it will be important to investigate the precise roles of these genes in neurodevelopmental disease conditions.

## MATERIALS AND METHODS

### Animals and *in vivo* manipulations

All transgenic mice used in the study were maintained in the C57BL6 background. Gfap-cre [6.Cg-Tg(Gfap-cre)73.12Mvs/J] mice were obtained from the Jackson Laboratory and genotyped as described previously (Garcia et al., 2004). *Gfap-Cre* mice were generated using a 15 kb mouse *Gfap* promoter cassette containing all introns, promoter regulatory elements, exons, and 2 kb of 3′ and 2.5 kb of 5′ flanking regions of the mouse *Gfap* gene (Johnson et al., 1995). To avoid the expression of exogenous *Gfap*, a small fragment of the first exon of the *Gfap* gene was removed (Johnson et al., 1995). *Cdkn1c^loxp/loxp^* mice were obtained from Riken BioResource Center (Japan) and contain LoxP sites flanking exons 2 to 4 (which include the entire coding region) of *Cdkn1c* gene (Furutachi et al., 2013; Matsumoto et al., 2011). To specifically ablate the maternal *Cdkn1c* allele in GFAP^+^ cells, we crossed female *Cdkn1c^loxp/loxp^* mice with a male harbouring a *Cre* transgene under the control of the *Gfap* promoter (*Gfap-Cre*). Reciprocally, to ablate the paternal allele, female *Gfap-Cre* mice were crossed with male *Cdkn1c^loxp/loxp^* mice (see Fig. S6A). *Gfap-Cre* mice were also crossed with a reporter mouse, which carry the β-galactosidase gene under the regulation of the ubiquitous ROSA26 promoter containing a loxP-flanked stop sequence (*Gfap-Cre/LACZ*). Control mice were generated by crossing mice without Cre-recombinase with ROSA26R mice (*Gfap-control/LACZ*). Animals were genotyped by PCR analysis of DNA extracted from ear-punch tissue and amplified with the primers for the presence of Cre-recombinase Cre-F and Cre-R (Table S1), and for the presence of LoxP sites p57 CKO-F, p57 CKO-R and p57 3′-Rv (Table S1). For imprinting studies, hybrid F1 offspring from reciprocal crosses between the subspecies *Mus musculus domesticus* and *Mus musculus castaneus* were used. Mice were maintained in a 12 h light/dark cycle with free access to food and water and according to the Animal Care and Ethics committee of the University of Valencia.

### Surgical and histological procedures

BrdU administration regimes have been previously detailed (Ferrón et al., 2007, 2011). Briefly, mice at 2-4 months of age were injected intraperitoneally with 50 mg of BrdU per kg of body weight every 2 h for 12 consecutive hours (seven injections in total). Two weeks after injections, animals were deeply anaesthetized with a mixture of ketamine and medetomidine, and fixed them to a stereotactic device (NeuroLab). We infused IGF2 (100 µg ml^−1^; R&D Sytems) in PBS or PBS alone into the lateral ventricle for 7 days by means of an osmotic mini-pump (Alzet, model 1007D; flow rate 0.5 µl/h) coupled to a cannula for intracerebral delivery (see Fig. 5A). The stereotactic coordinates for targeting the lateral ventricle were anteroposterior −0.1 mm, mediolateral −0.8 mm from bregma and dorsoventral −3.0 mm from the skull surface. Seven days later, animals were transcardially perfused with 4% paraformaldehyde (PFA) in 0.1 M phosphate buffer saline (PBS) pH 7.4 and brains were vibratome sectioned at 40 µm. For *in vivo* postnatal electroporation, a RFP Piggy BAC plasmid was injected together with a GLAST-Transposase plasmid in mouse pups at postnatal day 2 (P2) using a square wave electroporator (ECM 830 Square Wave Electroporation System, BTX) (Fabra-Beser et al., 2021; Mateos-White et al., 2020). Pups were deeply anesthetized by isoflurane inhalation and once the pedal reflex was lost, ≈1 μl of a 2 µg µl^−1^ plasmid solution was injected into one of the lateral ventricles (Sonego et al., 2013). Five pulses of 95 ms at 95 V and spaced 950 ms were delivered, using forcep electrodes (Platinum Tweezertrode 5 mm Diameter, BTX) at 45° to target the subpalial SVZ (Mateos-White et al., 2020). Pups were reanimated on a heating pad and returned to mother's cage until culling day. Perfusion and tissue processing was carried out at P60 as previously described. To normalize the electroporation efficiency between animals, the percentage of RFP^+^ cells was calculated relative to RFP^+^ cells in the SVZ. All counting were carried out blinded to avoid the risk of bias.

### Immunohistochemistry and β-galactosidase staining

For immunohistochemistry, sections were washed in PBS and blocked at room temperature for 1 h in PBS with 0.1% Triton X-100 supplemented with 10% Foetal bovine serum (FBS) and then incubated overnight at 4°C with primary antibodies (see Table S2). For BrdU detection, sections were pre-incubated in 2 N HCl for 20 min at 37°C and neutralized in 0.1 M sodium borate (pH 8.5) for 10 min. Detections were performed with fluorescent secondary antibodies incubated for 1 h at room temperature (see Table S3). For SVZ wholemounts, the lateral walls of the lateral ventricles were dissected free and the resulting wholemounts were fixed for 1.5 h in 4% PFA and washed overnight at 4°C in PBS. Wholemounts were washed three times in PBS containing 0.5% Triton X-100 for 15 min each, blocked for 2 h in 10% FBS and 2% Triton X-100 in PBS, then incubated for 48 h at 4°C with primary antibodies in the same blocking solution. After incubation with appropriate secondary antibodies, the stained walls were mounted with Fluorsave (Calbiochem). Nuclei were counterstained with 1 µg ml^−1^ of DAPI. Primary and secondary antibodies and dilutions used are listed in Tables S2 and S3, respectively. Samples were captured and analysed with an Olympus FV10i confocal microscope (Olympus). For β-galactosidase staining, brain samples were fixed with 4% PFA in 0.1 M PBS (pH 7.4), 2 mM MgSO_4_ and 5 mM EGTA for 30 min at 4°C and processed for vibratome sectioning. Sections were incubated in PBS with 2 mM MgCl_2_, 5 mM K_3_Fe(CN)_6_, 5 mM K_4_Fe(CN)_6_, 0.01% sodium deoxycholate and 0.02% NP-40, and 1 mg ml^−1^ X-Gal for 24 h at 37°C and washed several times in PBS.

### Neurosphere cultures, differentiation assays and immunofluorescence

Adult mice were sacrificed by cervical dislocation. To initiate each independent culture, the brains were dissected free and the regions containing the SVZ were isolated from each hemisphere and washed in Earle's balanced salt solution (EBSS; Gibco). Tissues were transferred to EBSS containing 1.0 mg ml^−1^ papain (Worthington DBA), 0.2 mg ml^−1^ L-cystein (Sigma), 0.2 mg ml^−1^ EDTA (Sigma) and incubated for 30 min at 37°C. Tissue was then rinsed in EBSS, transferred to Dulbecco's modified Eagle's medium (DMEM)/F12 medium (1:1 v/v; Life Technologies) and carefully triturated with a fire-polished Pasteur pipette to a single cell suspension. Isolated cells were collected by centrifugation, resuspended in DMEM/F12 medium containing 2 mM L-glutamine, 0.6% glucose, 9.6 g ml^−1^ putrescine, 6.3 ng ml^−1^, progesterone, 5.2 ng ml^−1^ sodium selenite, 0.025 mg ml^−1^ insulin, 0.1 mg ml^−1^ transferrin, 2 µg ml^−1^ heparin (sodium salt, grade II; Sigma) and supplemented with 20 ng ml^−1^ epidermal growth factor (EGF; Invitrogen) and 10 ng ml^−1^ fibroblast growth factor (FGF; Sigma) (growth medium) (Belenguer et al., 2016; Ferrón et al., 2007). Neurospheres were allowed to develop for 6 days in a 95% air/5% CO_2_ humidified atmosphere at 37°C. To estimate proliferation by the MTS assay, 15,000 cell/cm^2^ were plated after Accutase disaggregation in differentiation medium for 2 DIV and the absorbance at 490 nm was measured using the CellTiter 96 Aqueous One Solution Cell Proliferation Assay following the manufacturer's protocol and the spectrophotometer Victor 3-1420 (PerkinElmer). For culture expansion, cells were plated at a relatively high density (10 cell/μl) and maintained for several passages. For bulk differentiation assays, 80,000 cell/cm^2^ were seeded in Matrigel-coated coverslips and incubated 2 days in neurosphere culture medium without EGF. Medium was then changed with fresh medium without FGF but supplemented with 2% FBS for 5 more days (see Fig. 1A). For reactivation assays, 7 DIV differentiated NSCs were detached and replated in proliferation-promoting conditions (with mitogens) for 5 more days before counting the number of neurospheres formed. For primary cultures in the absence of mitogens, a previously described protocol was used (Ortega et al., 2011). Briefly, SVZ tissue was enzymatically digested with 0.7 mg ml^−1^ hyaluronic acid (Sigma) and 1.33 mg ml^−1^ trypsin (Sigma) in Hanks' Balanced Salt Solution (HBSS; Invitrogen) with 2 mM glucose for 30 min at 37°C. Tissue was rinsed in ice-cold medium composed of EBSS, 4% BSA (Sigma) and 20 mM HEPES. Tissue was then carefully triturated with a fire-polished Pasteur pipette to a single cell suspension. Cells were collected by centrifugation and washed first in ice-cold medium containing of 0.9 M sucrose (Sigma) and 0.5×HBSS, and second in ice-cold medium with 4% BSA and 2 mM HEPES. The final pellet was resuspended in culture medium containing DEMEM/F12 Glutamax (Invitrogen) supplemented with B27 (Invitrogen), 2 mM glutamine (Sigma) and 8 mM HEPES. Cells were plated on pretreated p24 wells with Poly-D-lysine (Sigma) at a density of 200-300 cells per mm^2^ and cultured for 5 days. When indicated, cultures were treated with IGF2 (R&D Systems; 100 ng ml^−1^), the PI3 K/Akt inhibitor LY294002 (Sigma; 50 µM) or the MEK1/2 inhibitor III PD0325901 (Millipore; 1 µM) at the time of plating. IGF2 treatments were performed in insulin-free medium. Cultures were fixed for staining at specific times of differentiation with 4% PFA and 0.1 M PBS for 15 min, and performed immunocytochemistry as described previously (Belenguer et al., 2016). Primary and secondary antibodies and dilutions used are listed in Tables S2 and S3, respectively. DAPI (1 µg ml^−1^) was used to counterstain DNA. Laser settings were first established on wild-type tissue or untreated samples, and similar regions of interest were acquired in an Olympus FV10i confocal microscope. Maximal projection images were generated and the mean grey intensities of nuclear marker p57 were measured with ImageJ/Fiji software. Intensities are represented as frequency histograms normalized to the maximum count in each comparison.

### Flow cytometry analysis

Cell analysis by flow cytometry were done following previously established protocols (Belenguer et al., 2021b). The SVZ of both brain hemispheres from each mouse were minced and enzymatically digested using the Neural Tissue Dissociation kit (T) in a GentleMACS Octo Dissociator with heaters (Miltenyi). Digested pieces were mechanically dissociated by pipetting up and down 20 times through a plastic Pasteur pipette. Cell suspension was filtered through a 40 mm nylon filter. Cells were pelleted (300 ***g***, 10 min), resuspended in 100 ml blocking buffer (HBSS without calcium and magnesium, 10 mM HEPES, 2 mM EDTA, 0.1% glucose and 0.5% BSA) and incubated with different combinations of specific primary antibodies at 4°C for 30 min (see Table S2). Cells were also incubated with EGF-Alexa488 or EGF-Alexa647 (1:300; Molecular Probes) at 4°C for 30 min. After washing with 1 ml of blocking buffer, labelled samples were centrifuged (300 ***g*** for 10 min at 4°C) and resuspended in 0.5 ml of blocking buffer for analysis. All analyses were performed with a LSR-Fortessa cytometer (Becton Dickinson) with 350, 488, 561 and 640 nm lasers. Dead cells were excluded by staining with 0.1 mg ml^−1^ DAPI before analysis.

### Expression studies and SNP sequencing

RNAs were extracted with RNAeasy mini kit (Qiagen) including DNase treatment, following the manufacturer's guidelines. For quantitative PCR, 1 µg of total RNA was reverse transcribed using random primers and RevertAid H Minus First Strand cDNA Synthesis kit (Thermo Scientific), following standard procedures. Thermocycling was performed in a final volume of 10 µl containing 1 µl of cDNA sample (diluted 1:20), and the reverse transcribed RNA was amplified by PCR with appropriate Taqman probes (see Table S4). Quantitative PCR was used to measure gene expression levels normalized to *Gapdh*, the expression of which did not differ between the groups. qPCR reactions were performed in a Step One Plus cycler with Taqman Fast Advanced Master Mix (Applied Biosystems). To study specific expression of the paternal and maternal alleles, PCR was carried out for the *Cdkn1c* gene in NSCs derived from adult F1 mice hybrid offspring from *Mus musculus domesticus* (C57BL6/J) and *Mus musculus castaneus* (CAST/EiJ) mice. We have previously identified a single-nucleotide polymorphism (SNP) between the two subspecies at the *Cdkn1c* gene (Fig. S4B). The SNP was a ‘T’ nucleotide in BL6 and a ‘C’ nucleotide in CAST mice, and was located at position 1382 in the Cdkn1c transcript (*Cdkn1c* sequence NM_001354981.1). The PCR product was purified and sequenced by Eurofins Genomics and analysed using the Gene Align programme.

### DNA methylation and pyrosequencing

DNA methylation level was quantified using bisulfite conversion and pyrosequencing. The DNA was bisulfited-converted using EZ DNA Methylation-Gold kit (Zymo Research) in accordance with the manufacture's protocol. Specifically, for *Cdkn1c* promoter, bisulfite-converted DNA was amplified by PCR with specific primer pairs: *Cdkn1cMETH-F* and *Cdkn1cMETH-R* (Table S1). PCRs were carried out in a 20 µl volume, with 2 U HotStar Taq polymerase (Qiagen), PCR buffer 10× (Qiagen), 0.2 mM dNTPs and 400 mM primers. PCR conditions were: 96°C for 5 min, followed by 39 cycles of 94°C for 30 s, 54°C for 30 s and 72°C for 1 min. For pyrosequencing analysis, a biotin-labelled primer was used to purify the final PCR product using sepharose beads. The PCR product was bound to Streptavidin Sepharose High Performance (GE Healthcare), purified, washed with 70% ethanol, denatured with 0.2 N NaOH and washed again with 10 mM Tris-acetate. Pyrosequencing primer (*Cdkn1cMETH-seq*; 400 mM) was then annealed to the purified single-stranded PCR product and pyrosequencing was performed using the PyroMark Q96MD pyrosequencing system using PyroMark reactives (Qiagen).

### Immunoblotting

Cells were lysed in cold RIPA buffer. Total protein concentration was determined using the BCA system (Pierce). Equal amounts (30 mg) of protein were loaded on polyacrylamide gels for SDS–polyacrylamide gel electrophoresis. Proteins were transferred to polyvinylidene difluoride (PVDF) membranes and immunoblots were carried out using primary antibodies (Table S2) followed by incubation with appropriate secondary horseradish peroxidase-conjugated antibodies (Table S3) and chemoluminiscent detection (Western Lightning, PerkinElmer). All antibodies were diluted in PBS containing 5% semi-skimmed milk and 0.1% Tween-20. Proteins were revealed using Lightning Plus ECL (Perkin Elmer) and the bands were analysed by densitometry using ImageJ (NIH) software.

### Statistical analysis

All statistical tests were performed using the GraphPad Prism Software, version 7.00 for Windows. Data were first tested for normality with a Shapiro-Wilk test. The significance of the differences between groups were evaluated with adequate statistical tests for each comparison. For data that passed normality tests: when analysing only one variable, a paired *t*-test was used for comparing two groups and one-way ANOVA followed by Tukey's post-hoc test for three or more groups. When two variables were analyse, two-way ANOVA followed by Tukey post-hoc test was used. For data groups that did not pass normality, Wilcoxon or Mann–Whitney non-parametric tests were performed, depending on whether samples were paired or not, respectively. For variables with more than two categories, Kruskal–Wallis or Friedman tests (for unpaired or paired data, respectively) were used followed by a Benjamini, Krieger and Yekutieli post-hoc test. For temporal measures that passed the normality test, lineal regression analysis was performed. When comparisons were performed with relative values (percentages), data were previously normalized by using arcsin root transformation. Values of *P*<0.05 were considered statistically significant. Data are presented as the mean±s.e.m.; the number of experiments performed with independent cultures or animals (*n*) and *P*-values are indicated in the figures. Box and whisker plots show the mean (+), median (line in box), and maximum and minimum values (whiskers).

## Supplementary Material

10.1242/develop.200563_sup1 Supplementary information
