# Supplementary material for: IGF2 interacts with the imprinted gene Cdkn1c to promote terminal differentiation of neural stem cells
Source: Development. 2023 Jan 12;150(1):dev200563. doi: 10.1242/dev.200563 (PMC9903205; doi:10.1242/dev.200563)
Supplement: Supplementary information [file develop-150-200563-s1.pdf]

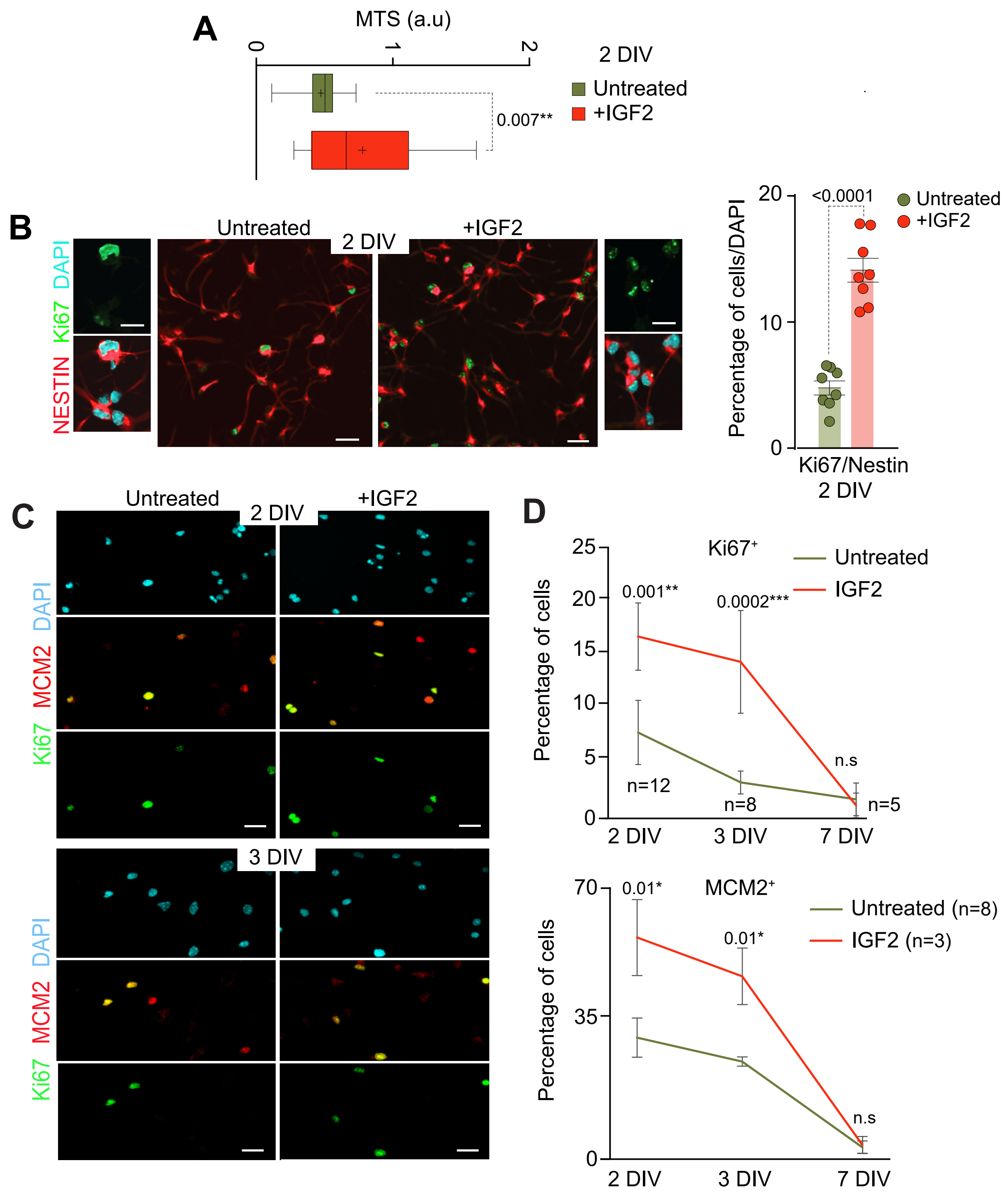

Fig. S1. Differentiation of NSCs with IGF2 promotes cell cycle maintenance . (A) MTS assay perfor-med after 2 DIV of differentiation in presence or absence of IGF2, measured as arbitrary units (a.u.). Boxes indicate interquartile range and whiskers indicate maximum and minimum values; n=20 and 13 experimental replicates respectively (two-tailed unpaired Student´s t-test). **(B)** Immunocytochemistry images for Nestin (red) and Ki67 (green) in NSCs after 2 DIV of differentiation in presence or absence of IGF2. High magnification images are also shown (left panel). Quantification of the percentage of Ki67/Nestin^{+} cells in NSCs after 2 DIV of differentia-tion in presence or absence of IGF2 (right panel). Data are mean±s.e.m; n=8 experimental replicates (two-tailed paired Student's t-test). **(C)** Immunocytochemistry confocal images for Ki67 (green), MCM2 (red) and DAPI (cyan) in NSCs after 2 and 3 DIV of differentiation in presence or absence of IGF2. **(D)** Percentage of cells positive for Ki67 and MCM2 in untrea-ted and IGF2-treated cultures after 2, 3 and 7 DIV in differentiation-promoting conditions. Data are mean±s.e.m; n=12, 8 and 5 experimental replicates for Ki67+ cells; n=8 and 3 experimental replicates respectively (Mann-Whitney test). DAPI was used to counterstain DNA. P-values and number of samples are indicated. Scale bars in B and C: 30 μm; High magnifi-cation in B: 10 μm.

Development • Supplementary information

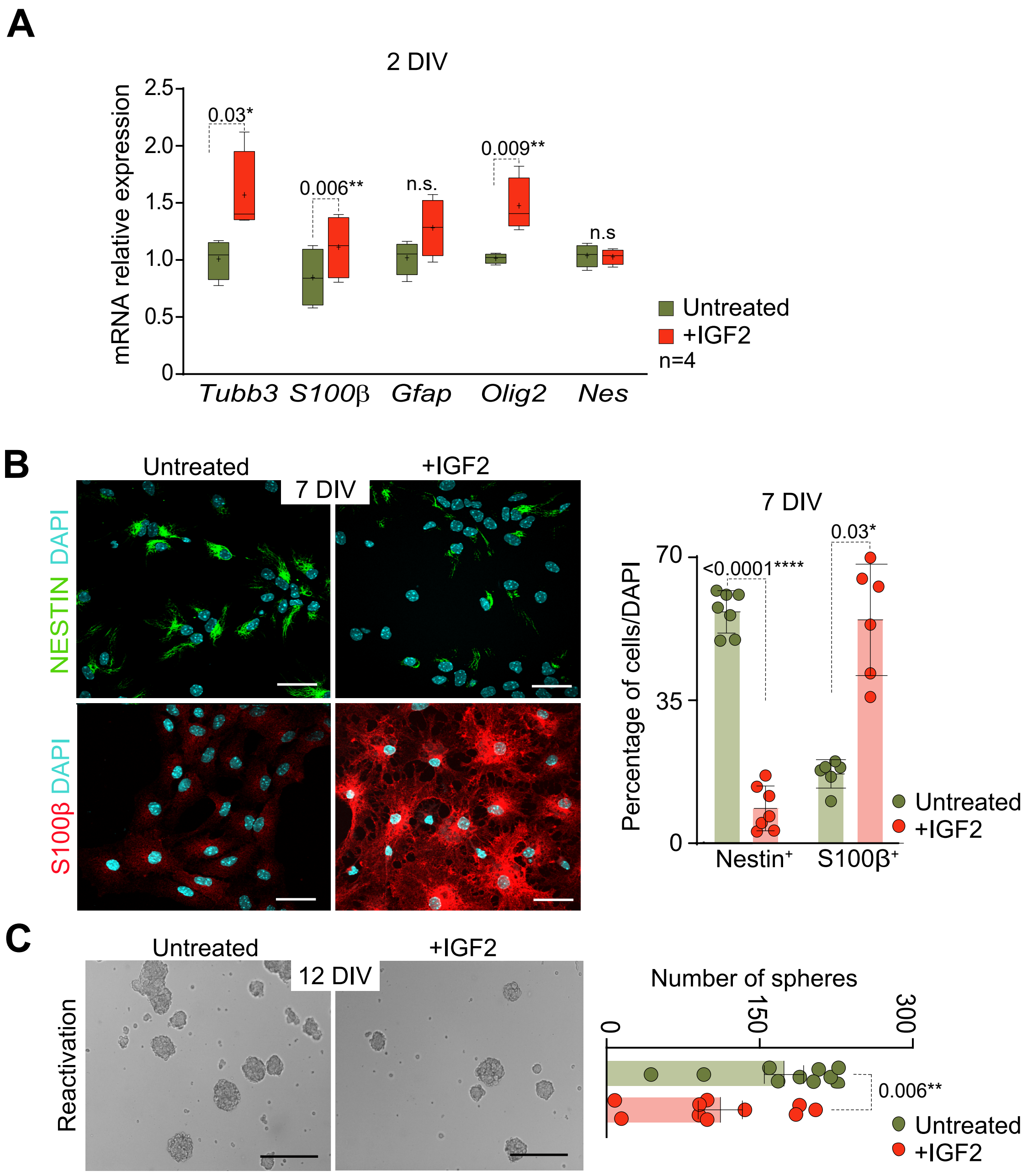

Fig. S2. IGF2 promotes terminal differentiation of NSCs. (A) qPCR for *βIII-tubulin* (*Tubb3*), *S100β*, *Gfap*, *Olig2* and *Nestin (Nes)* genes in NSCs after 2 DIV in differen-tiation conditions in presence or absence of IGF2. *Gapdh* was used as housekeeping gene. Boxes indicate interquartile range and whiskers indicate maximum and minimum values; n=4 experimental replicates (two-tailed unpaired Student´s t-test). **(B)** Immunocytochemistry images for Nestin (green) and S100*β* (red) in untreated and IGF2-treated cultures after 7 DIV of differentiation (left panel). Percentage of cells positive for Nestin and S100*β* after 7 DIV of differentiation in presence or absence of IGF2 (right panel). DAPI was used to counterstain DNA. Data are mean±s.e.m; n=6 experimental replicates (two-tailed paired Student´s t-test). **(C)** Representative images of neurospheres formed by differentiated cultures in the reactivation assay in presence or absence of IGF2 (left panel). Number of spheres formed after reactivation by detaching 7 DIV-differentiated NSCs and replating them for 5 more days in proliferation-promoting conditions in presence or absence of IGF2 (right panel). Data are mean±s.e.m; n=10 experimental replicates (Wilcoxon matched-pairs signed rank test). P-values and number of samples are indicated. Scale bars in B: 30 µm; in C: 100 µm.

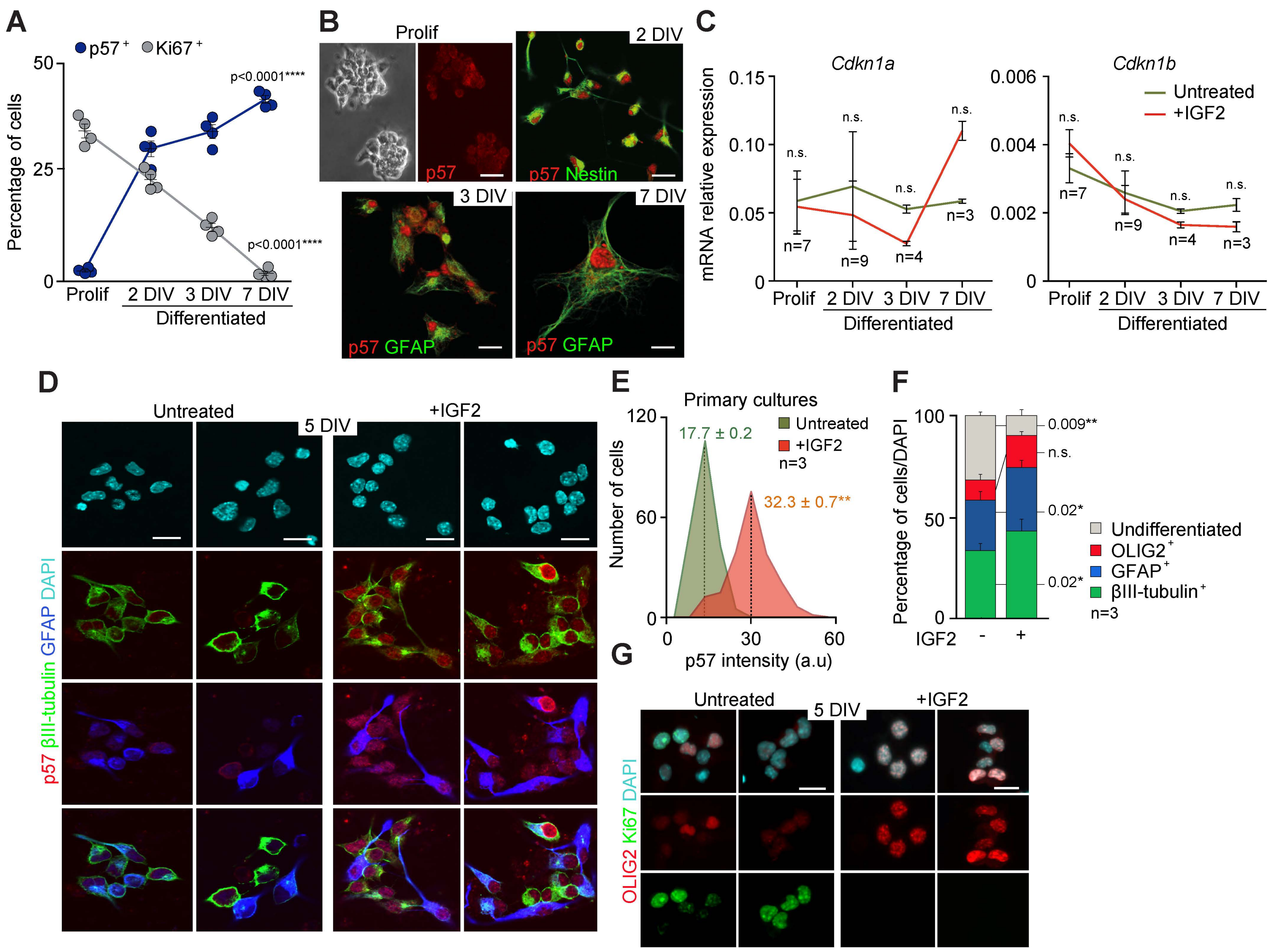

Fig. S3. IGF2 induces expression of p57 and differentiation of adult NSCs in primary cultures in the absence of mitogens. (A) Percentage of cells positive for p57 and Ki67 in NSCs cultures in proliferation conditions and after 2, 3 and 7 DIV of differentiation. Data are mean±s.e.m; n=4 experimental replicates (Two-way ANOVA with a post-hoc Tukey test). **(B)** Immunocytochemistry for p57 (red), Nestin (green) and GFAP (green) in NSCs in proliferation and after 2, 3 and 7 DIV of differentiation. **(C)** qPCR for *Cdkn1a* and *Cdkn1b* in NSCs in proliferation conditions and after 2, 3 and 7 DIV of differentiation. *Gapdh* was used as housekeeping gene. Data are mean±s.e.m; n=7, 9, 4 and 3 experimental replicates respectively (Wilcoxon matched-pairs signed rank test). **(D)** Immunocytochemistry images for p57 (red), *β*III-tubulin (green) and GFAP (blue) in primary cultures from the adult SVZ in absence or presence of IGF2 after 5 DIV of differentiation-promoting conditions. The percentage of undifferentiated cells is also determined. Data are mean±s.e.m; n=3 experimental replicates (two-tailed paired Student´s t-test). **(E)** Histograms showing p57 intensity (in arbitrary units, a.u) in primary cultures from the SVZ in presence or absence of IGF2 after 5 DIV of differentiation. **(F)** Percentage of cells positive for β III-tubulin, Olig2 or GFAP in untreated and in IGF2-treated cultures after 5 DIV of differentiation. The percentage of undifferentiated cells is also determi-ned. Data are mean±s.e.m; n=3 experimental replicates (two-tailed paired Student´s t-test). **(G)** Immunocytochemistry for OLIG2 (red) and Ki67 (green) in primary cultures isolated from the adult SVZ that have been treated or not with IGF2. DAPI was used to counterstain DNA. Number of samples and P-values are indicated. Scale bars in B, D and G: 30 µm.

Development • Supplementary information

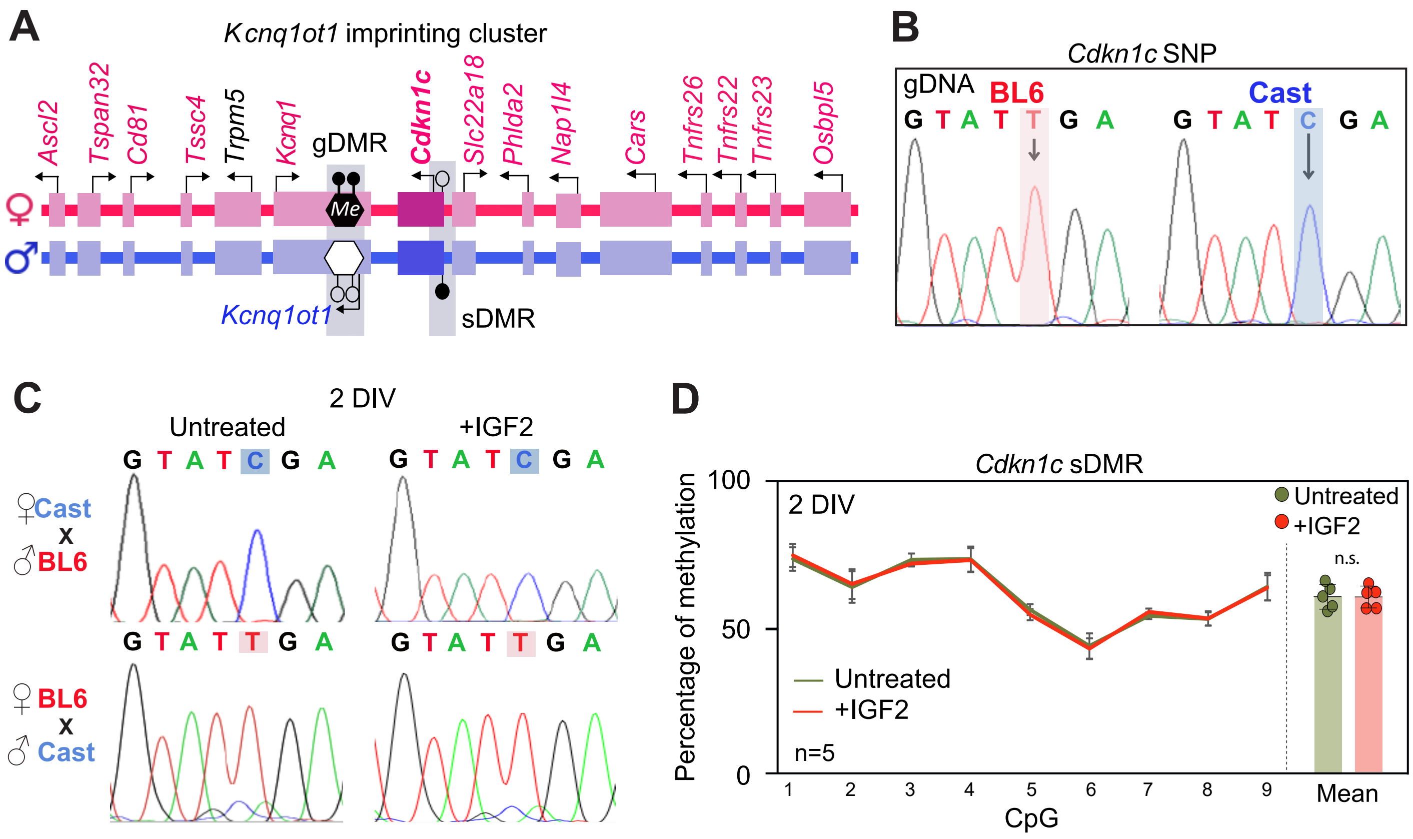

Fig. S4. Genomic imprinting of the *Cdkn1c* gene is maintained after IGF2 treatment. (A) Sche-matic of the *Cdkn1c* imprinted cluster. Expression of the maternally (pink) and paternally (blue) expressed genes within the cluster is shown. The methylation status of the *Cdkn1c* somatic DMR (sDMR) is illustrated with open and filled circles representing full methylation at the paternal allele and lack of metylation at the maternal allele, respectively. Germline-derived DMR (gDMR) is also represented. **(B)** Sequence analysis of gDNA from adult NSCs derived from *Mus musculus domesticus* (abbreviated BL6) and *Mus musculus castaneus* (abbreviated Cast) mice. Sequence traces show a polymorphism at the *Cdkn1c* gene (T/C). **(C)** Sequence analysis of RT-PCR products from adult NSCs derived from reciprocal F1 hybrids mice from BL6 and Cast (CastxBL6 and BL6xCast) after being treated or not with IGF2 in differen-tiation conditions. **(D)** Percentage of methylation determined by bisulfite sequencing and pyrosequencing at 9 CpG sites within the sDMR of *Cdkn1c* in untreated or IGF2-treated after 2 DIV of differentiation. Mean percentage of methylation is also indicated. Data are mean±s.e.m; n=5 experimental replicates (Mann-Whitney test). Number of samples is indica-ted.

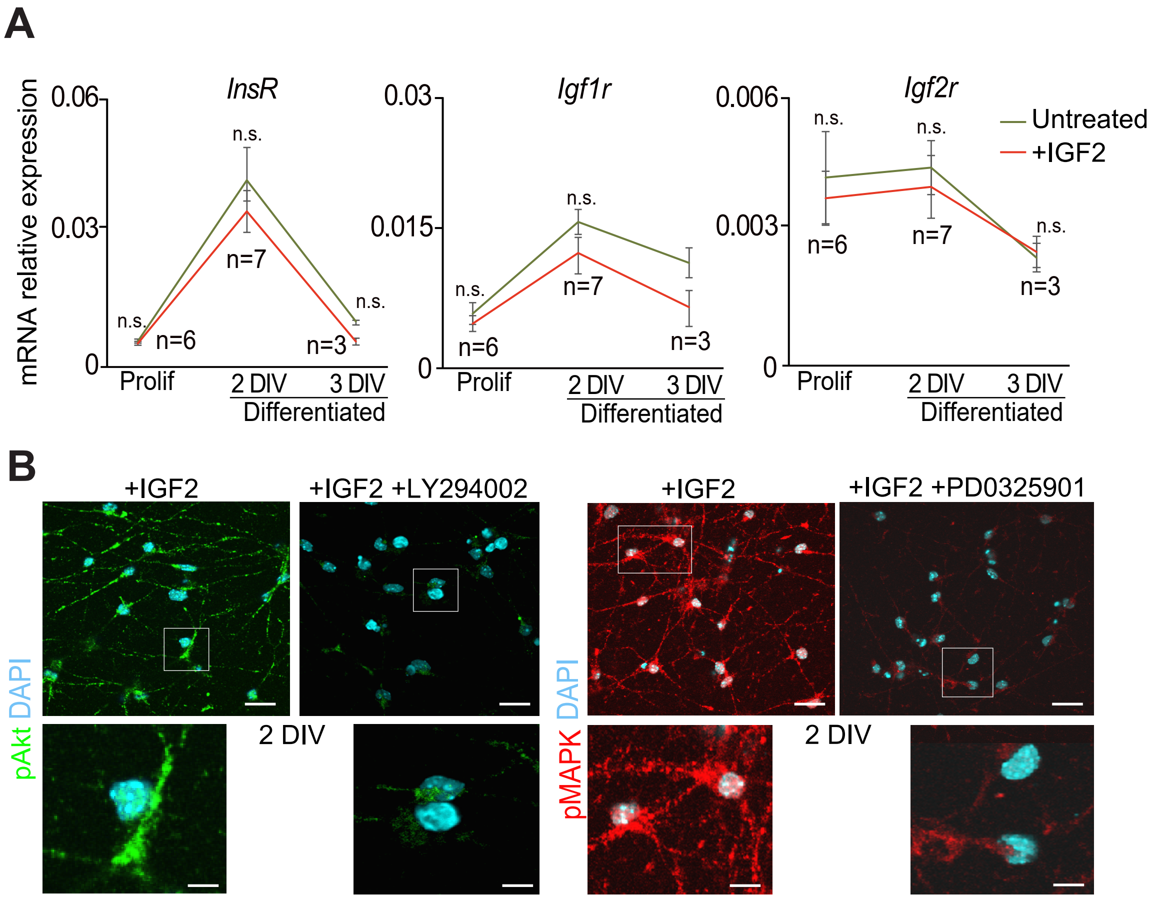

Fig. S5. Mechanism of action of IGF2 in NSCs during differentiation. (A) qPCR for insulin receptor (*Insr*), Igf1 receptor (*Igf1r)* and Igf2 receptor (*Igf2r)* genes in NSCs in proli-ferating conditions (Prolif) and after 2 and 3 DIV of differentiation in presence or absence of IGF2. *Gapdh* was used as housekeeping gene. Data are mean±s.e.m; n=6, 7 and 3 experimental replicates respectively (Mann-Whitney test). **(B)** Immunocytochemistry images for pAkt (green) and pMAPK (red) in NSCs after 2 DIV of differentiation in presence of IGF2 and after treatment with the PI3K inhibitor LY294002 or the ERK1/2 inhibitor PD0325901. DAPI was used to counterstain DNA. Scale bars in B: 30 μm (high magnification images in B: 10 μm).

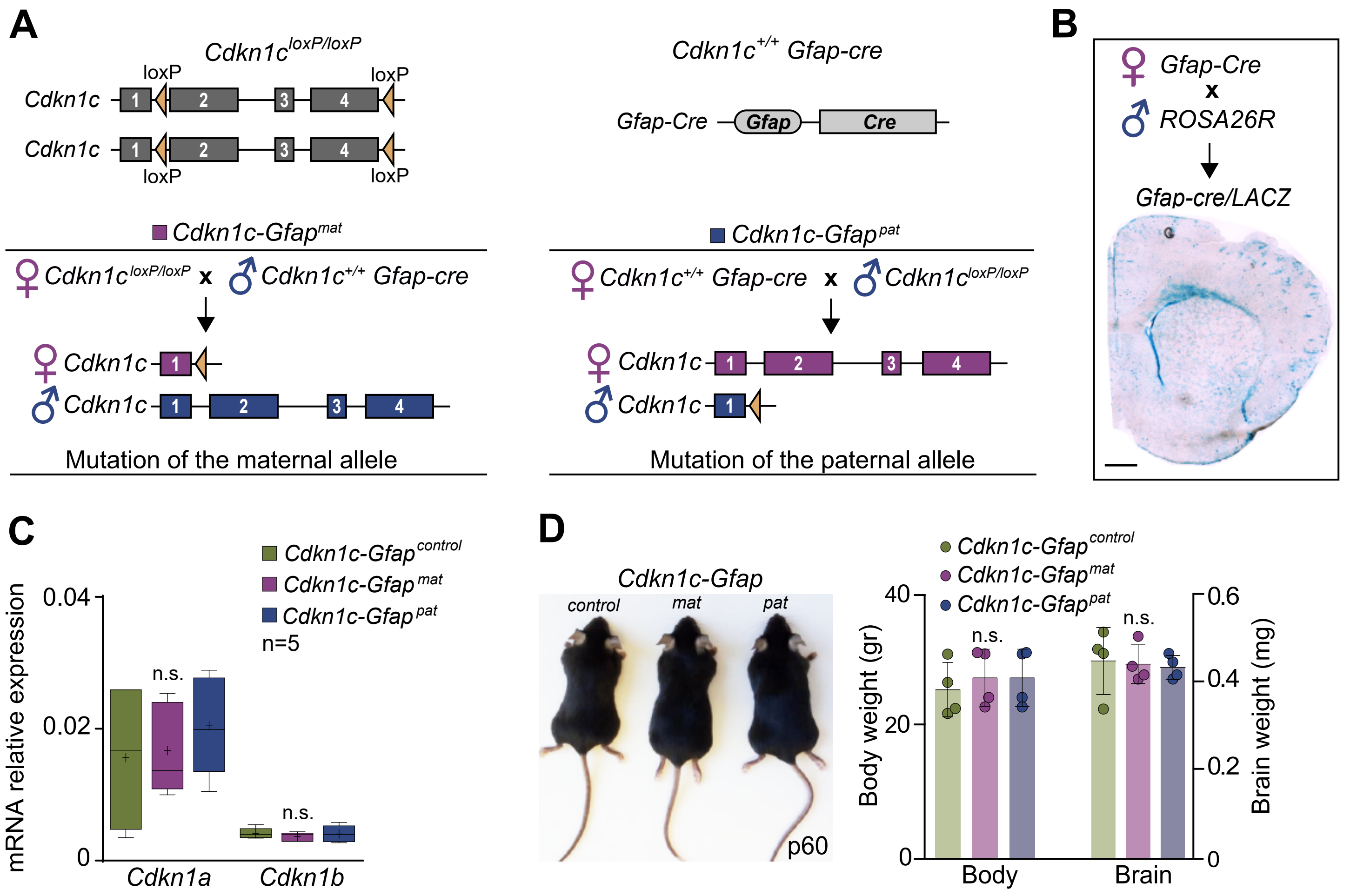

Fig. S6. Conditional deletion of *Cdkn1c* in a murine model. (A) Schematic of the conditional murine model. Mice with the mutation of *Cdkn1c* at the maternal allele (*Cdkn1c-Gfap*^{mat}) were generated by crossing *Cdkn1c* floxed females with GFAP-*cre* males. Reciprocally, mice with a mutation in the paternal allele (*Cdkn1c-Gfap*^{pat}) were generated by crossing GFAP-*cre* females with *Cdkn1c* floxed males. **(B)** β-galactosidase staining (blue) in the brain of Gfap-CRE/LACZ mice. **(C)** qPCR for *Cdkn1a* and *Cdkn1b* in *Cdkn1c-Gfap*^{control}, *Cdkn1c-Gfap*^{mat} and *Cdkn1c-Gfap*^{pat} NSCs in proliferation conditions. *Gapdh* was used as housekeeping gene. Boxes indicate interquartile range and whiskers indicate maximum and minimum values; n=5 experimental replicates (Two-way ANOVA with a post-hoc Tukey test). **(D)** Images of *Cdkn1c -Gfap*^{control}, *Cdkn1c-Gfap*^{mat} and *Cdkn1c-Gfap*^{pat} mice (left panel). Body and brain weights in postnatal day (p)60 mice from the three genotypes (right panel). Data are mean±s.e.m; n=4 experi-mental replicates (Two-way ANOVA with a post-hoc Tukey test). Number of samples and P-values are indicated.

Development • Supplementary information

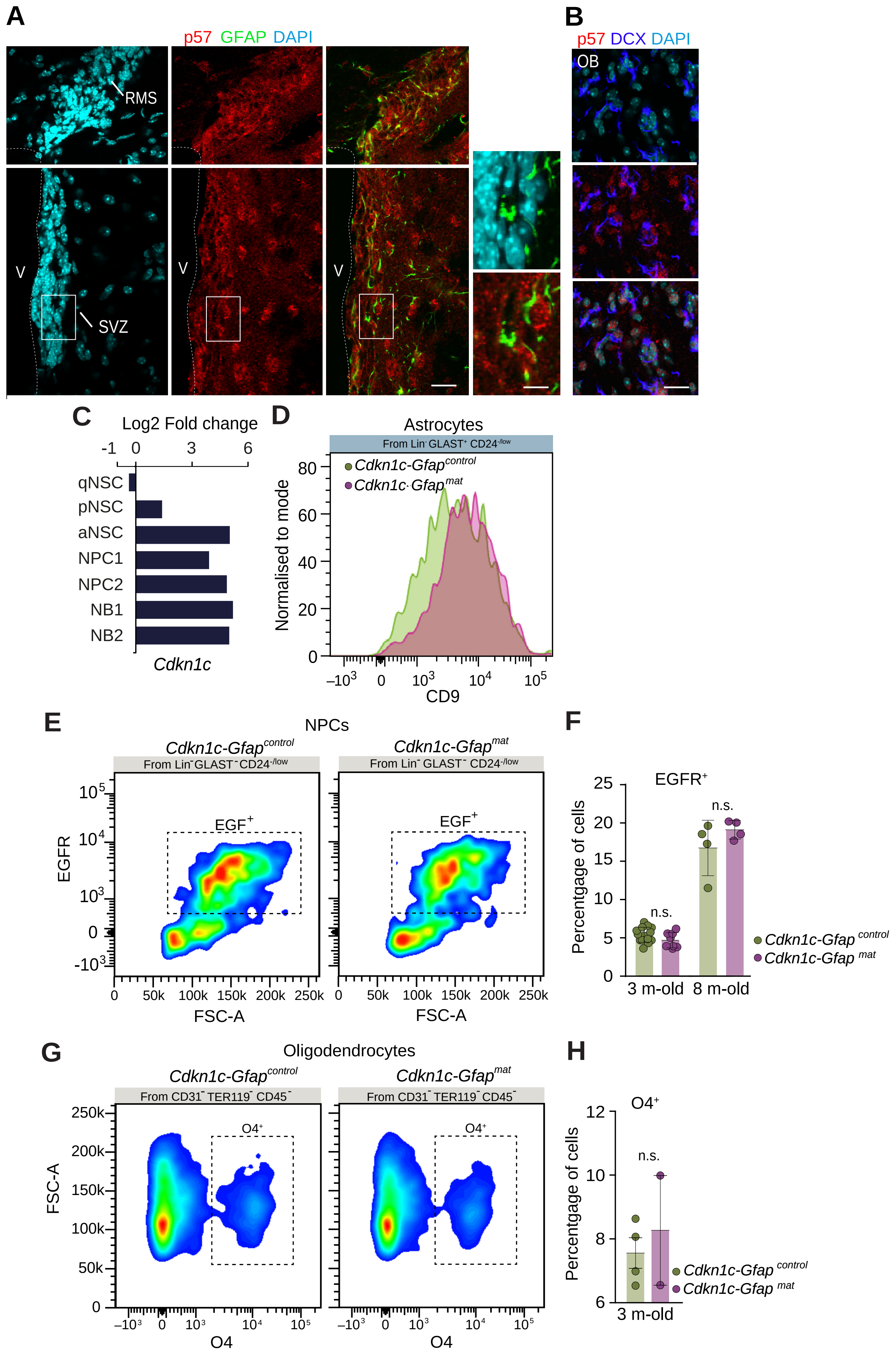

Development • Supplementary information

Fig. S7. p57 is expressed in the adult SVZ. (A) Immunohistochemistry confocal images for p57 (red) and GFAP (green) in the RMS and SVZ of wild-type mice. **(B)** Immunohistochemistry confocal images for p57 (red) and DCX (blue) in the OB of wild-type mice. **(C)** RNAseq data represented as Log2 Fold change for *Cdkn1c* in FACs isolated cells from the adult SVZ of wild-type mice. **(D)** Histograms showing CD9 intensity in cells isolated from the SVZ of *Cdkn1c-Gfap*^{control} and *Cdkn1c-Gfap*^{mat} mice. **(E)** Flow cytometry analysis of the EGFR^{+} neural progenitor (NPCs) population in the SVZ of *Cdkn1c-Gfap*^{control} and *Cdkn1c-Gfap*^{mat} mice. **(F)** Percentage of EGFR^{+} cells analysed by flow cytometry in the SVZ of 3 months-old and 8 months-old mice. Data are mean±s.e.m; n=8, 5, 4 and 4 experimental replicates respectively (Two-way ANOVA with a post-hoc Tukey test). **(G)** Flow cytometry analysis of the O4^{+} oligodendrocyte population in the SVZ of *Cdkn1c-Gfap*^{control} and *Cdkn1c-Gfap*^{mat} mice. **(H)** Percentage of O4^{+} cells analysed by flow cytometry in the SVZ of 3 months-old mice. DAPI was used to counterstain DNA. V: ventricular lumen. Data are mean±s.e.m; n=4 and 2 experimental replicates respectively (Mann-Whitney test). Scale bars in A and B: 30 µm (high magnifications in A: 10 µm).

Development • Supplementary information

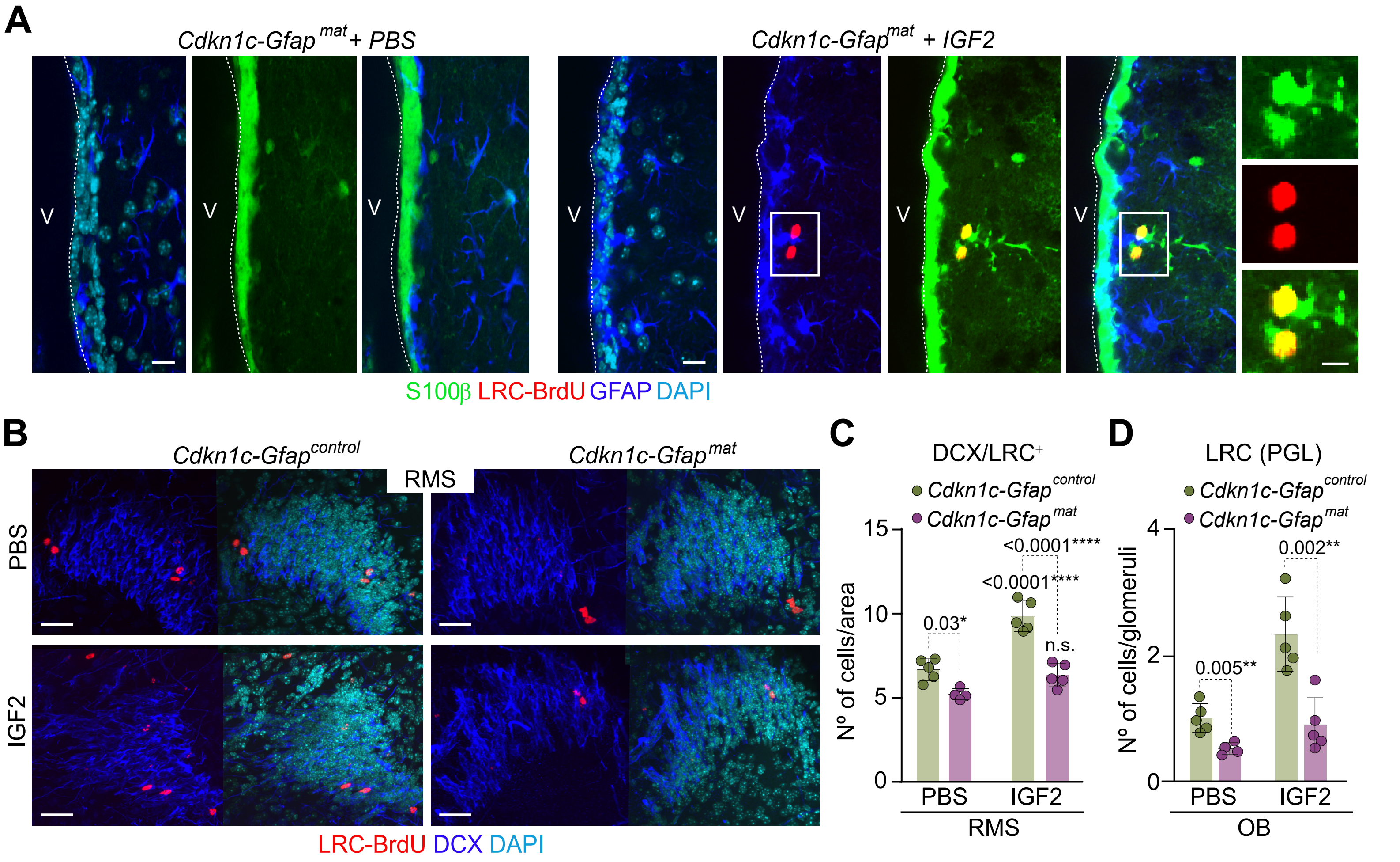

Fig. S8. Ventricular infusion of IGF2 induces astrocytic differentiation of NSCs. (A) Immunohistochemistry confocal images for S100β (green), GFAP (blue) and BrdU-LRC (red) in the SVZ of *Cdkn1c-Gfap*^{control} and *Cdkn1c-Gfap*^{mat} mice after infusion of PBS or IGF2. **(B)** Immunohistochemistry confocal images for BrdU-LRC (red) and DCX (blue) in the SVZ of *Cdkn1c-Gfap*^{control} and *Cdkn1c-Gfap*^{mat} mice after infusion of PBS or IGF2. **(C)** Quantification of the number of DCX/BrdU-LRC^{+} neuroblasts migrating through the RMS of *Cdkn1c-Gfap*^{control} and *Cdkn1c-Gfap*^{mat} mice after the infusion of PBS or IGF2. Data are mean ±s.e.m; n=8, 5, 4 and 4 experimental replicates respectively (Two-way ANOVA with a post-hoc Tukey test). **(D)** Quantification of the number of BrdU-LRC^{+} cells in the periglomerular layer (PGL) of the OB in *Cdkn1c-Gfap*^{control} and *Cdkn1c-Gfap*^{mat} mice after infusion of PBS or IGF2. DAPI was used to counterstain DNA. Data are mean±s.e.m; n=5 experimental replicates (Two-way ANOVA with a post-hoc Tukey test). V: ventri-cular lumen. P-values are indicated. Scale bars in A and B: 30 µm (high magnification in A: 7 µm).

Development • Supplementary information

Table S1. List of primers used.

Gene	Sequence (5'-3')	Application
p57 CKO-F	AAGCTGGACAGGACAAGCGATCC	Genotyping
p57 CKO-R	ATGGTCGAAGGCTGTGCAAACGC	Genotyping
p57 3'-Rv	GTTCTCGCACACAAACTAGATCAG	Genotyping
Cdkn1cMETH-F	AGGATTTAGTTGGTAGTAGTAGG	Methylation study
Cdkn1cMETH-R	AACCATAAAACTAAACACAACCCC	Methylation study
Cdkn1cMETH-seq	GGTGTAGTTTTAGGGTTAG	Methylation study
Cdkn1cSNP-F	TAGCAGGAACCGGAGATGG	SNP Sequencing
Cdkn1cSNP-R	ACACCTTGGGACCAGCGTACT	SNP Sequencing
Cre-F	GCGGTCTGGCAGTAAAAACTATC	Genotyping
Cre-R	GTGAAACAGCATTGCTGTCACTT	Genotyping

Table S2. List of primary antibodies used.

Antibody	Source	Host	Dilution	Cat #	Application
βIII-tubulin	Covance	Mouse	1/300	PRB-435P	ICC
BrdU	Abcam	Rat	1/600	Ab6326	IHC
CD24-PerCP-Cy5.5	BD Biosciences	Rat	1/300	562360	FC
CD31-BUV421	BD Biosciences	Rat	1/100	563356	FC
CD45-BUV421	BD Biosciences	Rat	1/200	563890	FC
CD9-Vio770	Miltenyi	Rat	1/20	130-102-384	FC
DCX	Santa Cruz	Mouse	1/500	sc-271390	IHC
GAPDH	Millipore	Mouse	1/5000	MAB374	WB
GFAP	Dako	Rabbit	1/600	Z0334	ICC/IHC
GFAP	Millipore	Chicken	1/600	AB5541	ICC/IHC
GLAST-PE	Miltenyi	Mouse	1/20	130-095-821	FC
IGF1R	Cell Signaling	Rabbit	1/500	3027	WB
IGF2R	Abcam	Mouse	1/1000	ab2733	WB
IR (4B8)	Cell Signaling	Rabbit	1/200	3025	WB
Ki67	Abcam	Rabbit	1/100	ab15580	ICC/IHC
MCM2	Santa Cruz	Goat	1/300	Sc-9839	ICC
Nestin	Hybridoma Bank	Mouse	1/4	rat-401	ICC
O4	Hybridoma Bank	Mouse	1/2	rip	ICC
O4-405	R&D	Rat	1/50	FAB1326V	FC
OLIG2	Millipore	Rabbit	1/300	AB9610	ICC
p57	Sigma	Rabbit	1/1000	SAB4500071	WB/ICC/IHC
pAkt (Ser473)	Cell Signaling	Rabbit	1/2000	9271	ICC
PathScan Multiplex WB cocktail I	Cell Signaling	Rabbit	1/400	5301	WB
pYR1161IGFIR	Abcam	Rabbit	1/400	ab5681	WB
pY972IR	Abcam	Rabbit	1/500	ab5678	WB
pMAPK (Erk1/2)	Cell Signaling	Rabbit	1/3000	9102	ICC
S100β	Dako	Rabbit	1/300	Z0311	ICC/IHC
tAkt	Cell Signaling	Rabbit	1/1000	4691	WB
Ter119-BUV421	BD Biosciences	Rat	1/200	563998	FC
tMAPK	Cell Signaling	Rabbit	1/1000	9102	WB

ICC, Immunocytochemistry IHC, Immunohistochemistry WB, Western-blot FC, Flow cytometry

Table S3. List of secondary antibodies used.

Antibody	Source	Dilution	Cat #	Application
Alexa Fluor® 488 Donkey Anti-Chicken	Jackson ImmunoResearch	1/600	703-545-155	ICC/IHC
Alexa Fluor® 488 Donkey Anti-Mouse	Molecular Probes	1/600	A-21202	ICC/IHC
Alexa Fluor® 488 Donkey Anti-Rabbit	Jackson ImmunoResearch	1/600	711-547-003	ICC/IHC
Alexa Fluor® 647 Donkey Anti-Chicken	Jackson ImmunoResearch	1/600	703-605-155	ICC/IHC
Alexa Fluor® 647 Donkey Anti-Mouse	Jackson ImmunoResearch	1/600	715- 605-151	ICC/IHC
Alexa Fluor® 647 Donkey Anti-Rabbit	Jackson ImmunoResearch	1/600	711-607-003	ICC/IHC
Alexa Fluor® 647 Donkey Anti-Goat	Jackson ImmunoResearch	1/600	705-605-003	ICC/IHC
Biotinylated Horse Anti-Mouse	Vector	1/1000	BA2000	ICC
Cy3-Donkey Anti-Mouse	Jackson ImmunoResearch	1/800	715-165-151	ICC/IHC
Cy3-Donkey Anti-Rabbit	Jackson ImmunoResearch	1/800	711-165-152	ICC/IHC
Cy3-Donkey Anti-Rat	Jackson ImmunoResearch	1/600	712-165-153	ICC/ICH
Cy3-Streptavidin	Jackson ImmunoResearch	1/2000	016-160-084	ICC
Goat Anti-Mouse IgG-HRP	Dako	1/5000	P0447	WB
Goat Anti-Rabbit IgG-HRP	Santa Cruz	1/5000	sc-2004	WB

ICC, Immunocytochemistry IHC, Immunohistochemistry WB, Western-blot

Table S4. List of Taqman probes used.

Gene	TaqMan Code (Applied Biosystems)
Cdkn1a	Mm04205640_g1
Cdkn1b	Mm00438168_m1
Cdkn1c	Mm01272135_g1
Gapdh	Mm99999915_g1
Gfap	Mm01253033_m1
Igfr1	Mm00802831_m1
Igfr2	Mm00439576_m1
Insr	Mm01211875_m1
Nes	Mm00450205_m1
Olig2	Mm01210556_m1
S100β	Mm00485897_m1
Tubb3	Mm00727586_s1
