# Supplementary material for: IGF2 interacts with the imprinted gene Cdkn1c to promote terminal differentiation of neural stem cells
Source: Development. 2023 Jan 12;150(1):dev200563. doi: 10.1242/dev.200563 (PMC9903205; doi:10.1242/dev.200563)
Supplement: Supplementary file 1 [file develop-150-200563_review_history.pdf]

IGF2 interacts with the imprinted gene *Cdkn1c* to promote terminal differentiation of neural stem cells

Anna Lozano-Ureña, Laura Lázaro-Carot, Esteban Jiménez-Villaba, Raquel Montalbán-Loro, Isabel Mateos-White, Pere Duart-Abadia, Irene Martínez-Gurrea, Keiichi I. Nakayama, Isabel Fariñas, Martina Kirstein, Cristina Gil-Sanz and Sacri R. Ferrón
DOI: 10.1242/dev.200563

Editor: Francois Guillemot

Review timeline
First submission: 26 January 2022
Editorial decision: 1 March 2022
Rebuttal received: 24 May 2022
Rebuttal decision: 27 May 2022
First revision received: 26 October 2022
Accepted: 23 November 2022

Original submission

First decision letter

MS ID#: DEVELOP/2022/200563

MS TITLE: Imprinted genes Cdkn1c and Igf2 interact to promote terminal differentiation of adult NSCs

AUTHORS: Anna Lozano-Urena, Esteban Jimenez-Villaba, Laura Lazaro-Carot, Raquel Montalban-Loro, Irene Martinez-Gurrea, Keiichi I. Nakayama, Martina Kirstein, and Sacri R Ferron

Dear Dr. Ferron,

I have now receivedthe reports of three referees on your manuscript and I have reached a decision. I am sorry to say that the outcome is not a positive one. The referees' comments are appended below, or you can access them online: please go to Development's submission site and click on the 'Manuscripts with Decisions' queue in the Author Area.

As you will see, two of the referees express significant concerns about your paper and are not strongly in favour of publication. In particular, referee 1 comments on the lack of sufficient novelty of the conclusions of the study, and referee 2 criticises the experimental design relying entirely on neurospheres and their treatment with EGF and FGF2. Having looked at the manuscript myself, I agree with their views and after careful consideration I have decided to reject your paper.

I do realise this is disappointing news, but Development receives many more papers than we can publish, and we can only accept manuscripts that receive strong support from referees.

I do hope you find the comments of the referees helpful, and that this decision will not dissuade you from considering Development for publication of your future work. Many thanks for sending your manuscript to Development.

© 2023. Published by The Company of Biologists under the terms of the Creative Commons Attribution License (https://creativecommons.org/licenses/by/4.0/).

Reviewer 1

Advance summary and potential significance to field

In the manuscript presented by Lozano-Ureñ et al., the authors investigate the effects of IGF2 on terminal differentiation of adult Neural Stem Cells (NSCs) and its interaction with the imprinted gene Cdkn1c. Using differentiating neurospheres based cultures, they show that an addition of IGF2 promotes NSCs terminal differentiation and upregulates Cdkn1c gene expression. Investigating the regulation of Cdkn1c expression by IGF2, they show that IGF2 action is not linked to a loss of imprinting of Cdkn1c but rather to the activation of the PI3K-Akt pathway. Furthermore, they show that inactivation of p57, the cyclin-dependent kinase inhibitor encoded by Cdkn1c, alters the effects of IGF2 on NSCs terminal differentiation.

In general, the experiments are well designed and controlled,the figures are thorough. The manuscript contains publishable data but the impact of the results is limited. For instance, the link between IGF2 and p57 expression via PI3K-Ekt pathway is not new as it has been already demonstrated in Thomas et al. (2016) in the hematopoietic stem cells like the authors mentioned in the manuscript.

Comments for the author

The title is somewhat misleading, as epigenetics is not involved in the interaction of Cdkn1c and IGF2. For instance, the imprinted nature of IGF2 gene is not implicated in the function described here nor in its interaction with Cdkn1c. On the same line, the results do not show a regulation of Cdkn1c by IGF2 via a regulation of its methylation. Furthermore, the manuscript exhibits many redundancies between the introduction and the discussion, and ideas are repeated even within the discussion.

Specific points:
- In the title of the second part “IGF2 induces the expression of the maternally expressed gene….”, besides the redundancy of wording, the term “maternally“ is misleading as the maternally imprinting is not a subject of this part.

- In the text, figure 4e illustrates the following claim: “the proportion of cells with the capacity to still form neurospheres in non-adherent cultures after differentiation was again reduced after IGF2 treatment in wild-type and Cdkn1c-Gfappat but not in Cdkn1c-Gfapmat cultures (Fig. 4e).” However, the graph shows a significant reduction of the number of spheres obtained from cdkn1c-GFAPmat genotype after IGF2 treatment compared to the untreated condition. This sentence should be changed.

- For the statistical analysis, the authors used t-test when comparing two groups which is not adequate when the sample is <30. A non-parametric test like Mann-Whitney U test or Wilcoxon Signed Rank test should be used instead.

- To help the reading, the meaning of the “n” values that are integrated in the figures must be added in the captions.

Reviewer 2

Advance summary and potential significance to field

This research article investigates the role of IGF2 in differentiation of neurosphere-derived cells from adult SVZ in vitro. The results show that IGF2 increases differentiation of both neuronal and glial cells from neurospheres and that this differentiation is not dependent on genomic imprinting but on p57-dependent signaling. IGF2 conveys its effects on p57 via Akt/Erk kinase pathway.

This is an interesting, additive research article that helps to refine the understanding of the role of IGF2 in the biology of adult neural stem cells. The most substantial novelty contribution of the article to the field is not the underlying connection between IGF2 signaling and p57 in

© 2023. Published by The Company of Biologists under the terms of the Creative Commons Attribution License (https://creativecommons.org/licenses/by/4.0/).

differentiation but the finding that unlike cell maintenance roles of IGF2, its function in cell differentiation does not require genomic imprinting. Unfortunately, this most surprising finding is not explored beyond the initial description. While this study is intriguing, it suffers from several major conceptual flaws. However, if these flaws are corrected by a major revision, the publication may have more significant impact on the fields of neurogenesis, genomic imprinting and IGF2 signaling.

Comments for the author

Major concerns:

1. The entire study is performed on neurospheres or neurosphere-derived cell cultures. While useful for discrete purposes, the neurosphere assay developed by Reynolds and Weiss displays many artifacts and limitations, already recognized in early 2000s (see review by Jensen and Parmar, Mol. Neurobiol. 2006, for example). To ensure that their findings in vitro are relevant, the authors should replicate the IGF2-dependent pro-differentiation effects in vivo, ideally by administration of IGF2 by osmotic mini-pumps to the lateral ventrices of mice or rats. In addition, experiments on cell differentiation should be replicated on primary adult NSC cultured in monolayer (Costa et al., Development 2011) without previous exposure to EGF/FGF2 (more below). Primary cell cultures suffer from fewer artifacts than neurospheres.

2. Exposure of primary neurospheres to EGF/FGF2 may introduce artifacts and change sensitivity of neurosphere-derived cells to mitogens, including IGF2 which can alter IGF2-dependent effects and interpretation of data. The authors should avoid this pitfall by replicating some of their experiments (such as IGF2-dependent cell pro-differentiation and increased Cdkn1c expression) on neurosphere-derived cells by experiments on primary cell cultures not exposed to EGF/FGF2.

3. The entire article stands on the assumption that SVZ neurospheres and neurosphere-derived cells express receptors for IGF2. But this is not directly showed. The authors should show that the cells express IGF2R (or insulin receptor) and how this expression changes with IGF2 administration. Without this evidence, it is hard to accept the results or to know if IGF2 in the used concentration (10x EC50 – see minor points below) activates its receptors or non-specifically activates different targets.

4. Statistical treatment of data is problematic. The data are analysed by parametric statistical tests, however, it is not indicated if data were not normally distributed or even if normality was tested. In fact, in many cases the data sets contain only 3-4 biological replicates, which does not allow proper testing of normality even with the Kolmogorov-Smirnov Test. First, the normal distribution of data must be tested where possible ($N > 5$). Normally distributed data sets can be tested with parametric statistical tests. Where testing of normal distribution of data is not possible or not warranted ($N<5$), parametric tests must be replaced by non-parametric tests (e.g. Kruskal-Wallis instead of ANOVA, Mann-Whitney instead of T-Test etc.). Applying parametric tests when not appropriate increases false failure of rejecting the null hypothesis (e.g. Type II error).

Minor concerns:
1. It is not clear what the null hypothesis of the article is? This should be stated explicitly.

2. Concentration of IGF2 used in this article is 100 ng/ml. Molecular weight of IGF2 is 632 (or around 20 kDa) and its EC50 to IGF2R is reported to be around 20 nM. 100 ng/ml corresponds to about 160 nM – 10 times more than its EC50. This seems like unnecessarily high concentration. As a proof of principle, the authors should show that even 20 nM IGF2 can induce its differentiation effects to avoid non-specific artifacts.

3. Martigel used for culturing neurosphere-derived cells might caused small proportion of pro-neuronal differentiation (almost zero in control condition in Fig.1f). This almost zero differentiation to bIII-Tub cells is disconcerting. A solution could be growing cells without Matrigel as primary cell cultures (see major concerns above).

4. Given the extremely low expression of Tuj1 in Fig.1d raises a question of how specific the RT-qPCR primers were. Was the primer specificity tested?

© 2023. Published by The Company of Biologists under the terms of the Creative Commons Attribution License (https://creativecommons.org/licenses/by/4.0/).

5. What was the cut-off for delta Ct values for qPCR? If it was above 35, such expression data suffer from artifacts (e.g. primer-dimer driven amplification) and lack biological meaning.

6. Were qPCR data analyzed as delta-delta Ct as described by Livak and Schmittgen, Methods 2001? What was the housekeeping gene the expression was compared to?

7. In support to the major concern 2, there is the result, which shows that withdrawal of EGF increases Cdkn1c expression, suggesting that EGF artificially suppresses Cdkn1c expression. This result calls for replicating the Cdkn1c expression experiment in the absence of EGF and in primary cell cultures.

8. What cell identify the control neurosphere-derived cells in Fig.1 had? Since there were no bIIItubulin+ and almost no O4+, S100b+ and GFAP+, what were the control cells? One would expect more GFAP+ NSC-like cells but Fig.1e shows very few of these as well. The authors should quantify number of GFAP+/Nestin+ (or Vimentin+) NSCs in their cultures.

9. While the summary results in Fig.2c show a very small increase of p57 protein levels in cells treated with IGF2, the representative blots are not convincing. With N=4, I am concerned that this increase is an artifact of inappropriately used parametric statistical tests. The rather small increase in p57 protein levels does not correspond to quite robust increase in Cdkn1c mRNA in Fig.2a. All data should be re-tested with non-parametric tests where appropriate, but it is especially important for data in Fig.2c.

10. Third paragraph in the introduction states "The potential role of p57 in the regulation of neurogenesis in the adult SVZ niche has not been addressed". This is not true. p57 has been implicated in NSC proliferation in the SVZ in the context of ischaemia and repetitive magnetic stimulation (Guo et al., PLoS ONE 2014). This should be corrected and this article should be cited.

11. Given the regulation of p57 expression by miR-25, and co-current regulation of p57 and PTEN, it would be useful to see if IGF2 administration changes expression of both miR-25 and PTEN.

12. When showing results from cells in differentiating conditions, it is not clear if all these experiments followed the protocol as described in Fig.1a. This should be made clear.

13. In figure 3, there is a missing control of cells treated with Akt and/or Erk inhibitors but in the absence of IGF2 administration. Alternatively, stimulation of phosphorylation of Akt/Erk should replicate IGF2 effects on differentiation.

14. How do authors explain the discrepancy between their previous work (Ferron et al., Nat. Comm. 2015), which shows that genomic imprinting is essential for IGF2-dependent cell maintenance of adult NSCs from SVZ, yet their results in this study (Fig.4) show that IGF2 role in cell differentiation in vitro is not dependent on genomic imprinting?

<u>Reviewer 3</u>

Advance summary and potential significance to field

The role of the imprinted gene Cdkn1c/p57 in the regulation of neurogenesis in the adult Sub Ventricular zone (SVZ) niche is unknown. Using a number of elegant experiments the authors conclude that p57 acts downstream of IGF2 signalling to control the differentiation programme of adult neural stem cells.

Comments for the author

The paper is well written and presented. There are a number of issues that I feel will be important to address prior to potential publication:

© 2023. Published by The Company of Biologists under the terms of the Creative Commons Attribution License (https://creativecommons.org/licenses/by/4.0/).

Major points:
1. The conclusion that IGF2 downstream signalling effects on Cdkn1c expression are not mediated by ERK1/2 is entirely based on the expression of Cdkn1c not being reduced at mRNA level after exposure to MEK/ERK inhibitor PD0325901. This is in spite of ERK1/2 increased activity upon IGF2 stimulation, measured by phosphorylation (shown in 2 c; left panel. The data presented for this inhibitor (shown in figure 3d) are highly variable. Given these issues, it is important to add a 'positive' control that shows that inhibition of ERK1/2 by PD0325901 at the dose applied is in fact occurring in these cells. Using Igf2 mRNA levels as the only readout is not adequate and for completeness, the authors should also consider adding differentiation data on IGF2+PD0325901 to Figures 3 e) and f).
The authors should also quantify the total levels of AKT and MAPK against respective loading controls and add this information as supplementary information, with corresponding statistical analysis. It seems to me, by visual inspection of the western blots. that both total AKT and MAPK levels are reduced upon IGF2 treatment. This is important as an increase in activity via phosphorylation may not be of significance if the total levels of these proteins are reduced.

2. What are the levels of expression of p57 protein in the 'untreated' conditions during the time course of the experiment? Concerning this point There is a mention to 'mitogen withdrawal inducing a 4 fold mRNA increase in Cdkn1c as measured at 2, 3, 7 DIV compared to proliferation conditions (Fig. 2a)' but at 2 DIV it looks more like a 2 fold increase (please add an extra reference point in Y axis) and at 3 and 7 DIV there is hardly Cdkn1c mRNA expression? Question remains on levels of p57 protein are at 3 DIV and 7 DIV in untreated conditions and how they relate to mRNA expression in Fig 2a. I would like to suggest to add a panel of p57 protein measurements for untreated conditions (mostly composed by undifferentiated cells) to Figure 2b for time points 2DIV, 3 DIV and 7 DIV (although for 2 DIV they are already shown in Fig 2d). Alternatively, this data on untreated conditions could be added as an extra-plot in Supplementary figure 1b?

3. Related to the point above, the increase of Cdkn1c in untreated conditions at 2 DIV, albeit small, occurs without IGF2 treatment, which raises the question of possible IGF2 independent actions of Cdkn1c. It would be beneficial to add a section in the discussion on the limitations of the study and future directions. For example, the experimental strategy followed here somewhat limits the investigation of Cdkn1c to IGF2-dependent actions, and as such potentially important IGF2-independent roles are not addressed. These could be presumably addressed in other models of differentiation that are not related to exogenous levels of IGF2. In that regard transient transfections of Cdkn1c could be of value to define any IGF2 independent roles?
There is a degree of repetition between Introduction and Discussion, so discussing the results more broadly would be beneficial.

Minor points:
1.It is clear from the knockout study (Figs 4d,e) that the actions of IGF2 are only partially mediated by Cdkn1c and as such this should be made clear in abstract and throughout.

2. Further experiments looking specifically at cell cycle stages would be required to substantiate the claim, mentioned in abstract for example, that the increase in IGF2 signalling and Cdkn1c lead to cell cycle exit. Although this is indeed very likely, it was not formally shown. Caution should be applied therefore, and advisable to tone down that conclusion by stating that the current data suggests that this might be the case.

3.The authors found that the increase of Cdkn1c mRNA expression is derived from the active maternal allele, i.e. does not involve loss of imprinting. As such stating that they have identified a methylation independent mechanism of control of Cdkn1c expression is inadequate.

4. Please add details about the specificity of the Cre line- i.e. where is it expressed? - and add the potential limitations of the Cre line if any.

5. Related to the Cre line it is stated in Methods that 'in these mice Gfap expression is prevented by the removal of a small region in exon 1' - what does that mean? Please add further information.

6. The authors should consider showing individual points in all graphs when N<10.

© 2023. Published by The Company of Biologists under the terms of the Creative Commons Attribution License (https://creativecommons.org/licenses/by/4.0/).

7. Please specify the precise numbers used in all experiments - e.g. stating N>4; N>=6 is not ideal. The full disclosure of numbers can be done in figures figure legends and/or in a dedicated supplementary table but should not be ambiguous. Figure 2c and 3C indicates n=4 in graph but only 3 blots are shown in supplementary 5 - therefore, is it n=4 or n=3?

8. Please clarify what is meant by normalized counts in Fig 2D - normalised to what precisely? and what does counts mean? please state the number of cells that were analysed in both groups (given that there are obvious differences in cell proliferation)
9. Supplementary Figure 4 could actually be added as a Main Figure as it is a nice summary/model of the work.

10. Please add the references to the phospho AKT and MAPK antibodies to table S4 (I can only see total ones mentioned)

11. Please specify the genetic background of the non-transgenic mice used in the study 12. There are a few typos - e.g. IFG2 instead of IGF2; differentaton instead of differentiation.

Author rebuttal letter

Dear Editorial Board

We write in response to the decision made on our manuscript entitled *"Imprinted genes Cdkn1c and Igf2 interact to promote terminal differentiation of adult NSCs"*. We are very grateful to the reviewers for their positive feedback, helpful comments and useful suggestions.

To replicate the IGF2-dependent effects *in vivo*, the reviewers proposed the IGF2 infusion in the lateral ventricles of control and *Cdkn1c* deficient mice. We have performed this experiment very recently and, consistently with the *in vitro* data, the results show that IGF2 promotes terminal differentiation of NSCs in a *Cdkn1c*-dependent manner. Infusion of IGF2 in *Cdkn1c* deficient mice generates a higher proportion of terminally differentiated astrocytes, at expenses of neuronal and oligodendroglial differentiation. IGF2 also promotes proliferation of NSCs within the niche but these proliferative effects are independent of *Cdkn1c*. Moreover, using flow cytometry and lineage tracing experiments we have confirmed that the deletion of *Cdkn1c* in NSCs from the SVZ *in vivo*, initially reduces the number of activated NSCs by direct differentiation into terminally differentiated astrocytes, also at expenses of neurogenesis and oligodendrogenesis, however these effects are exacerbated after infusion of IGF2.

We believe that these results address the main reviewer´s concerns and these new data will make the work of a much higher impact and quality. Other minor comments from reviewers will be easily addressed.

All the points raised by the reviewers are listed below with clarifications and a set of Figures including the new data is also appended.

In light of the comments above, we would be most grateful for the opportunity to make a further revision to the manuscript. We would be happy to include the additional data now provided and rearrange the data in a more comprehensive way and make clarifications to the text.

We hope that you agree with the points we have raised and will reconsider your original decision.

Many thanks for your consideration,

Sacri R. Ferrón

Note: Point-by-point response to reviewers comments can be found under **First revision:** Author response to reviewers' comments

---

© 2023. Published by The Company of Biologists under the terms of the Creative Commons Attribution License (https://creativecommons.org/licenses/by/4.0/).

Rebuttal response letter

MS ID#: DEVELOP/2022/200563

MS TITLE: Imprinted genes Cdkn1c and Igf2 interact to promote terminal differentiation of adult NSCs
AUTHORS: Anna Lozano-Urena, Esteban Jimenez-Villaba, Laura Lazaro-Carot, Raquel Montalban-Loro, Irene Martinez-Gurrea, Keiichi I. Nakayama, Martina Kirstein, and Sacri R Ferron

Dear Dr. Ferron,

Thank you for your appeal on your recently rejected manuscript. I do understand your disappointment, but given the opinions expressed by the reviewers, I saw little option other than to decline the paper.

However, I appreciate that in your rebuttal letter, you state that you will be able to address the main concerns of the referees. Therefore I am willing to reconsider a revised version of your manuscript that addresses the points raised by the reviewers. Upon resubmission, please provide a detailed response to the reviewers' comments and highlighting particularly any concerns that have not been included in the revised manuscript.

The revised manuscript and rebuttal will be sent to the original reviewers. If they are convinced by your arguments, then we would be able to consider the manuscript for publication.

To submit a revision, please go to your Author Area and click on the 'Submit a Revision' link.

Yours sincerely,

Francois Guillemot
Handling Editor
Development

First revision

Author response to reviewers' comments

Reviewer 1

In the manuscript presented by Lozano-Ureña et al., the authors investigate the effects of IGF2 on terminal differentiation of adult Neural Stem Cells (NSCs) and its interaction with the imprinted gene *Cdkn1c*. Using differentiating neurospheres based cultures, they show that an addition of IGF2 promotes NSCs terminal differentiation and upregulates *Cdkn1c* gene expression. Investigating the regulation of *Cdkn1c* expression by IGF2, they show that IGF2 action is not linked to a loss of imprinting of *Cdkn1c* but rather to the activation of the PI3K-Akt pathway. Furthermore, they show that inactivation of p57, the cyclin-dependent kinase inhibitor encoded by *Cdkn1c*, alters the effects of IGF2 on NSCs terminal differentiation. In general, the experiments are well designed and controlled, the figures are thorough. The manuscript contains publishable data but the impact of the results is limited. For instance, the link between IGF2 and p57 expression via PI3K-Ekt pathway is not new as it has been already demonstrated in Thomas et al. (2016) in the hematopoietic stem cells like the authors mentioned in the manuscript.

We have performed several *in vivo* experiments that we believe now increase significantly the impact of the results.

First, in order to characterize the function of p57 in the maintenance and differentiation of NSCs also *in vivo*, a flow cytometry analysis of the different cell fractions from the SVZ has been

© 2023. Published by The Company of Biologists under the terms of the Creative Commons Attribution License (https://creativecommons.org/licenses/by/4.0/).

performed in 3 months-old *Cdkn1c-Gfap*^{mat} and *Cdkn1c-Gfap*^{control} mice following a previously described protocol from our group (Belenguer et al., 2021). This protocol distinguishes between GLAST^{+} quiescent NSCs (qNSC) and GLAST/EGFR^{+} activated NSCs (aNSCs), and other populations from the SVZ, such as GLAST^{-}/CD24^{+}/PSA-NCAM^{+} neuroblasts (NB), GLAST^{-}/CD24^{-}/EGFR^{+} neural progenitors (NPCs) and CD9^{+} terminally differentiated astrocytes (Belenguer et al., 2021). Importantly, *Cdkn1c* deletion resulted in reduced numbers of aNSCs that correlated with a reduced number of primary neurospheres obtained from the SVZ of *Cdkn1c-Gfap*^{mat} mice. Consequently, a decrease in the percentage of the NB population was observed in *Cdkn1c* deficient mice. Loss of p57 also resulted in a significant increase in the terminally differentiated astrocytic population in *Cdkn1c-Gfap*^{mat} mice compared to wild-type. Moreover, a similar analysis in 8 months-old mice has been performed and results confirmed the loss of NB production and the induction of astrocyte differentiation in *Cdkn1c-Gfap*^{mat} mice, which were accelerated with age. These data have been added to **Figure 4** and **Supplementary Figure 7** of the new version of the manuscript.

<u>Secondly</u>, to further study the role of p57 in NSC differentiation *in vivo*, a lineage tracing experiment have been performed using brain electroporation in *Cdkn1c-Gfap*^{mat} and *Cdkn1c-Gfap*^{control} mice. A Piggy BAC integrative vector carrying a red fluorescent protein (RFP) together with a transposase under *GLAST* promoter have been used to permanently label with RFP NSCs and their progeny in newborn pups at 3 days of age. This procedure takes advantage of the brain ventricles to allow the introduction of DNA into the lining NSCs and has been extensively used by our group (Fabra-Beser et al., 2021; Mateos-White et al., 2020). Sixty days after electroporation, we have analyzed the number of RFP labeled cells migrating through the RMS and reaching and integrating in the OB in control and p57 deficient mice. Notably, RFP^{+} cells were far less numerous in the RMS of *Cdkn1c-Gfap*^{mat} mice resulting in fewer NBs in the OB. Consistently with the flow cytometry an increased percentage of RFP^{+} cells that expressed the astrocytic marker S100β in the striatum of *Cdkn1c-Gfap*^{mat} mice confirming the role of p57 in the differentiation process of adult NSCs also *in vivo*. These data have been included as **Figure 4** in the new version of the manuscript.

<u>Finally</u>, in order to functionally test whether p57 could also mediate the differentiation effects of IGF2 *in vivo*, we have infused 100 μg/ml^{-1} of recombinant IGF2 or PBS solution for 7 days into the lateral ventricle of *Cdkn1c-Gfap*^{mat} and *Cdkn1c-Gfap*^{control} mice brains. Fifteen days before pump implantation we have injected mice with BrdU and euthanized them immediately after pump removal (see schematic in new **Figure 5A**). In the SVZ, fast-proliferating transit-amplifying progenitors are expected to dilute out the BrdU but it is specifically retained in slowly proliferating NSCs (label-retaining cells, BrdU-LRCs), in olfactory bulb (OB) newborn neurons and in corpus callosum (CC) newborn oligodendrocytes that cease to divide and undergo terminal differentiation soon after the injection (Ferrón et al., 2007; Menn et al., 2006). Notably, the number of BrdU-LRCs in the SVZ was significantly increased after IGF2 infusion in both wild-type and *Cdkn1c* deficient mice and more of them were positive for the proliferation antigen Ki67. This was consistent with a role of IGF2 promoting the proliferation of NSCs in the SVZ as we had previously described (Ferrón et al., 2015; Lehtinen et al., 2011; Ziegler et al., 2014). Importantly, we observed that these proliferative effects were independent of p57, as a similar rate of activation was observed in NSCs within the SVZ of both genotypes after infusion. These data have been added as **Figure 5** in the new version of the manuscript.

Moreover, and consistently with the *in vitro* differentiation study, IGF2 infusion induced a higher proportion of differentiated cells within the adult wild-type SVZ. Immunohistochemistry analysis of terminally differentiated astrocytes (GFAP/S100β^{+}), neuroblasts (DCX^{+}) and oligodendrocytes (OLIG2^{+}) showed a more differentiated state of the niche after IGF2 infusion in control mice. Moreover, when the infusion of IGF2 was performed in *Cdkn1c- Gfap*^{mat} mice, an induced astrocytic fate was observed at expenses of neuronal and oligodendroglial differentiation of NSCs. These results demonstrate that IGF2 promotes proliferation and differentiation of NSCs, but that only differentiation is dependent of p57 balancing the decision between astrocytic and neuronal differentiation *in vivo*. These data have been added as **Figure 6** in the new version of the manuscript.

© 2023. Published by The Company of Biologists under the terms of the Creative Commons Attribution License (https://creativecommons.org/licenses/by/4.0/).

Reviewer 1 Comments for the Author:

The title is somewhat misleading, as epigenetics is not involved in the interaction of *Cdkn1c* and IGF2. For instance, the imprinted nature of IGF2 gene is not implicated in the function described here nor in its interaction with *Cdkn1c*. On the same line, the results do not show a regulation of *Cdkn1c* by IGF2 via a regulation of its methylation.

As the reviewer mentions IGF2 is not regulating the imprinting status of *Cdkn1c* in adult NSCs, thus we agree that the title might be confusing. However, given that we show here that NSCs have the expected maternal expression of *Cdkn1c*, and that this has not been described previously for this type of cells, we find important to keep in the title the imprinted nature of *Cdkn1c*. Thus following reviewer´s suggestion, we propose a new title of the manuscript: "*Igf2 interacts with the imprinted gene Cdkn1c to promote terminal differentiation of NSCs*".

Furthermore, the manuscript exhibits many redundancies between the introduction and the discussion, and ideas are repeated even within the discussion.

These redundancies have been eliminated now and a better discussion of the ideas, including the *in vivo* role of p57 mediating the differentiation effects of IGF2, have been added to the new version of the manuscript.

Specific points:

- **In the title of the second part "IGF2 induces the expression of the maternally expressed gene….", besides the redundancy of wording, the term "maternally" is misleading as the maternally imprinting is not a subject of this part.**

We have now rephrased this title as: *"IGF2 induces the expression of Cdkn1c in differentiating NSCs in vitro"*

- **In the text, figure 4e illustrates the following claim: "the proportion of cells with the capacity to still form neurospheres in non-adherent cultures after differentiation was again reduced after IGF2 treatment in wild- type and Cdkn1c-Gfappat but not in Cdkn1c-Gfap^{mat} cultures (Fig. 4e)." However, the graph shows a significant reduction of the number of spheres obtained from cdkn1c-GFAPmat genotype after IGF2 treatment compared to the untreated condition. This sentence should be changed.**

We apologize for the lack of clarity. We have repeated the experiment and the treatments from old Figure 4e (new **Figure 3E**) in four more cultures. We have performed a new statistical analysis with the combined data using the non-parametric Kruskal-Wallis test comparing different genotypes and Mann Whitney tests comparing the treatment condition. This new analysis indicates that although *Cdkn1c-Gfap*^{mat} cultures form less spheres in the presence of IGF2 than in untreated conditions, this decrease in the number of spheres is significantly higher in control and *Cdkn1c-Gfap*^{pat} IGF2-treated cultures (27% less spheres in *Cdkn1c-Gfap*^{mat} cultures after IGF2 treatment; versus 41% and 54% less spheres in *Cdkn1c-Gfap*^{control} and *Cdkn1c-Gfap*^{pat} cultures, respectively). This confirms that p57 mediates, at least partly, the differentiation effects of IGF2 in NSCs *in vitro*. These new data and the statistical analysis have been added as new **Figure 3E** and the sentence has been rephrased in the new version of the manuscript.

- **For the statistical analysis, the authors used t-test when comparing two groups which is not adequate when the sample is <30. A non-parametric test like Mann-Whitney U test or Wilcoxon Signed Rank test should be used instead.**

We have now analysed data for normality and those cases in which the set of data were not adjusted to normality we have applied non-parametric tests. These new analyses have been included in the new version of the manuscript both in Figures and in the statistical part of methods. See also **Table for Reviewers 1.**

© 2023. Published by The Company of Biologists under the terms of the Creative Commons Attribution License (https://creativecommons.org/licenses/by/4.0/).

- To help the reading, the meaning of the "n" values that are integrated in the figures must be added in the captions.

We have now clearly added the n values to all Figures.

Reviewer 2

This research article investigates the role of IGF2 in differentiation of neurosphere-derived cells from adult SVZ in vitro. The results show that IGF2 increases differentiation of both neuronal and glial cells from neurospheres and that this differentiation is not dependent on genomic imprinting but on p57-dependent signaling. IGF2 conveys its effects on p57 via Akt/Erk kinase pathway. This is an interesting, additive research article that helps to refine the understanding of the role of IGF2 in the biology of adult neural stem cells. The most substantial novelty contribution of the article to the field is not the underlying connection between IGF2 signaling and p57 in differentiation but the finding that unlike cell maintenance roles of IGF2, its function in cell differentiation does not require genomic imprinting. Unfortunately, this most surprising finding is not explored beyond the initial description. While this study is intriguing, it suffers from several major conceptual flaws. However, if these flaws are corrected by a major revision, the publication may have more significant impact on the fields of neurogenesis, genomic imprinting and IGF2 signaling.

Reviewer 2 Comments for the Author:

Major concerns:

1. The entire study is performed on neurospheres or neurosphere-derived cell cultures. While useful for discrete purposes, the neurosphere assay developed by Reynolds and Weiss displays many artifacts and limitations, already recognized in early 2000s (see review by Jensen and Parmar, Mol. Neurobiol. 2006, for example). To ensure that their findings in vitro are relevant, the authors should replicate the IGF2-dependent pro-differentiation effects in vivo, ideally by administration of IGF2 by osmotic mini-pumps to the lateral ventricles of mice or rats.

As the reviewer suggested and in order to functionally test whether p57 could also mediate the differentiation effects of IGF2 *in vivo*, we have infused 100 μg ml^{-1} of recombinant IGF2 or PBS solution for 7 days into the lateral ventricle of *Cdkn1c-Gfap*^{mat} and *Cdkn1c-Gfap*^{control} mice brains. Fifteen days before pump implantation we injected mice with BrdU and euthanized them immediately after pump removal (see schematic in new **Figure 5A**). In the SVZ, fast- proliferating transit-amplifying progenitors are expected to dilute out the BrdU and to be specifically retained in slowly proliferating NSCs (label-retaining cells, BrdU-LRCs), in olfactory bulb (OB) newborn neurons and in corpus callosum (CC) newborn oligodendrocytes that cease to divide and undergo terminal differentiation soon after the injection (Ferrón et al., 2007; Menn et al., 2006). Notably, after IGF2 infusion the number of BrdU-LRCs in the SVZ was significantly increased in both wild-type and *Cdkn1c* deficient mice (see new **Figure 5B-D**) and more of them were positive for the proliferation antigen Ki67 (see new **Figure 5E-G**). This was consistent with a role of IGF2 promoting the proliferation of NSCs in the SVZ as we previously described (Ferrón et al., 2015; Lehtinen et al., 2011; Ziegler et al., 2014). However, we observed that these proliferative effects were independent of p57, as a similar rate of activation was observed in NSCs of both genotypes after infusion (see new **Figure 5C-G**).

Moreover, and consistently with the *in vitro* differentiation study, IGF2 infusion induced a higher proportion of differentiated cells within the adult wild-type SVZ (see new **Figure 6**). Immunohistochemical analysis of terminally differentiated astrocytes (GFAP/S100β^{+} cells), neuroblasts (DCX^{+} cells) and oligodendrocytes (OLIG2^{+} cells) showed a more differentiated state of the niche after IGF2 infusion in control mice (see new **Figure 6A-D**). Importantly, when the infusion of IGF2 was performed in *Cdkn1c-Gfap*^{mat} mice, a more exacerbated induction of the astrocytic fate was observed in the infused mice and this was at expenses of neuronal and oligodendroglial differentiation of NSCs (see new **Figure 6**). This *in vivo* data demonstrates that IGF2 promotes the

© 2023. Published by The Company of Biologists under the terms of the Creative Commons Attribution License (https://creativecommons.org/licenses/by/4.0/).

differentiation of NSCs in a p57 dependent manner balancing the decision between astrocytic or neuronal differentiation. These data have been added as **Figure 6** in the new version of the manuscript.

In addition, experiments on cell differentiation should be replicated on primary adult NSC cultured in monolayer (Costa et al., Development 2011) without previous exposure to EGF/FGF2 (more below). Primary cell cultures suffer from fewer artifacts than neurospheres.

2. Exposure of primary neurospheres to EGF/FGF2 may introduce artifacts and change sensitivity of neurosphere-derived cells to mitogens, including IGF2, which can alter IGF2-dependent effects and interpretation of data. The authors should avoid this pitfall by replicating some of their experiments (such as IGF2-dependent cell pro-differentiation and increased Cdkn1c expression) on neurosphere-derived cells by experiments on primary cell cultures not exposed to EGF/FGF2.

In order to address this important question, we have performed primary cultures from the adult SVZ in the absence of mitogens, using methods previously established (Costa et al., 2011). As the reviewer suggested, treatment with IGF2 has been done in DMEM/F12 supplemented only with B27 in monolayer cultures. Immunocytochemical analysis for p57 has been done in absence or presence of IGF2. Consistent with our previous data in neurosphere cultures, p57 expression was significantly increased in primary cultures after IGF2 treatment (see new **Figure S3D, E**). After 5 days *in vitro*, immunoreactivity of cultures has been performed to determine the presence of neurons (βIII-Tubulin^{+} cells), astrocytes (GFAP^{+} cells) and oligodendrocytes (OLIG2^{+} cells). Treatment of primary cultures with IGF2 also caused a higher proportion of GFAP^{+} astrocytes, of βIII-Tubulin^{+} neurons and of OLIG2^{+} cells (see new **Figure S3F,G**) confirming the role of IGF2 on the acquisition of a differentiated state of NSCs cells from the SVZ. These data have been added to Supplementary Figure 3 in the new version of the manuscript.

3. The entire article stands on the assumption that SVZ neurospheres and neurosphere-derived cells express receptors for IGF2. But this is not directly showed. The authors should show that the cells express IGF2R (or insulin receptor) and how this expression changes with IGF2 administration. Without this evidence, it is hard to accept the results or to know if IGF2 in the used concentration (10x EC50 - see minor points below) activates its receptors or non-specifically activates different targets.

In response to this suggestion, we have conducted the additional qPCR analysis in differentiated cultures in the presence or absence of recombinant IGF2. Insulin receptor (*Insr*), IGF1 receptor (*Igf1r*) and IGF2 receptor (*Igf2r*) have been analysed (see new **Figure S5A**). We have also performed a western-blot analysis for the levels of phosphorylation of IR and IGF1R, as well as total levels of IGFR2. Although the levels of expression of the genes were not significantly elevated after IGF2 treatment, IGF2 induced the phosphorylation of INSR and IGF1R but not IGF2R. These data have been added as **Figure 2A** in the new version of the manuscript.

4. Statistical treatment of data is problematic. The data are analysed by parametric statistical tests, however, it is not indicated if data were not normally distributed or even if normality was tested. In fact, in many cases, the data sets contain only 3-4 biological replicates, which does not allow proper testing of normality even with the Kolmogorov-Smirnov Test. First, the normal distribution of data must be tested where possible (N> 5). Normally distributed data sets can be tested with parametric statistical tests. Where testing of normal distribution of data is not possible or not warranted (N<5), parametric tests must be replaced by non-parametric tests (e.g. Kruskal-Wallis instead of ANOVA, Mann-Whitney instead of T-Test etc.). Applying parametric tests when not appropriate increases false failure of rejecting the null hypothesis (e.g. Type II error).

As suggested by the referee we have increased, when possible, the number of animals or cultures analysed. As for the statistical analysis, data have been firstly tested for normality with Shapiro-Wilk test. The significance of the differences between groups have been evaluated with adequate statistical tests for each comparison. For data that passed normality tests: when analyzing only one variable, t test has been used for comparing two groups (paired t test when possible), and one-way

© 2023. Published by The Company of Biologists under the terms of the Creative Commons Attribution License (https://creativecommons.org/licenses/by/4.0/).

ANOVA followed by Benjamini, Krieger and Yekutieli post-hoc test for three or more groups; when two variables are analyzed, two-way ANOVA followed by Benjamini, Krieger and Yekutieli post- hoc tests have been applied. For data groups that did not pass normality Wilcoxon or Mann-Whitney non-parametric tests have been applied, depending on whether samples were paired or not, respectively. For variables with more than two categories, Kruskal-Wallis followed by Benjamini, Krieger and Yekutieli post-hoc test have been used. For temporal measures that passed normality test, lineal regression analysis has been performed. These analysis and p-values have been included in the new version of the manuscript and figures, and the tests performed in each one of the figure panels have been summarised in **Table 1 for reviewers.**

<u>Minor concerns:</u>

1. It is not clear what the null hypothesis of the article is? This should be stated explicitly.

The study is developed with the goal of identifying mechanisms of regulation of adult NSCs differentiation. The alternative hypothesis of this work is that paracrine factor IGF2 interacts with p57 to balance differentiation of adult NSCs from the SVZ. Based on this, the null hypothesis of this work is that IGF2 do not influence NSC differentiation. We have clearly included the alternative hypothesis in the manuscript.

2. Concentration of IGF2 used in this article is 100 ng/ml. Molecular weight of IGF2 is 632 (or around 20 kDa) and its EC50 to IGF2R is reported to be around 20 nM. 100 ng/ml corresponds to about 160 nM – 10 times more than its EC50. This seems like unnecessarily high concentration. As a proof of principle, the authors should show that even 20 nM IGF2 can induce its differentiation effects to avoid non-specific artifacts.

Previous studies using lower concentrations of IGF2 in differentiation assays of NSCs resulted in a lower percentage of oligodendrocytes after 7 days of differentiation. Whereas a 10.3 ± 0.9% of $O4^+$ cells were obtained in cultures treated with 100 ng ml^{-1} of IGF2, only a 1.5 ± 0.5% of O4+ cells were obtained when IGF2 was added at 10 ng ml^{-1}. Although the percentages of neurons and astrocytes obtained *in vitro* did not change with lower concentrations of IGF2, the oligodendroglial phenotype was reduced, for the reason we decided to maintain the higher dose of IGF2 in the studies. The differentiation studies with 10 ng ml^{-1} of IGF2 are shown in **Figure 1 for Reviewers**.

3. Matrigel used for culturing neurosphere-derived cells might cause small proportion of pro-neuronal differentiation (almost zero in control condition in Fig.1f). This almost zero differentiation to βIII-Tub cells is disconcerting. A solution could be growing cells without Matrigel as primary cell cultures (see major concerns above).

We are happy to clarify this concern. In the manuscript, the differentiation of neurosphere cultures in control conditions was done in the absence of insulin in the culture media, causing a complete abrogation of the insulin pathway. It has been shown that this defective insulin receptor signalling, negatively perturbs neural differentiation and affects neurogenesis during development (Liu et al., 2014; Teo et al., 2021). Moreover, we have previously shown that absence of insulin stimulation in neurosphere cultures causes the aberrant differentiation of neurons *in vitro* (Chidambaram et al., 2022; Chirivella et al., 2017). Thus, the low percentage of neurons in untreated conditions was due to the absence of insulin activation in the culture media. However, to determine whether matrigel can somehow impair the neuronal differentiation capacity of NSCs we have performed neurosphere cultures differentiation for 7 DIV on poly-D-lysine and laminin. This study revealed that neurosphere cells from the adult SVZ only attach and differentiate efficiently on Matrigel. These results are shown in **Figure 2 for reviewers**.

4. Given the extremely low expression of Tuj1 in Fig.1d raises a question of how specific the RT-qPCR primers were. Was the primer specificity tested?

Primers used for qPCR experiments were Taqman probes obtained from Applied Biosystems. More concretely the catalogue number for the *βIII-tubulin* probe is Mm00727586_s1. These primers have been previously used by our lab (Belenguer et al., 2016; Montalbán-Loro et al., 2019) and by other

© 2023. Published by The Company of Biologists under the terms of the Creative Commons Attribution License (https://creativecommons.org/licenses/by/4.0/).

groups (Logan et al., 2021; Miura et al., 2019) and is the best coverage one recommended for the company (see **Figure 3 for reviewers**, panel A).

5. What was the cut-off for delta Ct values for qPCR? If it was above 35, such expression data suffer from artifacts (e.g. primer-dimer driven amplification) and lack biological meaning.

The cut-off for delta Ct values for *βIII-tubulin* was around 30 cycles as indicated in **Figure 3 for reviewers**, panels B and C, thus we believe that no artifacts occurred in the qPCR amplifications.

6. Were qPCR data analyzed as delta-delta Ct as described by Livak and Schmittgen, Methods 2001? What was the housekeeping gene the expression was compared to?

In the original manuscript, the data were analysed as Delta-Ct to determine the absolute quantification of the different genes. Following reviewer's indications, we have now analysed qPCR expression data as Delta-Delta Ct. These analyses are now shown as new **Supplementary Figure 2A**. The housekeeping gene used for the analysis was *Gapdh* (Applied Biosystems Mm99999915_g1) as indicated in **Figure 3 for reviewers**.

7. In support to the major concern 2, there is the result, which shows that withdrawal of EGF increases *Cdkn1c* expression, suggesting that EGF artificially suppresses *Cdkn1c* expression. This result calls for replicating the Cdkn1c expression experiment in the absence of EGF and in primary cell cultures.

As mentioned, we have used now methods previously established (Costa et al., 2011) to grow primary cultures from the SVZ. New treatments with IGF2 have been done and levels of p57 protein have been quantified from these cultures. A clear increase in the protein levels of p57 was observed in IGF2 treated cultures. These data have been added as **Supplementary Figure 3D** to **3G**.

8. What cell identify the control neurosphere-derived cells in Fig.1 had? Since there were no bIIItubulin+ and almost no O4+, S100b+ and GFAP+, what were the control cells? One would expect more GFAP+ NSC-like cells but Fig.1e shows very few of these as well. The authors should quantify number of GFAP+/Nestin+ (or Vimentin+) NSCs in their cultures.

We apologize for the lack of clarity both in the text and in the data and images provided.

As we mentioned in concern 3 of this reviewer, it is well known that NSCs needs the activation of the insulin pathway to differentiate into neurons, astrocytes and oligodendrocytes (Chidambaram et al., 2022; Chirivella et al., 2017). Therefore, to determine the specific role of IGF2 in the differentiation process of NSCs, our control neurosphere culture system (untreated) was performed in insulin-free medium corresponding with starving conditions. Results in Figure 1f of the original manuscript showed a high percentage of undifferentiated cells in these starving conditions caused by the absence of any insulin pathway stimulation in the culture media. Moreover, as the reviewer suggested, we have quantified the number of Nestin^{+} cells in our cultures, and corroborated that untreated cells were unable to properly acquire a differentiated phenotype. Consistently IGF2 treatment of cultures reduced the number of Nestin^{+} cells *in vitro*. This analysis has been added as **Supplementary Figure 2B** in the new version of the manuscript.

9. While the summary results in Fig.2c show a very small increase of p57 protein levels in cells treated with IGF2, the representative blots are not convincing. With N=4, I am concerned that this increase is an artifact of inappropriately used parametric statistical tests. The rather small increase in p57 protein levels does not correspond to quite robust increase in Cdkn1c mRNA in Fig.2a. All data should be re-tested with non- parametric tests where appropriate, but it is especially important for data in Fig.2c.

We have now performed two more experiments in NSCs after 2 DIV of differentiation and have included the new analysis to the previous data (6 experiments for 2 DIV in total). We have used the parametric t test (paired data) after the confirmation that the data passed the Shapiro-Wilk normality test. A significant increase in the p57 protein levels has been confirmed in IGF2 treated cultures. New blots and the quantification graph for p57 levels, have been added as **Figure 1F** in

© 2023. Published by The Company of Biologists under the terms of the Creative Commons Attribution License (https://creativecommons.org/licenses/by/4.0/).

the new version of the manuscript. Complete blots are shown in new **Supplementary Figure 9**.

10. Third paragraph in the introduction states "The potential role of p57 in the regulation of neurogenesis in the adult SVZ niche has not been addressed". This is not true. p57 has been implicated in NSC proliferation in the SVZ in the context of ischaemia and repetitive magnetic stimulation (Guo et al., PLoS ONE 2014). This should be corrected and this article should be cited.

We have now included this reference and have rephrased this statement in the introduction of the new version of the manuscript.

11. Given the regulation of p57 expression by miR-25, and co-current regulation of p57 and PTEN, it would be useful to see if IGF2 administration changes expression of both miR-25 and PTEN.

We have now performed a qPCR study for *Mir25* and *Pten* genes in cultures grown in the presence or absence of IGF2. As shown in **Figure for reviewers 4**, no changes were observed in *Mir25* or *Pten* expression in NSCs after IGF2 treatment either in proliferation or after 2 and 3 DIV of differentiation. These data can be added to the manuscript.

12. When showing results from cells in differentiating conditions, it is not clear if all these experiments followed the protocol as described in Fig.1a.This should be made clear.

We have now indicated in each figure the time of differentiation condition.

13. In figure 3, there is a missing control of cells treated with Akt and/or Erk inhibitors but in the absence of IGF2 administration. Alternatively, stimulation of phosphorylation of Akt/Erk should replicate IGF2 effects on differentiation.

We have performed a new experiment in NSCs after 2 DIV of differentiation in which we have included the untreated condition with either Akt or Erk inhibitors. No differences with untreated cells and these conditions were observed in the new experiments. These studies are shown in **Figure 2C** in the new version of the manuscript.

14. How do authors explain the discrepancy between their previous work (Ferron et al., Nat. Comm. 2015), which shows that genomic imprinting is essential for IGF2-dependent cell maintenance of adult NSCs from SVZ, yet their results in this study (Fig.4) show that IGF2 role in cell differentiation in vitro is not dependent on genomic imprinting?

Our previous work described that IGF2 acts as a paracrine factor secreted by the vascular compartment and choroid plexus regulating the function of NSCs in the adult SVZ neurogenic niche (Ferrón et al., 2015). Moreover, as the reviewer mentions, we found that absence of genomic imprinting of the *Igf2* gene caused its biallelic expression in these compartments. However, and given that IGF2 acts *in vivo* as a secreted factor and not in an autocrine manner, in this new study we have mimicked the action of the factor by exogenously adding recombinant IGF2 to the culture medium or to the lateral ventricles *in vivo*. These strategies increase the levels of the factor in the niche or in the culture but without altering its physiological biallelic expression of IGF2 in the brain. Furthermore, Figure 4 in the original manuscript addressed the imprinting status of the *Cdkn1c* but not of the *Igf2* gene. We have clarified this point in the discussion of the new version of the manuscript.

Reviewer 3

The role of the imprinted gene Cdkn1c/p57 in the regulation of neurogenesis in the adult Sub Ventricular zone (SVZ) niche is unknown. Using a number of <u>elegant experiments the authors conclude that p57 acts</u> <u>downstream of IGF2 signalling to control the differentiation programme of adult neural stem cells</u>.

© 2023. Published by The Company of Biologists under the terms of the Creative Commons Attribution License (https://creativecommons.org/licenses/by/4.0/).

Reviewer 3 Comments for the Author:

The paper is well written and presented. There are a number of issues that I feel will be important to address prior to potential publication:

Major points:

1. The conclusion that IGF2 downstream signalling effects on Cdkn1c expression are not mediated by ERK1/2 is entirely based on the expression of Cdkn1c not being reduced at mRNA level after exposure to MEK/ERK inhibitor PD0325901. This is in spite of ERK1/2 increased activity upon IGF2 stimulation, measured by phosphorylation (shown in 2 c; left panel. The data presented for this inhibitor (shown in figure 3d) are highly variable. Given these issues, it is important to add a 'positive' control that shows that inhibition of ERK1/2 by PD0325901 at the dose applied is in fact occurring in these cells.

We have performed a new experiment in NSCs after 2 DIV of differentiation in which we have included an untreated condition with either Akt or Erk inhibitors. No differences with untreated cells and these conditions were observed in the new experiments. These studies have been included in new **Figure 2C**. Furthermore, to confirm the action of MAPK and Akt inhibitors in NSCs, we have performed immunocytochemistry for phospho MAPK (pMAPK) and phospho Akt (pAkt) in NSCs after treatment with PD0325901 or LY294002 for 2 DIV. A reduction of the levels of pMAPK and of pAkt were observed in NSCs after treatment, confirming that the inhibition of ERK1/2 and Akt was occurring in NSCs. Some images of these stainings are now shown in **Supplementary Figure 5B**. The antibodies used for the staining were: Phospho-p44/42 MAPK Erk1/2 Thr202/Tyr204 (Cell Signaling catalogue number 9101) and pAkt Ser 473 (Cell Signaling catalogue number 9271). These antibodies have been added to the list of primary antibody used in **Table S2**.

Using *Igf2* mRNA levels as the only readout is not adequate and for completeness, the authors should also consider adding differentiation data on IGF2+PD0325901 to Figures 3 e) and f).

We have now performed a differentiation experiment treating NSCs with IGF2 and with the MAPK inhibitor PD0325901. After 7 DIV we have analysed the percentage of neurons (βIII-tubulin^{+} cells), astrocytes (GFAP/S100β^{+} cells) and oligodendrocytes (O4^{+} cells) formed *in vitro*. Notably, no differences in the percentage of differentiated cells were observed after the combined treatment of IGF2 with the MAPK inhibitor, suggesting that the differentiation effects of IGF2 were not mediated by MAPK activation. These new data have been included in **Figure 2D** and **2E**.

The authors should also quantify the total levels of AKT and MAPK against respective loading controls and add this information as supplementary information, with corresponding statistical analysis. It seems to me, by visual inspection of the western blots. that both total AKT and MAPK levels are reduced upon IGF2 treatment. This is important as an increase in activity via phosphorylation may not be of significance if the total levels of these proteins are reduced.

We have quantified total Akt and total MAPK protein levels relative to GAPDH in untreated and IGF2-treated cultures after 2 days in differentiation conditions and found a significant decrease of tAkt and tMAPK after IGF2 treatment. These data are shown in **Figure 5 for reviewers**. We still believe that experiments shown in Figure 2D showing the differentiation effects of IGF2 after inhibition of Akt pathways, indicates that the phosphorylation of Akt is of biological relevance during NSCs differentiation.

2. What are the levels of expression of p57 protein in the 'untreated' conditions during the time course of the experiment?

We have now performed a more detailed study of the levels of expression of p57 during the differentiation process. Immunocytochemistry for p57 in NSCs after 2, 3 and 7 DIV of differentiation have shown that p57 increases significantly at 2 DIV of differentiation and its levels is maintained after 3 and 7 DIV of differentiation. Some examples of this staining are shown in new **Figure 1D** and the quantification of the levels of expression in new **Figure 1E**.

© 2023. Published by The Company of Biologists under the terms of the Creative Commons Attribution License (https://creativecommons.org/licenses/by/4.0/).

Concerning this point There is a mention to 'mitogen withdrawal inducing a 4 fold mRNA increase in Cdkn1c as measured at 2, 3, 7 DIV compared to proliferation conditions (Fig. 2a)' but at 2 DIV it looks more like a 2 fold increase (please add an extra reference point in Y axis) and at 3 and 7 DIV there is hardly Cdkn1c mRNA expression?

As the reviewer suggested we have added an extra reference point in Y-axis to new **Figure 1C** and we have corrected this in the text.

Question remains on levels of p57 protein are at 3 DIV and 7 DIV in untreated conditions and how they relate to mRNA expression in Fig 2a. I would like to suggest to add a panel of p57 protein measurements for untreated conditions (mostly composed by undifferentiated cells) to Figure 2b for time points 2DIV, 3 DIV and 7 DIV (although for 2 DIV they are already shown in Fig 2d). Alternatively, this data on untreated conditions could be added as an extra-plot in Supplementary figure 1b?

Following reviewer´s suggestion, we have added a panel of images in new **Figure 1D**, showing the levels of p57 protein in NSCs after 2, 3 and 7 DIV both in untreated and in IGF2-treated cultures.

3. Related to the point above, the increase of Cdkn1c in untreated conditions at 2 DIV, albeit small, occurs without IGF2 treatment, which raises the question of possible IGF2 independent actions of Cdkn1c. It would be beneficial to add a section in the discussion on the limitations of the study and future directions.

This suggestion is relevant; thus, we have added a sentence in the discussion addressing this point.

For example, the experimental strategy followed here somewhat limits the investigation of Cdkn1c to IGF2- dependent actions, and as such potentially important IGF2-independent roles are not addressed. These could be presumably addressed in other models of differentiation that are not related to exogenous levels of IGF2. In that regard transient transfections of Cdkn1c could be of value to define any IGF2 independent roles?

It has been previously shown that p57 overexpression at embryonic day E14.5 elicits neural precursor cell cycle exit and promotes transition from proliferation to neuronal differentiation (Tury et al., 2011). Therefore, in response to reviewer´s suggestion, we have performed overexpression experiments in adult NSCs followed by the induction of the differentiation of overexpressing cells *in vitro*. We have studied by immunostaining of cultures, proliferating cells and the population of Neurons (βIII-tubulin$^+$ cells), astrocytes (GFAP$^+$ cells) and oligodendrocytes (OLIG2$^+$ cells) formed after 7 DIV of differentiation. This study has revealed that upregulation of p57 causes the cell cycle exit of NSCs followed by their differentiation into the three neural lineages. A decrease in the percentage of Ki67$^+$ cells was observed in p57 upregulated cultures that showed also a more differentiated phenotype than control cultures. These data have been included in **Figure 6 for reviewers**.

There is a degree of repetition between Introduction and Discussion, so discussing the results more broadly would be beneficial.

This has been now corrected in the new version of the manuscript.

Minor points:
1. It is clear from the knockout study (Figs 4d,e) that the actions of IGF2 are only partially mediated by Cdkn1c and as such this should be made clear in abstract and throughout.

This has been corrected in the new version of the manuscript

2. Further experiments looking specifically at cell cycle stages would be required to substantiate the claim, mentioned in abstract for example, that the increase in IGF2 signalling and Cdkn1c lead to cell cycle exit. Although this is indeed very likely, it was not formally shown. Caution should be applied therefore, and advisable to tone down that conclusion by stating that the current data suggests that this might be the case.

© 2023. Published by The Company of Biologists under the terms of the Creative Commons Attribution License (https://creativecommons.org/licenses/by/4.0/).

Our *in vitro* data showed an increase in the percentage of Ki67/Nestin$^+$ cells in IGF2-treated cultures after 2 DIV of differentiation (Figure 1C from old version of the manuscript). However, to further determine the dynamics of proliferation during the differentiation process, additional, studies have been performed in wild-type cultures. Immunostaining studies for the cell cycle markers Ki67 and MCM2 have shown that IGF2 treatment causes an increase in the percentage of proliferating cells in the cultures after 2 and 3 DIV of differentiation. These effects were abrogated after 7 DIV in culture, when differentiation was completed. These data have been included as Supplementary Figure 1C and 1D in the new version of the manuscript.

3. The authors found that the increase of *Cdkn1c* mRNA expression is derived from the active maternal allele, i.e. does not involve loss of imprinting. As such, stating that they have identified a methylation independent mechanism of control of Cdkn1c expression is inadequate.

We apologize for the lack of clarity. We have now removed this statement from the manuscript.

4. Please add details about the specificity of the Cre line- i.e. where is it expressed? - and add the potential limitations of the Cre line if any.

Mice expressing Cre-recombinase directed by the mouse glial fibrillary acidic protein (*Gfap*) promoter are from Jackson Lab (B6.Cg-Tg(Gfap-cre)73.12Mvs/J; Stock No. 012886) (Garcia et al., 2004). The company reports that in contrast to other GFAP-cre lines (for example 77.6 line Stock 024098), these mice have Cre-recombinase activity in essentially all adult NSCs (and their progeny) from the subgranular zone (SGZ) and the subventricular zone (SVZ) neurogenic niches.

However, in order to validate the specificity of Cre-recombinase expression, we have performed an X-gal histochemistry in the adult brains of Gfap-cre mice crossed with a reporter mouse, which carry the β-galactosidase gene under the regulation of the ubiquitous ROSA26 promoter containing a loxP-flanked stop sequence (*Gfap- cre/LACZ*). These mice express the β-galactosidase gene after Cre-mediated recombination and show positive staining for X-gal (substrate for β-galactosidase) in very specific areas of the brain in *Gfap-cre/LACZ* mice. Our study shows a high expression for X-gal in the adult SVZ. Control mice were generated by crossing mice without Cre- recombinase with ROSA26R mice (*Gfap-control/LACZ*). As expected, absence of X-gal staining was observed in these control mice. This study has been added as **Supplementary Figure 6B** to the new version of the manuscript.

5. Related to the Cre line it is stated in Methods that 'in these mice Gfap expression is prevented by the removal of a small region in exon 1' - what does that mean? Please add further information.

This Gfap-Cre line has been previously described in Garcia *et al.*, 2004. To generate these mice authors used a 15- kb promoter cassette containing the full sequence of the mouse *Gfap* gene. This cassette contained all introns, promoter regulatory elements and 2 kb of 3´and 2.5 kb of 5´flanking regions. Therefore, to avoid the expression of the exogenous *Gfap*, a small fragment of the first exon of the *Gfap* gene was removed. We have included these technical details to the Methods section in the new version of the manuscript.

6. The authors should consider showing individual points in all graphs when N<10.

We appreciate reviewer's suggestion. We have now showed individual points in all graphs from *in vitro* and *in vivo* studies except in qPCR expression analysis in which we have decided to use *Box and Whiskers* graphs, to better display variation in the different sets of data.

7. Please specify the precise numbers used in all experiments - e.g. stating N>4; N>=6 is not ideal. The full disclosure of numbers can be done in figures, figure legends and/or in a dedicated supplementary table but should not be ambiguous. Figure 2c and 3C indicates n=4 in graph but only 3 blots are shown in supplementary 5 - therefore, is it n=4 or n=3?

We have specified the number of animals or samples used in all graphs. As for the WB analysis, we show now five different examples for the p57 levels of protein in new **Supplementary Figure 9**.

© 2023. Published by The Company of Biologists under the terms of the Creative Commons Attribution License (https://creativecommons.org/licenses/by/4.0/).

Moreover, as the reviewer suggests, we have also corrected *"n"* in **Figure 2B** from the new version of the manuscript.

8. Please clarify what is meant by normalized counts in Fig 2D - normalised to what precisely? and what does counts mean? please state the number of cells that were analysed in both groups (given that there are obvious differences in cell proliferation).

We apologise for the lack of clarity. The "Y" axis indicates number of cells analysed, thus we have modified this in the graph.

9. Supplementary Figure 4 could actually be added as a Main Figure as it is a nice summary/model of the work.

The summary Figure has been added to the new version of the manuscript as **Figure 7**.

10. Please add the references to the phospho AKT and MAPK antibodies to table S4 (I can only see total ones mentioned).

To detect phospho-Akt and phospho-MAPK by immunoblot, we did not use single antibodies. Instead, we used the antibody PathScan Multiplex WB cocktail I from Cell Signaling made in Rabbit catalogue number 5301, which is a cocktail of antibodies that can be used to assay the activation of multiple pathways and simultaneously detects levels of phospho-p90RSK, phospho-Akt, phospho-p44/42 MAPK (Erk1/2) and phospho-S6 ribosomal protein. The cocktail also includes the Rab11 antibody as a control for protein loading. This antibody is included in new **Supplementary Table 2**.

11. Please specify the genetic background of the non-transgenic mice used in the study

IGF2 treatment or infusion experiments shown in new Figures 1 to 6 were performed in mice from the genetic background C57BL/6. The imprinting study, shown in new **Supplementary Figure 4,** was performed in NSCs derived from adult F1 hybrid offspring obtained from reciprocal crossed between *Mus musculus domesticus* (C57BL/6) and *Mus musculus castaneus* (CAST/EiJ) mice in which we had previously identified a SNP between the two subspecies at the *Cdkn1c* gene. This is now better described in methods section "Expression studies and SNP sequencing".

12. There are a few typos - e.g. IFG2 instead of IGF2; differentaton instead of differentiation.

These typos have been amended in the new version of the manuscript.

References

Belenguer, G., Domingo-Muelas, A., Ferrón, S. R., Morante-Redolat, J. M. and Fariñas, I. (2016). Isolation, culture and analysis of adult subependymal neural stem cells. *Differentiation* 91, 28–41.

Belenguer, G., Duart-Abadia, P., Jordán-Pla, A., Domingo-Muelas, A., Blasco-Chamarro, L., Ferrón, S. R., Morante- Redolat, J. M. and Fariñas, I. (2021). Adult Neural Stem Cells Are Alerted by Systemic Inflammation through TNF-α Receptor Signaling. *Cell Stem Cell* 28, 285-299.e9.

Chidambaram, S., Velloso, F. J., Rothbard, D. E., Deshpande, K., Cajuste, Y., Snyder, K. M., Fajardo, E., Fiser, A., Tapinos, N., Levison, S. W., et al. (2022). Subventricular zone adult mouse neural stem cells require insulin receptor for self-renewal. *Stem Cell Rep* 17, 1411–1427.

Chirivella, L., Kirstein, M., Ferron, Domingo-Muelas, Durupt, Acosta-Umanzor, Cano-Jaimez, Perez-Sanchez, Barbacid, Ortega, S., et al. (2017). Cyclin-Dependent Kinase 4 Regulates Adult Neural Stem Cell Proliferation and Differentiation in Response to Insulin. *Stem Cells* 35, 2403–2416.

Costa, M. R., Ortega, F., Brill, M. S., Beckervordersandforth, R., Petrone, C., Schroeder, T., Götz, M. and Berninger, B. (2011). Continuous live imaging of adult neural stem cell division and lineage progression in vitro. *Development* 138, 1057–1068.

Fabra-Beser, J., Araujo, J. A. M. de, Marques-Coelho, D., Goff, L. A., Costa, M. R., Müller, U. and Gil-Sanz, C. (2021). Differential Expression Levels of Sox9 in Early Neocortical Radial Glial Cells

© 2023. Published by The Company of Biologists under the terms of the Creative Commons Attribution License (https://creativecommons.org/licenses/by/4.0/).

Regulate the Decision between Stem Cell Maintenance and Differentiation. *J Neurosci* 41, 6969–6986.

Ferrón, S. R., Andreu-Agulló, C., Mira, H., Sánchez, P., Marqués-Torrejón, M. Á. and Fariñas, I. (2007). A combined ex/in vivo assay to detect effects of exogenously added factors in neural stem cells. *Nat Protoc* 2, 849–859.

Ferrón, S. R., Radford, E. J., Domingo-Muelas, A., Kleine, I., Ramme, A., Gray, D., Sandovici, I., Constancia, M., Ward, A., Menheniott, T. R., et al. (2015). Differential genomic imprinting regulates paracrine and autocrine roles of IGF2 in mouse adult neurogenesis. *Nat Commun* 6, 8265.

Garcia, A. D. R., Doan, N. B., Imura, T., Bush, T. G. and Sofroniew, M. V. (2004). GFAP-expressing progenitors are the principal source of constitutive neurogenesis in adult mouse forebrain. *Nat Neurosci* 7, 1233–1241.

Lehtinen, M. K., Zappaterra, M. W., Chen, X., Yang, Y. J., Hill, A. D., Lun, M., Maynard, T., Gonzalez, D., Kim, S., Ye, P., et al. (2011). The Cerebrospinal Fluid Provides a Proliferative Niche for Neural Progenitor Cells. *Neuron* 69, 893–905.

Liu, J., Spéder, P. and Brand, A. H. (2014). Control of brain development and homeostasis by local and systemic insulin signalling. *Diabetes Obes Metabolism* 16, 16–20.

Logan, T., Simon, M. J., Rana, A., Cherf, G. M., Srivastava, A., Davis, S. S., Low, R. L. Y., Chiu, C.-L., Fang, M., Huang, F., et al. (2021). Rescue of a lysosomal storage disorder caused by Grn loss of function with a brain penetrant progranulin biologic. *Cell* 184, 4651-4668.e25.

Mateos-White, I., Fabra-Beser, J., Agustín-Durán, D. de and Gil-Sanz, C. (2020). Double In Utero Electroporation to Target Temporally and Spatially Separated Cell Populations. *J Vis Exp*.

Menn, B., Garcia-Verdugo, J. M., Yaschine, C., Gonzalez-Perez, O., Rowitch, D. and Alvarez-Buylla, A. (2006). Origin of Oligodendrocytes in the Subventricular Zone of the Adult Brain. *J Neurosci* 26, 7907–7918.

Miura, K., Harikae, K., Nakaguchi, M., Imaimatsu, K., Hiramatsu, R., Tomita, A., Hirate, Y., Kanai-Azuma, M., Kurohmaru, M., Ogura, A., et al. (2019). Molecular and genetic characterization of partial masculinization in embryonic ovaries grafted into male nude mice. *Plos One* 14, e0212367.

Montalbán-Loro, R., Lozano-Ureña, A., Ito, M., Krueger, C., Reik, W., Ferguson-Smith, A. C. and Ferrón, S. R. (2019). TET3 prevents terminal differentiation of adult NSCs by a non-catalytic action at Snrpn. *Nat Commun* 10, 1726.

Teo, A. K. K., Nguyen, L., Gupta, M. K., Lau, H. H., Loo, L. S. W., Jackson, N., Lim, C. S., Mallard, W., Gritsenko, M. A., Rinn, J. L., et al. (2021). Defective insulin receptor signaling in hPSCs skews pluripotency and negatively perturbs neural differentiation. *J Biol Chem* 296, 100495.

Tury, A., Mairet-Coello, G. and DiCicco-Bloom, E. (2011). The Cyclin-Dependent Kinase Inhibitor p57Kip2 Regulates Cell Cycle Exit, Differentiation, and Migration of Embryonic Cerebral Cortical Precursors. *Cereb Cortex* 21, 1840– 1856.

Ziegler, A. N., Chidambaram, S., Forbes, B. E., Wood, T. L. and Levison, S. W. (2014). Insulin-like Growth Factor-II (IGF-II) and IGF-II Analogs with Enhanced Insulin Receptor-a Binding Affinity Promote Neural Stem Cell Expansion*. *J Biol Chem* 289, 4626–4633.

© 2023. Published by The Company of Biologists under the terms of the Creative Commons Attribution License (https://creativecommons.org/licenses/by/4.0/).

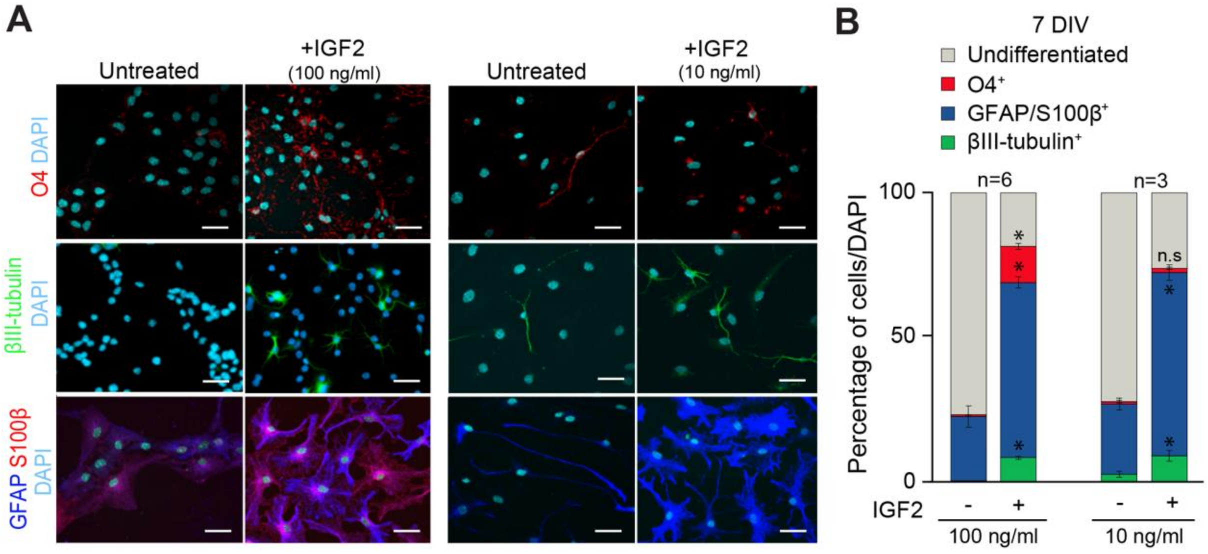

Figure 1 for reviewers. Differentiation of adult NSCs with a lower concentration of IGF2 (10 ng/ml) results in a defective differentiation of NSCs into oligodendrocytes. (A) Immunocytochemistry for O4 (red), βIII-tubulin (green) and GFAP/S100β (blue/red) in NSCs after 7 DIV of differentiation in the absense or presence of IGF2. Two concentration of IGF2 were used to differentiate the cells (10 ng/ml and 100 ng/ml). **(B)** Percentage of oligodendrocytes (O4+ cells), astrocytes (GFAP+ cells) and neurons (βIII-tubulin+ cells) formed in presence of 10 ng/ml and 100 ng/ml of IGF2. Data for untreated cultures are also included. All error bars show s.e.m. of 6 cultures for 100 ng/ml of IGF2 and 3 cultures for 10 ng/ml of IGF2 condition. P-values and number of samples are indicated. A paired t-test (untreated vs. +IGF2) has been applied. Scale bars in A: 30 µm.

© 2023. Published by The Company of Biologists under the terms of the Creative Commons Attribution License (https://creativecommons.org/licenses/by/4.0/).

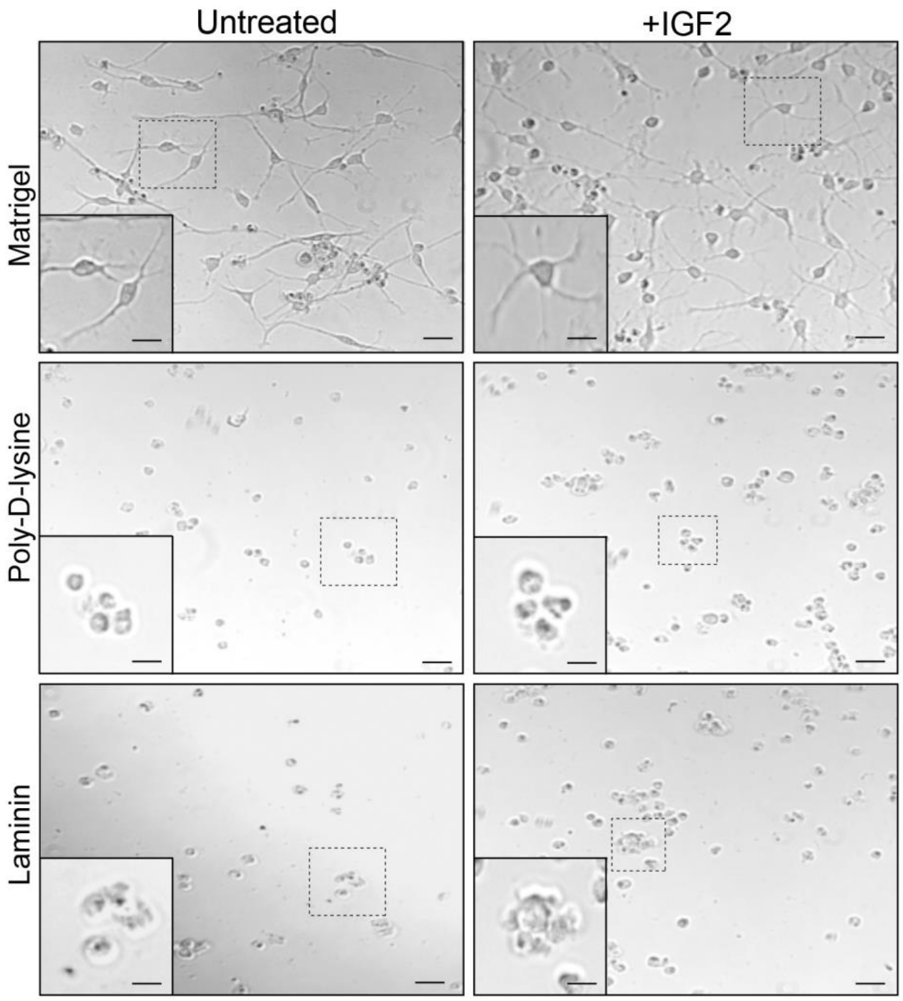

Figure 2 for reviewers. Differentiation of NSCs is more efficient in matrigel-treated plates. Phase contrast images of neurosphere cultures after 7 DIV of differentiation on plates treated with Matrigel (1x), Poly-D-lysine (100 μg/ml) or Laminin (10 μg/ml) and in presence or absence of IGF2. High magnifica- tion images are included. Scale bars: 30 μM (7 μm in inserts).

© 2023. Published by The Company of Biologists under the terms of the Creative Commons Attribution License (https://creativecommons.org/licenses/by/4.0/).

A

Gene	Chromosome	Gene Name	Applied Code	Amplicon size (bp)	Dye
Gapdh	Chr. 6	*glyceraldehyde-3-phosphate dehydrogenase*	Mm99999915_g1	109	FAM
Tubb3	Chr. 8	*tubulin, beta 3 class III*	Mm00727586_s1	64	FAM

B

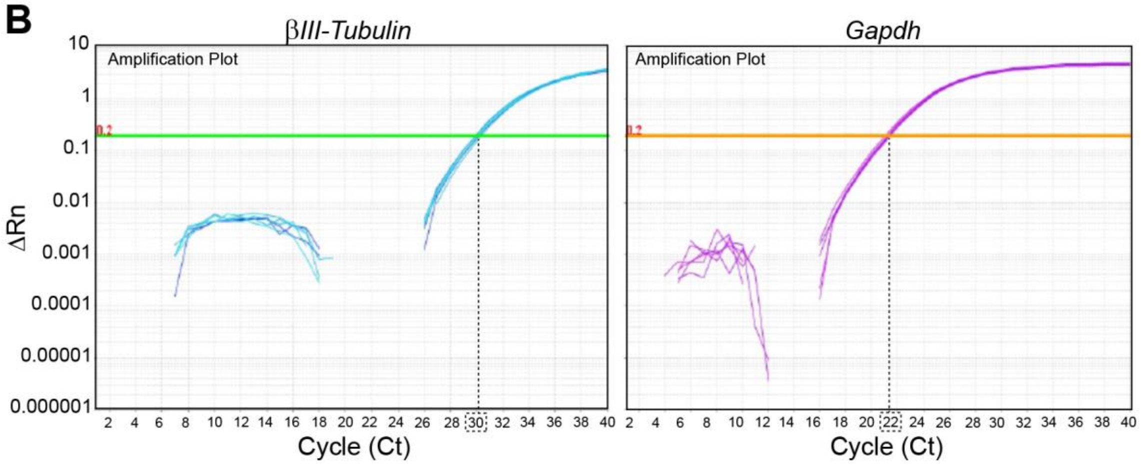

C

		Cycle (Ct)				MEAN			
		GAPDH		***βIII-Tubulin***					
		Rep1	Rep2	Rep1	Rep2	GAPDH	β-III Tub	ΔCt	2^-ΔCt
**-IGF2**	***Culture 1***	19,64	19,76	31,61	31,57	19,70	31,59	11,89	0,00026
	Culture 2	19,51	19,58	31,96	32,09	19,54	32,03	12,48	0,00017
	Culture 3	19,57	19,78	31,57	31,72	19,67	31,65	11,98	0,00025
	Culture 4	19,44	19,66	31,99	31,61	19,55	31,80	12,25	0,00021
**+IGF2**	***Culture 1***	19,84	20,10	31,25	30,75	19,97	31,00	11,03	0,00048
	Culture 2	19,96	19,81	31,46	31,49	19,89	31,47	11,59	0,00032
	Culture 3	19,66	19,98	31,40	31,59	19,82	31,50	11,67	0,00031
	Culture 4	19,71	19,87	31,33	31,01	19,79	31,17	11,38	0,00038
	Rep: technical replicates							**Untreated**	**0,00022**
								**IGF2**	**0,00037**

Figure 3 for reviewers. Analysis of the specificity of *βIII-tubulin* primers. (A) Taqman probes from Applied Biosystem used for quantitative PCR (qPCR) analysis. Name of the gene, chromosome location, amplicon size and company code are indicated. **(B)** qPCR amplification curves for *βIII-tubulin* primers showing different samples. Amplification curves for *Gapdh* (used as housekeeping gene), are also shown. Number of Cycle (Ct) used for the analysis is indicated with dashed lines. **(C)** Table showing amplification cycles or Ct in IGF2-treated experiments. Two technical replicates were done per sample.

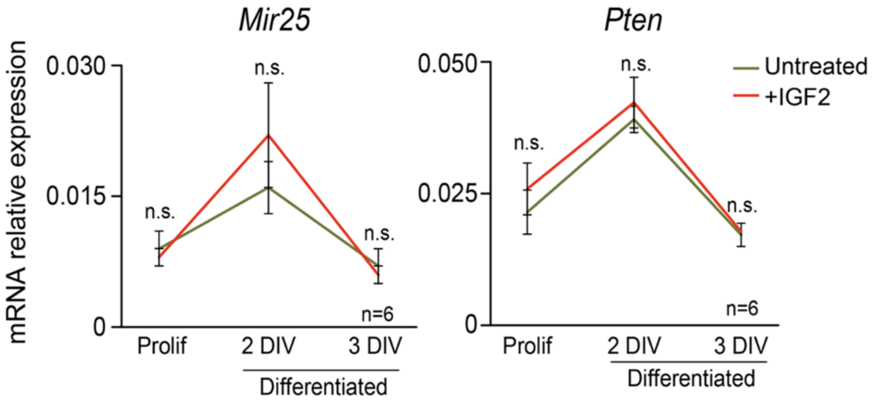

Figure 4 for reviewers. *Mir25* and *Pten* are not altered in NSCs cultu- res treated with IGF2. qPCR for *Mir25* and *Pten* in NSCs cultures in proli- feration (Prolif) conditions and after 2 DIV and 3 DIV of differentiation. All error bars show s.e.m of 6 independent cultures.

© 2023. Published by The Company of Biologists under the terms of the Creative Commons Attribution License (https://creativecommons.org/licenses/by/4.0/).

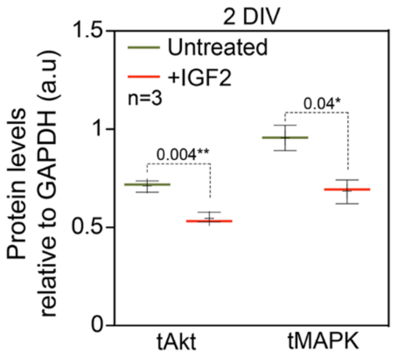

Figure 5 for reviewers. Levels of expression of total Akt and total MAPK. Quantification of total Akt and total MAPK protein levels by western-blot. Data are presented relative to GAPDH in untreated (green) and IGF2-treated (red) cultures after 2 DIV in differentiation conditions. All error bars shows s.e.m of three cultures per conditions. P-values and number of samples are indicated.

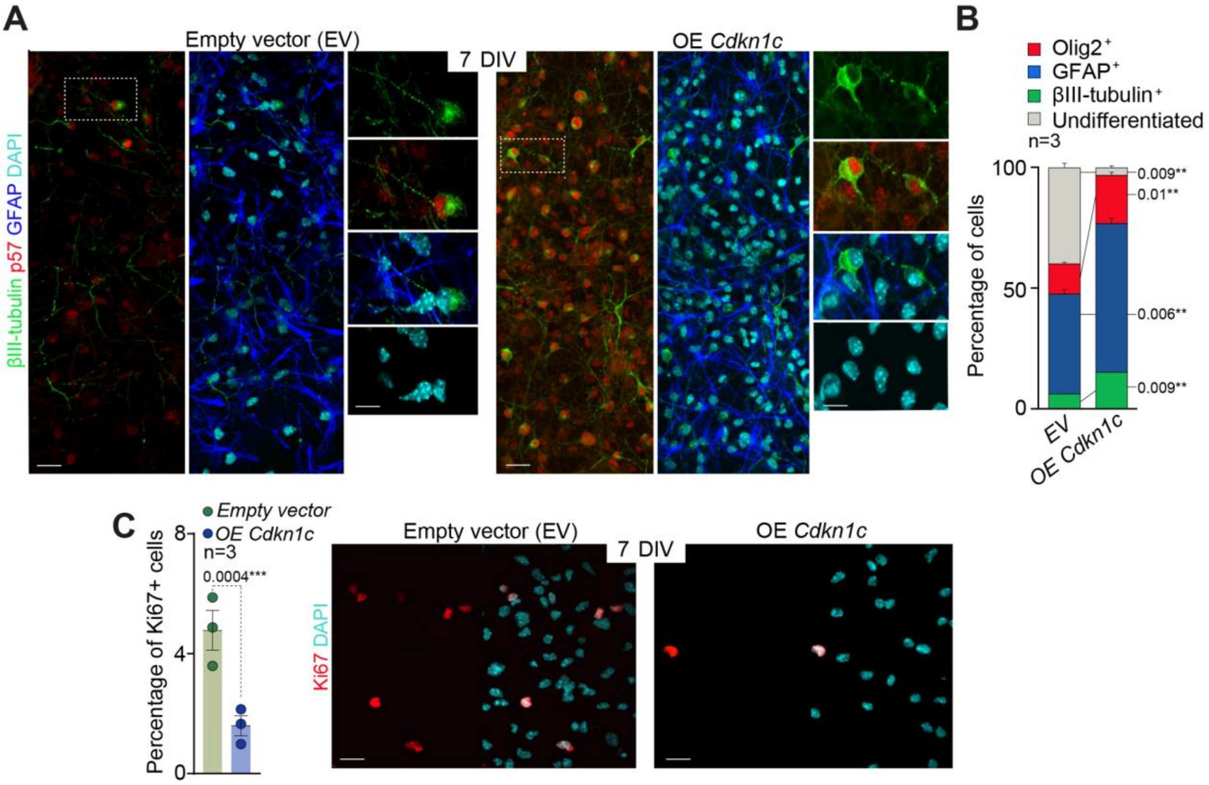

Figure 6 for reviewers. Upregulation of *Cdkn1* promotes differentiation of NSCs into astrocytes, neurons and oligo- dendrocytes. (A) Immunocytochemistry images for p57 (red), GFAP (blue) and ßIII-tubulin (green) in NSCs after 7 DIV of diffe- rentiation in cultures that have been nucleofected with an overexpressing plasmid for *Cdkn1c* (Origene). An empty vector (EV) was used as a control for nucleofection. **(B)** Percentage of cells positive for ßIII-tubulin, Olig2 and GFAP in *Cdkn1c* overexpres- sing cells after 7 DIV in differentiation-promoting conditions. The percentage of undifferentiated cells is also determined. **(C)** Percentage of Ki67 positive cells in NSCs overexpressing *Cdkn1c* after 7 DIV of differentiation (left panel). Immunocytoche- mistry images for Ki67 (red) in NSCs overexpressing *Cdkn1c*. The empty vector condition is also shown (right panel). All error bars show s.e.m of 3 independent cultures. Scale bars in A and C, 30 μm (inserts in A, 10 μm).

© 2023. Published by The Company of Biologists under the terms of the Creative Commons Attribution License (https://creativecommons.org/licenses/by/4.0/).

Second decision letter

MS ID#: DEVELOP/2022/200563

MS TITLE: Igf2 interacts with the imprinted gene Cdkn1c to promote terminal differentiation of NSCs

AUTHORS: Anna Lozano-Urena, Laura Lazaro-Carot, Esteban Jimenez-Villaba, Raquel Montalban-Loro, Isabel Mateos-White, Irene Martinez-Gurrea, Pere Duart-Abadia, Keiichi I. Nakayama, Isabel Farinas, Martina Kirstein, Cristina Gil-Sanz, and Sacri R Ferron
ARTICLE TYPE: Research Article

I am delighted to tell you that your manuscript has been accepted for publication in Development, pending our standard ethics checks.

Reviewer 2

Advance summary and potential significance to field

The added in vivo and in vitro experiments (especially the primary cell cultures of NSC) strengthened the major conclusions of the manuscript. This article improves our understanding how mechanisms of how IGF2 regulates biology and cell dynamics of adult NSCs. The findings are relevant both to the adult neurogenesis and the IGF fields.

Comments for the author

The authors diligently and extensively addressed all major concerns and majority of minor concerns. I recommend this manuscript to publication if other reviewers are also satisfied with the way their concerns were addressed.

© 2023. Published by The Company of Biologists under the terms of the Creative Commons Attribution License (https://creativecommons.org/licenses/by/4.0/).
